# Phylogenetic Signal in Primate Tooth Enamel Proteins and its Relevance for Paleoproteomics

**DOI:** 10.1093/gbe/evaf007

**Published:** 2025-01-21

**Authors:** Ricardo Fong-Zazueta, Johanna Krueger, David M Alba, Xènia Aymerich, Robin M D Beck, Enrico Cappellini, Guillermo Carrillo-Martin, Omar Cirilli, Nathan Clark, Omar E Cornejo, Kyle Kai-How Farh, Luis Ferrández-Peral, David Juan, Joanna L Kelley, Lukas F K Kuderna, Jordan Little, Joseph D Orkin, Ryan S Paterson, Harvinder Pawar, Tomas Marques-Bonet, Esther Lizano

**Affiliations:** Département de sciences biologiques, Université de Montréal, Montréal, QC, Canada; Department of Medicine and Life Sciences, Institute of Evolutionary Biology (CSIC-UPF), Pompeu Fabra University, Barcelona, Spain; Department of Medicine and Life Sciences, Institute of Evolutionary Biology (CSIC-UPF), Pompeu Fabra University, Barcelona, Spain; Institut Català de Paleontologia Miquel Crusafont (ICP-CERCA), Universitat Autònoma de Barcelona, Edifici ICTA-ICP, Cerdanyola del Vallès, Barcelona, Spain; Unidad de Paleobiología, ICP-CERCA, Unidad Asociada al CSIC por el IBE UPF-CSIC, Barcelona, Spain; Institut Català de Paleontologia Miquel Crusafont (ICP-CERCA), Universitat Autònoma de Barcelona, Edifici ICTA-ICP, Cerdanyola del Vallès, Barcelona, Spain; School of Science, Engineering and Environment, University of Salford, Manchester, UK; Geogenetics Section, Globe Institute, University of Copenhagen, Copenhagen, Denmark; Department of Medicine and Life Sciences, Institute of Evolutionary Biology (CSIC-UPF), Pompeu Fabra University, Barcelona, Spain; Institut Català de Paleontologia Miquel Crusafont (ICP-CERCA), Universitat Autònoma de Barcelona, Edifici ICTA-ICP, Cerdanyola del Vallès, Barcelona, Spain; Department of Biological Sciences, University of Pittsburgh, Pittsburgh, PA, USA; Ecology and Evolutionary Biology, University of California Santa Cruz, Santa Cruz, CA, USA; Illumina Artificial Intelligence Laboratory, San Diego, CA, USA; Department of Medicine and Life Sciences, Institute of Evolutionary Biology (CSIC-UPF), Pompeu Fabra University, Barcelona, Spain; Department of Medicine and Life Sciences, Institute of Evolutionary Biology (CSIC-UPF), Pompeu Fabra University, Barcelona, Spain; Ecology and Evolutionary Biology, University of California Santa Cruz, Santa Cruz, CA, USA; Illumina Artificial Intelligence Laboratory, San Diego, CA, USA; Department of Human Genetics, University of Utah, Salt Lake City, UT, USA; Département de sciences biologiques, Université de Montréal, Montréal, QC, Canada; Department of Medicine and Life Sciences, Institute of Evolutionary Biology (CSIC-UPF), Pompeu Fabra University, Barcelona, Spain; Département d’anthropologie, Université de Montréal, Montréal, QC, Canada; Geogenetics Section, Globe Institute, University of Copenhagen, Copenhagen, Denmark; Department of Medicine and Life Sciences, Institute of Evolutionary Biology (CSIC-UPF), Pompeu Fabra University, Barcelona, Spain; Department of Medicine and Life Sciences, Institute of Evolutionary Biology (CSIC-UPF), Pompeu Fabra University, Barcelona, Spain; Institut Català de Paleontologia Miquel Crusafont (ICP-CERCA), Universitat Autònoma de Barcelona, Edifici ICTA-ICP, Cerdanyola del Vallès, Barcelona, Spain; Catalan Institution of Research and Advanced Studies (ICREA), Barcelona, Spain; Centro Nacional de Análisis Genómico (CNAG), Barcelona, Spain; Department of Medicine and Life Sciences, Institute of Evolutionary Biology (CSIC-UPF), Pompeu Fabra University, Barcelona, Spain; Institut Català de Paleontologia Miquel Crusafont (ICP-CERCA), Universitat Autònoma de Barcelona, Edifici ICTA-ICP, Cerdanyola del Vallès, Barcelona, Spain; Unidad de Paleobiología, ICP-CERCA, Unidad Asociada al CSIC por el IBE UPF-CSIC, Barcelona, Spain

**Keywords:** ancient biomolecules, primate evolution, dental enamel, paleoproteomics, phylogenetic analysis

## Abstract

Ancient tooth enamel, and to some extent dentin and bone, contain characteristic peptides that persist for long periods of time. In particular, peptides from the enamel proteome (enamelome) have been used to reconstruct the phylogenetic relationships of fossil taxa. However, the enamelome is based on only about 10 genes, whose protein products undergo fragmentation in vivo and post mortem. This raises the question as to whether the enamelome alone provides enough information for reliable phylogenetic inference. We address these considerations on a selection of enamel-associated proteins that has been computationally predicted from genomic data from 232 primate species. We created multiple sequence alignments for each protein and estimated the evolutionary rate for each site. We examined which sites overlap with the parts of the protein sequences that are typically isolated from fossils. Based on this, we simulated ancient data with different degrees of sequence fragmentation, followed by phylogenetic analysis. We compared these trees to a reference species tree. Up to a degree of fragmentation that is similar to that of fossil samples from 1 to 2 million years ago, the phylogenetic placements of most nodes at family level are consistent with the reference species tree. We tested phylogenetic analysis on combinations of different enamel proteins and found that the composition of the proteome can influence deep splits in the phylogeny. With our methods, we provide guidance for researchers on how to evaluate the potential of paleoproteomics for phylogenetic studies before sampling valuable ancient specimens.

**Significance**

Ancient protein sequences from dental enamel have been successfully applied to infer phylogenetic relationships of extinct species. Post mortem degradation and a rather small proteome (∼10 proteins) limit the amount of molecular information that can be retrieved from ancient dental enamel. As a benchmarking experiment, we simulated ancient protein sequence data from high quality primate genomic data and compared the phylogenies that were derived from each dataset. Our results characterize the minimum amount of ancient sequence information that enables phylogenetic placement of ancient samples at least at family level, and highlight possible pitfalls of paleoproteomics applied to phylogenomics.

## Introduction

The survival of endogenous amino acids in fossils was demonstrated in the mid-20th century (Abelson 1954). More recently, access to protein sequence data from long deceased organisms has been achieved with the aid of mass spectrometry methods (Ostrom et al. 2000; Nielsen-Marsh et al. 2002; Cappellini et al. 2014). Since then, the field has grown to propose a set of standards (Hendy et al. 2018; Welker 2018; Hendy 2021; Warinner et al. 2022), and has proven to reliably determine sequence information from samples from as much as 14.8 million years ago (Ma) (Stolarski et al. 2023). The persistence of peptides for millions of years, even from temperate to warm environments, contrasts with the maximum biomolecule age of 2 million years from ancient DNA (aDNA) under permafrost conditions, which are considered ideal for DNA preservation (Kjær et al. 2022).

Despite post mortem degradation and often low protein abundance in the tissue (Castiblanco et al. 2015), scientists have started studying ancient proteomes from a phylogenetic perspective (Buckley 2013; Welker et al. 2015, 2019, 2020; Cappellini et al. 2019; Madupe et al. 2023). Given the abundance of tooth remains in the archaeological record, a considerable amount of paleoproteomic research has focused on tooth enamel (Cappellini et al. 2019; Dickinson et al. 2019; Welker et al. 2019; Froment et al. 2020; Welker et al. 2020; Nogueira et al. 2021; Madupe et al. 2023). Several protein fragments are persistent in mature enamel (Castiblanco et al. 2015). These protein fragments have been successfully used to infer the phylogenetic position of extinct taxa, such as the Pleistocene rhinoceros *Stephanorhinus*, and the extinct hominids *Gigantopithecus blacki* and *Homo antecessor* (Cappellini et al. 2019; Welker et al. 2020, 2019). However, these studies have also highlighted some of the current challenges of addressing phylogenetic analysis through ancient proteins. The most evident drawback is the limited amount of information due to the short length of the recovered peptides. In particular, the enamel proteome is rather small, comprising <15 proteins, which are further enzymatically degraded in vivo during enamel formation (Smith et al. 1989), and even more post mortem. To date, the combined length of recovered peptides from ancient enamelomes can range between 456 amino acids (Welker et al. 2019) and 1,014 amino acids (Welker et al. 2020). In addition, the peptides from the enamel proteome are not evenly recovered along the protein sequence (Welker et al. 2020), further limiting subsequent analyses.

Whole-genome sequencing can provide many more informative sites than a whole proteome ever could. This advance has led to a continuous refinement of molecular phylogenies and thus provides a robust reference against which to compare protein sequence-based phylogenies. Moreover, protein sequences can be bioinformatically predicted from nucleotide sequences. This enables us to infer protein sequences without the need to sequence the proteins directly.

**Fig. 1.**
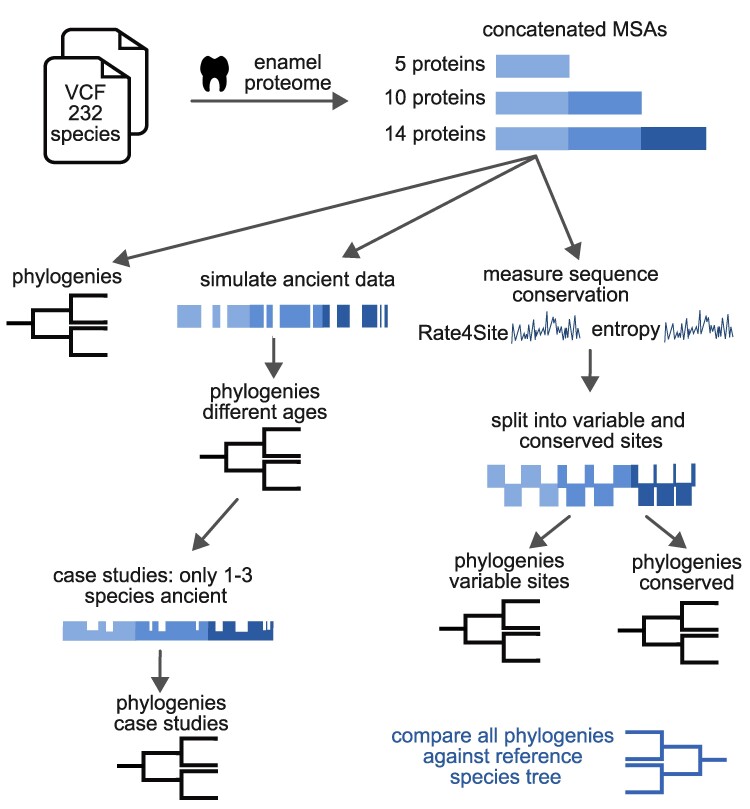
Overview of workflow. All primate genomic data stem from previously published VCF files (Meyer et al. 2012; Prado-Martinez et al. 2013; Prüfer et al. 2014, 2017; Xue et al. 2015; De Manuel et al. 2016; Mallick et al. 2016; Nater et al. 2017; Kuderna et al. 2023). From these files, we predicted the sequences of 14 tooth enamel proteins. The sequences of these proteins were aligned and concatenated into larger multiple sequence alignments (MSAs), combining different proteins. One version contains the 5 proteins that have been experimentally verified in several studies (Cappellini et al. 2019; Welker et al. 2019, 2020; Madupe et al. 2023), the 10 protein version contains 5 additional proteins that may be found in enamel, and the 14 protein version contains 4 additional collagen sequences. We performed phylogenetic analysis on the full sequence of these concatenated MSAs (excluding signal peptide). We also simulated ancient, fragmentary data for different degrees of fragmentation by eliminating sites in the MSA equivalent to data loss seen in ancient samples. Subsequently, we performed phylogenetic analysis on these MSAs, either with all or only 1–3 species fragmented. Moreover, we quantified the degree of variability of amino acid sites across primates using Shannon entropy and Rate4Site (Pupko et al. 2002). This way, sites of the MSAs could be categorized into “conserved” or “variable” and phylogenetic analysis could be performed on each of those sets of sites. All phylogenetic trees resulting from the analyses of this project were compared to a genomic data-based reference tree (Kuderna et al. 2023).

To our knowledge, a comprehensive assessment of the phylogenetic signal present in the enamel proteome has not been made thus far. Here, we evaluate the accuracy of phylogenetic reconstructions that can be achieved with fragmentary peptide data, compared to a robust, dated whole-genome phylogeny (Kuderna et al. 2023). We performed several phylogenetic analyses on protein sequences predicted from DNA data that span 16 families of the order Primates (Fig. 1). The analysis is based on 14 proteins that have been associated with the enamel proteome (Maas and Dumont 1999; Bartlett et al. 2006; Asaka et al. 2009; Zanolli et al. 2017; Cappellini et al. 2019; Welker et al. 2019; Welker et al. 2020; Madupe et al. 2023; Paterson et al. 2024). The proteins (or protein subunits) analyzed are alpha 2-HS glycoprotein (AHSG), albumin (ALB), ameloblastin (AMBN), amelotin (AMTN), amelogenin X-linked protein (AMELX), enamelin (ENAM), matrix metallopeptidase 20 (MMP20), odontogenic, ameloblast-associated protein (ODAM), serpin family C member 1 protein (SERPINC1), tuftelin 1 (TUFT1), collagen type I alpha 1 chain (COL1A1), collagen type I alpha 2 chain (COL1A2), collagen type XVII alpha 1 chain (COL17A1), and collagen type II alpha 1 chain (COL2A1). All predicted protein sequences were aligned. We assessed the degree of sequence conservation at each site of these alignments. Phylogenetic analysis was performed on the full-length translated sequences of the 14 enamel-associated proteins. A further analysis was carried out only with peptides corresponding to the protein regions typically captured in paleoproteomic studies, with the aim of simulating the limited amount of data in paleoproteomic studies. We further searched for segments in the protein sequences that appear most phylogenetically informative. The results of these analyses will inform future paleoproteomic studies by indicating which peptides should have priority in experimental recovery, but also by setting realistic expectations for the discriminatory power of these sequences in subsequent phylogenetic studies. Lastly, we discuss the implications of these findings and related factors, such as possible dependencies between the studied loci, when using ancient enamel peptides for evolutionary studies.

## Results

### Assessment of Protein Sequence Conservation

The degree of sequence conservation and evolutionary rates were examined for a set of 14 proteins from 232 primate species and 1 nonprimate outgroup (*Tupaia*). The analysis was performed on a concatenation of multiple sequence alignments (MSAs) of each protein into one large multiprotein MSA. Each species was represented by the individual that had the most complete sequence data (i.e. fewest gaps or masked positions).

**Fig. 2.**
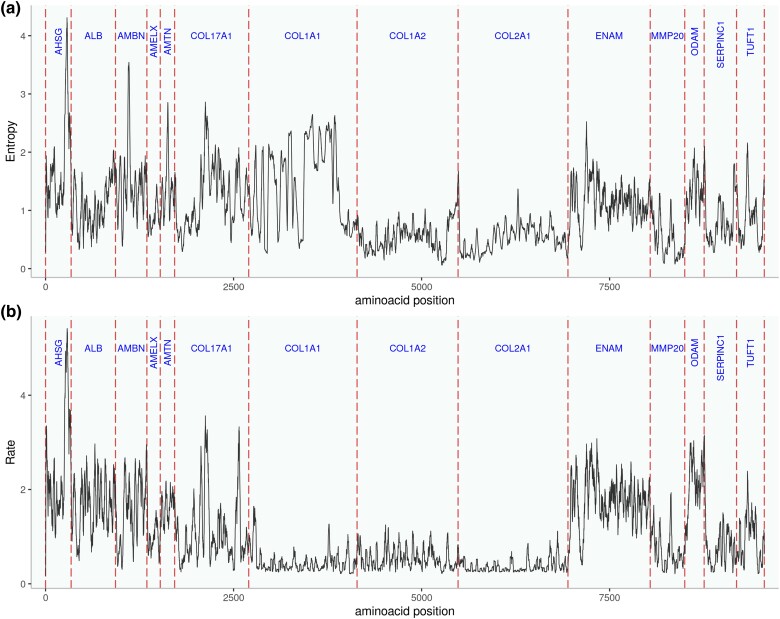
Evolutionary rates and sequence diversity estimated by a) Shannon entropy and b) Rate4Site scores for a concatenation of all 14 proteins. Collagens evolve at a slower rate than all noncollagen enamel proteins. COL17A1, which is the only collagen known to be an essential part of tooth enamel (Asaka et al. 2009), has an evolutionary rate and degree of conservation more similar to the noncollagen enamel proteins. COL1A1 displays elevated Shannon entropy values because many sequences have masked or missing positions that the software interprets as diversity.

**Fig. 3.**
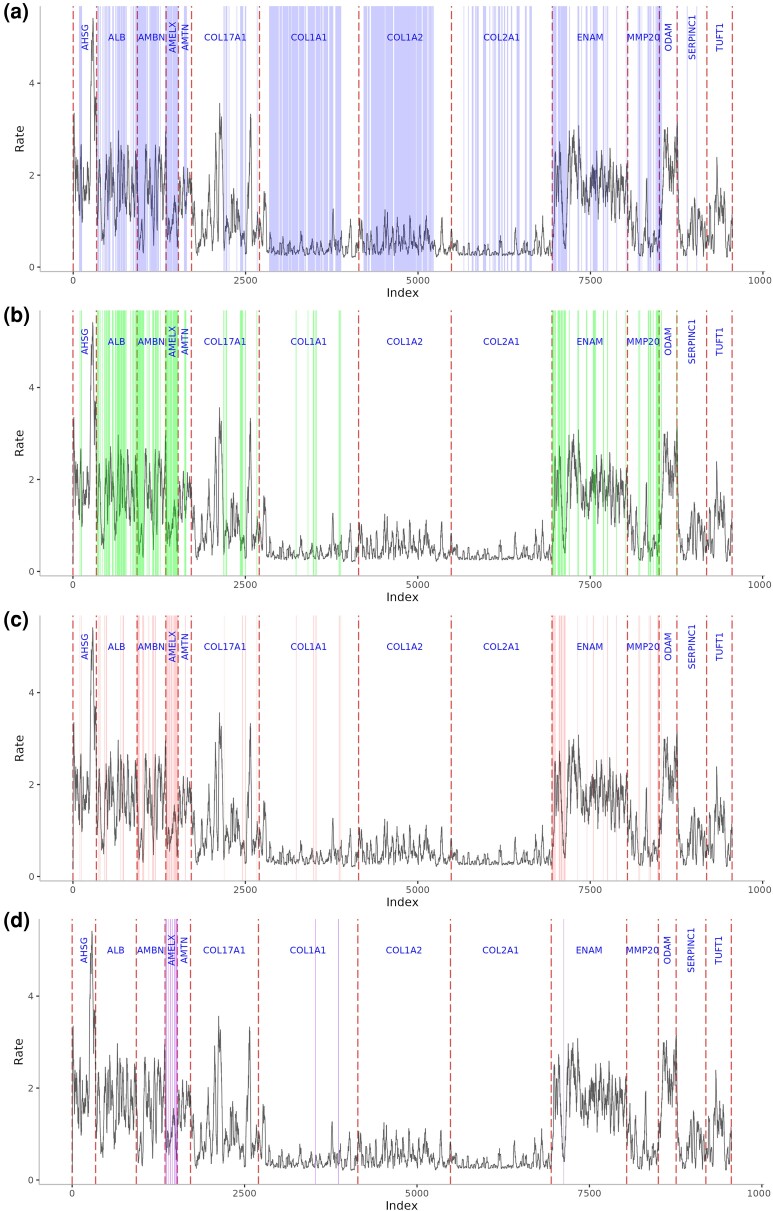
Evolutionary rates in the context of potential ancient sequence coverage across different time scales. The colorful shaded areas represent areas that have experimental support for being able to be retrieved from fossil tooth enamel samples. The names of the stages represent the ages of the samples they are based on, which all stem from moderate to tropical climate zones. They may not necessarily reflect the stage of degradation of any sample at this given age. a) “100 ka” (collagens may be retrieved from dentin or bone); b) “1 to 2 Ma”; c) “5 Ma” (no direct fossil evidence, extrapolated between “1 to 2 Ma” and “10 Ma”); d) “10 Ma.”

Both Shannon entropy and Rate4Site (R4S) can be applied to measure the degree of protein sequence conservation. While Rate4Site accounts for the different likelihoods of substitution during sequence evolution, Shannon entropy values are agnostic to any evolutionary or physico-chemical constraints. Shannon entropy values and Rate4Site scores both demonstrate that sequence diversity and evolutionary rates vary across the length of each protein sequence (Fig. 2). In particular, collagens (except some sites in COL17A1) evolve at a slower rate than noncollagen proteins (Fig. 2b). Rate4Site scores roughly correlate with Shannon entropy values (Fig. 2, Pearson correlation ρ = 0.54, *P*-value < 2.2e-16). About 4% of all sites are residues with particularly high evolutionary rates (R4S score > 2) that also fall into the regions that could be experimentally recovered in ancient samples (Fig. 3a). This is particularly the case in ALB, AMELX, AMBN, and ENAM. In most other proteins, such as AHSG, AMTN, COL17A, MMP20, ODAM, or TUFT1, the regions of particularly high evolutionary rates correspond to peptides that have not yet been experimentally recovered. At the other extreme, some regions of high sequence conservation levels stand out. In ENAM, there is a stretch of 49 highly conserved amino acids (corresponding to the positions 191 to 239 in UniProt ID Q9NRM1) that carries 2 phosphorylation sites. The region falls into the 32 kilodalton (kDa) cleavage product of ENAM (Ozdemir et al. 2005), which belongs to peptides that can be experimentally recovered in deep time (Fig. 3a-c) (Welker et al. 2020). MMP20 also displays larger regions of highly conserved amino acids that fall into the experimentally recovered sequences. One of those regions (corresponding to UniProt ID O60882, positions 174 to 254) lies around the active center (position 227) and its surrounding inorganic ion binding regions; another one lies around positions 330 to 483, a region whose ends are connected via a disulfide-bridge. Other longer experimentally recovered regions of relatively highly conserved sequences belong to AMBN, COL1A1, COL1A2, COL17A1, and SERPINC1.

To examine if the above described patterns also hold beyond primates, Rate4Site scores were calculated on a set of 22 species from different taxonomic groups across mammals (supplementary fig. S8, Supplementary Material online, for list of species see supplementary table S9, Supplementary Material online). The general pattern of slower evolutionary rates in collagens is consistent across mammals, particularly for COL1A1, COL1A2, and COL2A1. The area of the 32 kDa fragment in ENAM is not as strongly conserved as within primates; whereas, the region around the active center in MMP20 shows a persistently low evolutionary rate. Across mammals as a whole, the N-terminus of AMELX displays a higher degree of conservation, as is the case for primates specifically. In contrast, the C-terminus of AMTN, and the N-terminus of COL1A1 appear to evolve at a higher rate in the mammal-wide data compared to the primates-only data.

### Phylogenies Based on Full-Length Sequences

**Fig. 4.**
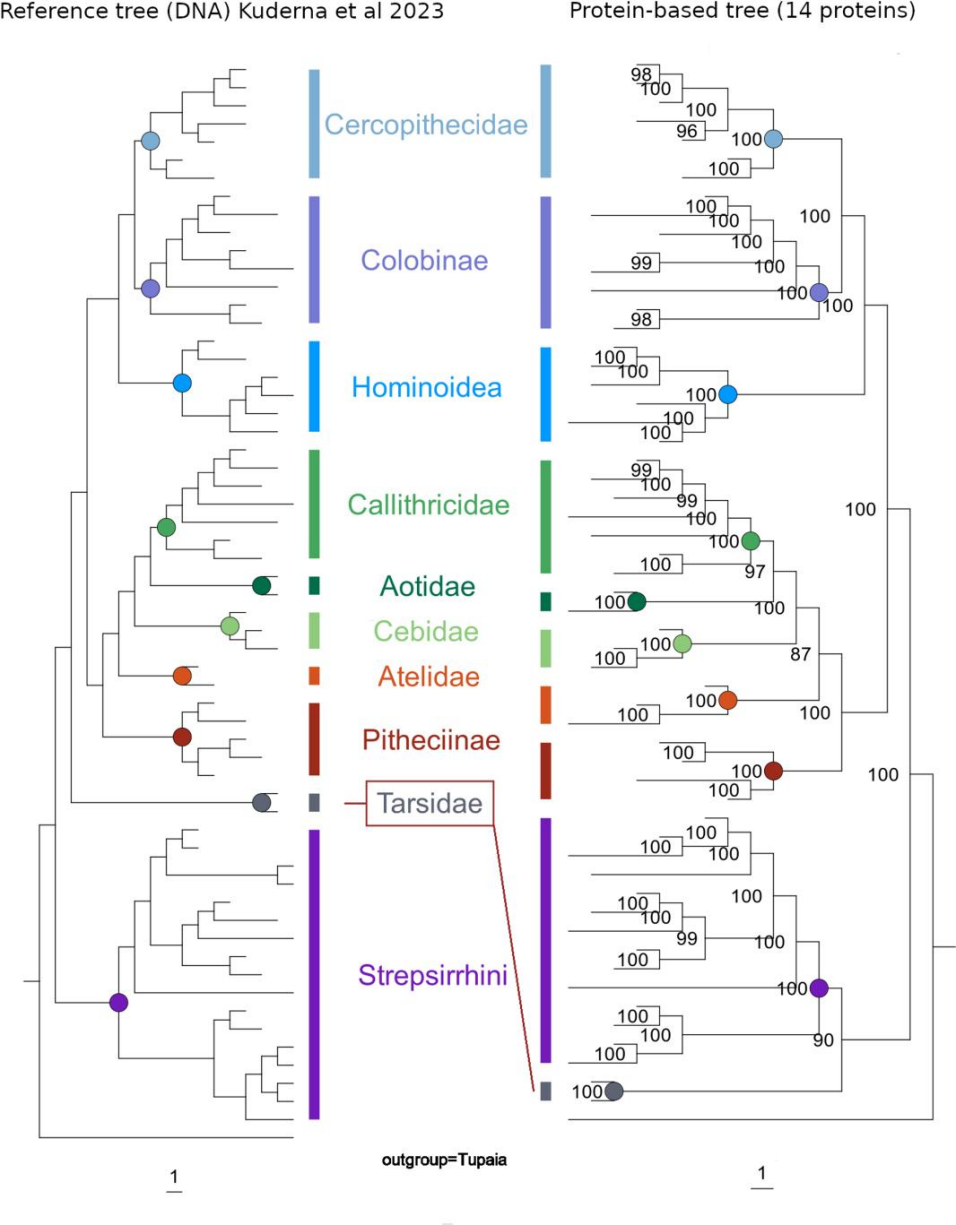
Species tree compared to phylogenetic tree based on 14 protein concatenation. The placement of families and even genera is largely in accordance. However, Tarsiiformes (only family Tarsiidae) form a monophyly with Strepsirrhini, a placement that is nowadays widely rejected (Hartig et al. 2013; Morse et al. 2019; Seiffert et al. 2020; Kuderna et al. 2023). In the phylogenies based on concatenations of 5 and 10 proteins, which do not comprise collagens, Tarsiidae are placed as a sister group of Simiiformes in accordance with the reference tree (Kuderna et al. 2023) (supplementary fig. S2, Supplementary Material online).

The phylogenetic signal in each protein sequence dataset was assessed by measuring the Robinson–Foulds distance (RF-distance, topology only) between the tree resulting from that dataset and the reference tree, as well as manual inspection of differences in the topologies. There was no major difference in the phylogenetic trees created by maximum likelihood (ML) or Bayesian analysis (supplementary fig. S3, Supplementary Material online). The Bayesian analysis performed slightly better by creating more accurate trees (smaller RF-distances to reference tree) from the 5 (ML = 170, Bayesian = 153) and 10 (ML = 122, Bayesian = 117) protein concatenations; however, the ML approach produced a slightly more accurate tree for the 14 (ML = 108, Bayesian = 110) protein concatenation. Since the differences between the methods appeared to be minor, all following analyses were performed using the ML approach because it shows a higher computational efficiency (supplementary table S4, Supplementary Material online). In all trees, all taxa were placed correctly at least at the family level, with 2 exceptions: First, in the tree based on the 5 protein concatenation, Galagonidae and Lorisidae remain unresolved, meaning that species of these 2 families do not form 2 distinct clades. Second, while the general tendency is that the more proteins that are included in the concatenation, the more similar the tree is to the reference tree, there is 1 caveat for the 14 protein concatenation: the deepest relationship within Primates, namely the branching pattern between lorises and lemurs (Strepsirrhini), tarsiers (Tarsiiformes), and monkeys and apes (Simiiformes), is incorrectly resolved. Specifically, in the trees of the 14 protein concatenation (ML and Bayesian), Tarsiiformes form a clade with Strepsirrhini to the exclusion of Simiiformes (Fig. 4) with a bootstrap value of 90 and a posterior probability of 1; however, current molecular and morphological evidence (Hartig et al. 2013; Morse et al. 2019; Seiffert et al. 2020; Kuderna et al. 2023) collectively provides compelling support for Tarsiiformes + Simiiformes to the exclusion of Strepsirhini. In contrast, in the phylogenies from the 5 and 10 protein concatenations, Tarsiiformes and Simiiformes form a clade, in agreement with the reference tree (supplementary fig. S2, Supplementary Material online).

In our case, the addition of 4 collagen genes to the dataset, resulting in the 14 protein concatenation, drove the misplacement of Tarsiiformes. We tested different combinations of collagens and noncollagen proteins of our dataset to see which gene products in particular are driving this misplacement (supplementary table S5, Supplementary Material online). If any of these individual collagens is combined with all 10 noncollagen proteins into an 11 protein concatenation, the noncollagen proteins drive the placement of tarsiers to the correct position, according to the species tree. However, if the 10 noncollagen proteins are combined with COL1A2 and COL1A1 or COL17A1, this is sufficient to override the signal in the noncollagen proteins and place Tarsiiformes with Strepsirrhini.

### Phylogenies by Fragmentation Stage

To date, there is only a limited amount of information on how enamel proteins degrade post mortem over large time scales. We created a simple model of peptide fragment degradation by inspecting publicly available experimental enamel proteomes, combined with newly sequenced enamel proteomes from this study. These experimentally recovered enamel proteome sequences were aligned to the MSAs of our predicted protein sequences (“BLOCK3” in supplementary file S2, Supplementary Material online). Different stages of post mortem protein sequence fragmentation were then modeled by removing specific sites (i.e. columns) in the MSA of each protein. The older the modeled fragmentation stage, the more sites were removed. For the model, we assumed heterogeneous post mortem survival times across all sites of each protein for 2 reasons: first, because the enamelome is already enzymatically cleaved in vivo (Smith et al. 1989), and second because different peptides may have different physico-chemical properties that influence their chemical breakdown. To understand the approximate patterns of this heterogeneity, we assessed how often each site in a protein could be experimentally recovered from published data together with new data using the MSAs. To date, the amount of available ancient protein sequences is not sufficient to use statistical methods to model the process of fragmentation across millions of years. Instead, we followed the rationale of reducing the amount of sequence information similar to what we observed in sequences of a certain age range. These ages give rise to the eponymous fragmentation stages. The more coverage a single site has, the longer is its anticipated survival.

**Fig. 5.**
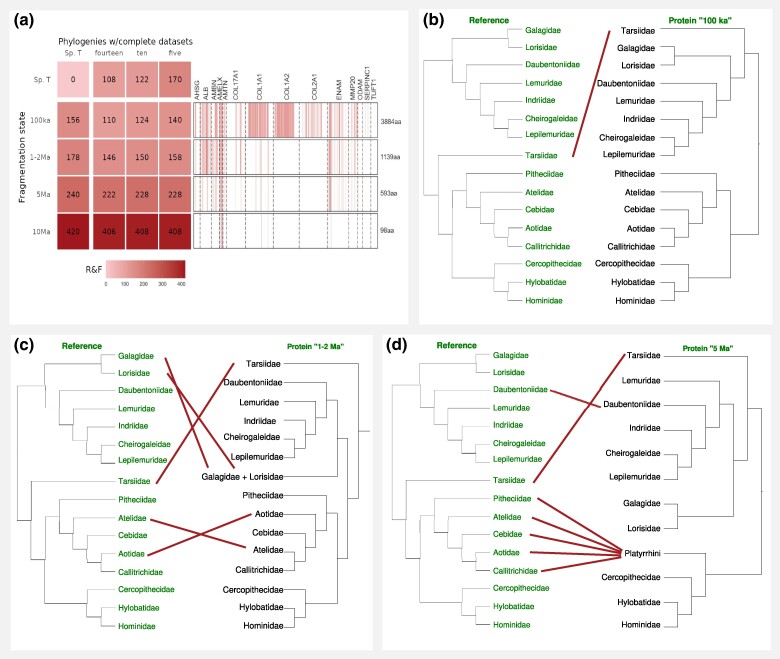
Phylogenies based on simulated ancient data and reference tree. a) Robinson–Foulds distances. Sp.T.—Reference tree based on whole-genome data (Kuderna et al. 2023), “100 ka,” “1 to 2 Ma,” “5 Ma,” and “10 Ma” represent different stages of fragmentation to which the amino acid MSA has been reduced prior to phylogenetic analysis. b) Differences at family level between reference tree and “100 ka” tree. c) Differences at family level between reference tree and “1 to 2 Ma” tree. d) Differences at family level between reference tree and “5 Ma” tree. The“10 Ma” tree is not shown because the family clades are widely lost.

Based on the modeled fragmentation stages, we created 4 concatenated MSAs with increasingly reduced sequence data and examined phylogenetic trees that were calculated from those MSAs in order to understand the standalone phylogenetic information of fragmented protein sequences. The protein concatenation corresponding to the fragmentation stage of “100 ka” had a total length of 3,884 amino acids (∼41% of the original MSA's length, see supplementary table S3, Supplementary Material online). The phylogeny based on this data showed a RF-distance of 156 to the species tree (Fig. 5), in contrast with the phylogeny based on the full-length 14 proteins (alignment length 9,557 amino acids), which showed a RF-distance to the species tree of 108. All placements at family level and mostly genus level are in accordance with the reference tree, except for Tarsiiformes being grouped with Strepsirrhini (for more details see supplementary table S10, Supplementary Material online). This is in line with the previous observation that the inclusion of COL1A2 and COL1A1 or COL17A1 can produce this result, even if just fragments of these protein sequences are included. Most differences that explain the RF-distance of 156 stem from different placements of species within their genus. The phylogeny based on the fragmentation stage “1 to 2 Ma” (MSA length 1,139 aa, ∼12% of the original MSA's length) has a very similar distance to the species tree (158). Most nodes at family level are placed in accordance with all confidently resolved nodes of the reference tree, with some exceptions (supplementary table S10, Supplementary Material online). For instance, in Hominidae, contrasting the reference tree, *Pan* and *Gorilla* are sister taxa, with *Homo* as an outgroup. Tarsiiformes are placed as an outgroup to both Strepsirhini and Simiiformes (bootstrap 100), a placement that is rejected by current molecular and morphological evidence (Hartig et al. 2013; Morse et al. 2019; Seiffert et al. 2020; Kuderna et al. 2023).

The tree based on data of the fragmentation stage “5 Ma” (alignment length 593 amino acids, ∼6% of the original MSA's length) has an RF-distance to the species tree of 240. In catarrhines, all family level relationships still agree with the reference tree, but there are some inconsistencies within families compared to the reference tree (supplementary table S10, Supplementary Material online). In platyrrhines, correct resolution of nodes at family level is widely lost. The placement of nodes at family level in Lemuridae is in accordance with the species tree, except for the placement of *Varecia* and *Daubentonia madagascariensis* (supplementary table S10, Supplementary Material online). Tarsiiformes form a clade with Strepsirhini (bootstrap 93).

At the fragmentation stage “10 Ma” (alignment length 98 amino acids, ∼1% of the original MSA's length), the phylogeny is largely unresolved, with an RF-distance to the reference tree of 420 and most bootstrap values far below 50. Only lorisiforms are correctly separated as their own taxon. The 4 tarsiiform species are monophyletic and placed with low confidence within Simiiformes.

### Case Studies of Simulated Ancient Samples

**Fig. 6.**
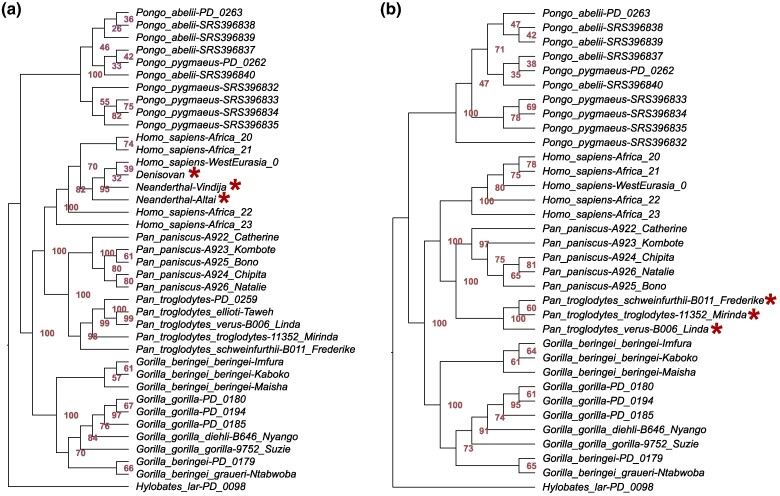
Phylogenetic trees of simulated cases studies of Neanderthals and chimpanzees. Only the sequences of the tested species (marked with asterisk) were fragmented and aligned together with full-length sequences of the reference. a) “Neanderthal case,” sequences of Neanderthal (*Homo neanderthalensis*) and Denisovan individuals were in silico fragmented to fragmentation stage “100 ka.” In the resulting phylogeny, they cannot be distinguished from anatomically modern humans. b) “Chimpanzee case,” sequences of chimpanzee (*P. troglodytes*) were in silico fragmented to fragmentation stage “5 Ma.” In accordance with the reference tree, they form a sister group to all bonobos (*Pan panicus*).

Often, paleoproteomic studies will only aim to place a few closely related specimens at a time into a framework of mostly complete reference protein sequences. To simulate such a scenario, we created 4 cases in which protein sequence data from a group with fairly well-known taxonomic placement were fragmented and aligned to reference sequences that are at full-length. In the first case, sequence data of 2 Neanderthals (individuals Vindija 33.19 and Altai) and 1 Denisovan (Denisova3 individual) that were fragmented to the degradation stage “100 ka” were aligned to a reference MSA of 14 enamel-related proteins from Hominoidea. The phylogenetic placement is in accordance with the reference tree at genus level, but not at species level (Fig. 6a). The Neanderthal and Denisovan individuals place within the *Homo sapiens* clade with low branch support, instead of forming a sister clade as shown by large-scale genomic data (Kuhlwilm et al. 2016). In the same tree, individuals of the 2 species with the youngest split, namely *Pongo abelii* and *Pongo pygmaeus*, do not form 2 distinct clades, nor do the individuals of *Gorilla gorilla* and *Gorilla beringei*, despite being based on full-length protein data. These results align with previous observations on the limitations of paleoproteomic data to resolve phylogenies in Hominidae at species-level resolution (Welker et al. 2019, 2020; Madupe et al. 2023). In particular, if several individuals per clade are examined using such limited sequence data; the interspecific differences can fall within the range of intraspecific variation (Madupe et al. 2023). Reasons for this low phylogenetic resolution between species of the same genus can be incomplete lineage sorting or the overall slow evolutionary rate of the proteins found in dental enamel.

**Fig. 7.**
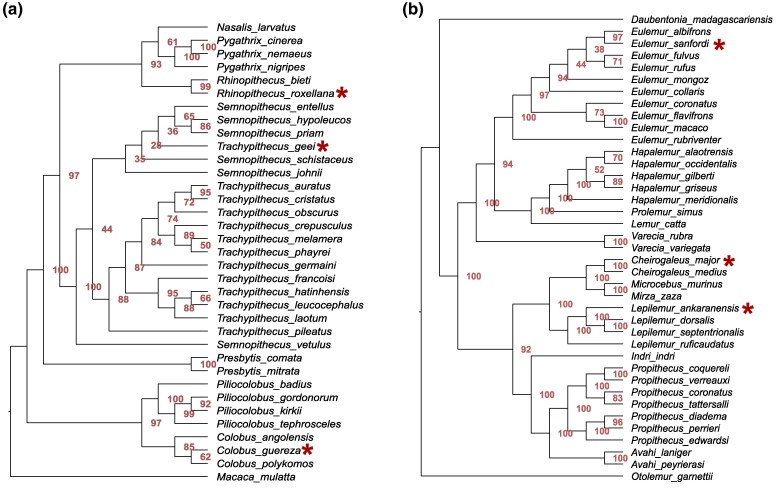
Phylogenetic trees of simulated cases studies of colobines and lemurs. Only the sequences of the tested species (marked with asterisk) were fragmented and aligned together with full-length sequences of the reference. a) “Colobine case,” sequences of *T. geei*, *R. roxellan*a, and *C. guereza* were in silico fragmented to fragmentation stage “5 Ma.” Note that *T. geei* is not placed correctly at genus level. This can be fixed by allowing for mixture models (supplementary fig. S9, Supplementary Material online). *T. geei* can also be placed correctly at genus level if the topology of all reference sequences is constrained. Constraining the topology also fixed the wrong placement of the reference individual from the species *Semnopithecus vetulus* (supplementary fig. S10, Supplementary Material online). b) “Lemur case,” sequences of *E. sanfordi*, *C. major*, and *L. ankaranensis* were in silico fragmented to fragmentation stage “100 ka.” All of them are placed in the clade of their respective genus.

The example of simulated ancient chimpanzee data of a fragmentation stage of “5 Ma” produces a different result (Fig. 6b). While the phylogenetic relationships of the species within *Pongo* and *Gorilla* cannot be resolved, the 3 *Pan troglodytes* individuals are placed confidently in a clade that is a sister taxon to *Pan paniscus* (bootstrap 100). Divergence times have been estimated using MCMCtree for each node of a simplified version (1 individual per species) of the “Chimpanzee case” tree (supplementary table S11 and figs. S11 and S12, Supplementary Material online). The confidence intervals for the estimates of the case study and the reference tree (Kuderna et al. 2023) overlap at nearly all nodes. On average, divergence time estimates derived from the protein-based data of the “Chimpanzee case” are 21% younger than those of the genome-based reference. While this is only one case example, it shows that divergence time estimates with similar results to those derived from high quality genome data are possible. In the “Colobine case” (Fig. 7a), simulated ancient samples (fragmentation stage “5 Ma”) of *Rhinopithecus roxellana*, and *Colobus guereza* are placed correctly at species level. However, the simulated ancient sample of *Trachypithecus geei* is placed within its sister genus *Semnopithecus*. Slight modifications of the methodology can fix this misplacement. *T. geei* was placed within its correct genus, when mixture models were allowed (supplementary fig. S9, Supplementary Material online). It was also placed correctly when the topology of the reference data was constrained based on information from the DNA-based reference tree (Kuderna et al. 2023) (supplementary fig. S10, Supplementary Material online). In the “Lemur case” (Fig. 7b), the simulated ancient samples (fragmentation stage “100 ka”) of *Eulemur sanfordi* and *Cheirogaleus major* were placed correctly at species level and *Lepilemur ankaranensis* at genus level.

### Phylogenies by Amino Acid Conservation

To quantify the contribution of the variable sites in each MSA to the correct resolution of the according tree, we divided all concatenated MSAs into 2 MSAs, 1 consisting of variable sites, the other of conserved sites. To divide between variable and conserved, the MSA of each protein was normalized to a mean of 1 in their Shannon entropy values or Rate4Site scores. They were then divided into sites above this value, i.e. the “variable” sites, and below, i.e. the “conserved” sites (supplementary fig. S6, Supplementary Material online). These sites from all proteins were concatenated and used for phylogenetic analysis with ML. Phylogenies based only on variable sites identified by Rate4Site (supplementary fig. S7, Supplementary Material online, “R4S variable”) are just as similar to the species tree as in the case of phylogenies based on the full-length protein sequences (supplementary fig. S3, Supplementary Material online, both have a RF-distance of 108 to the reference tree). Similarly, when using Shannon entropy to define conserved and variable sites, phylogenies based on the variable sites (supplementary fig. S7, Supplementary Material online, “Shannon variable”) are more similar to the reference tree than those based on the more conserved sites (“Shannon conserved”). In general, all phylogenies based on variable sites that were identified using Rate4Site (“R4S variable”) are more similar to the reference tree than those based on variable sites that were identified using Shannon entropy (“Shannon variable”). Thus, the more variable sites contain most of the phylogenetic signal. In this case, to define the most variable sites, Rate4Site has been a better predictor of phylogenetically informative sites.

### Evolutionary Rate Covariation Scores

**Fig. 8.**
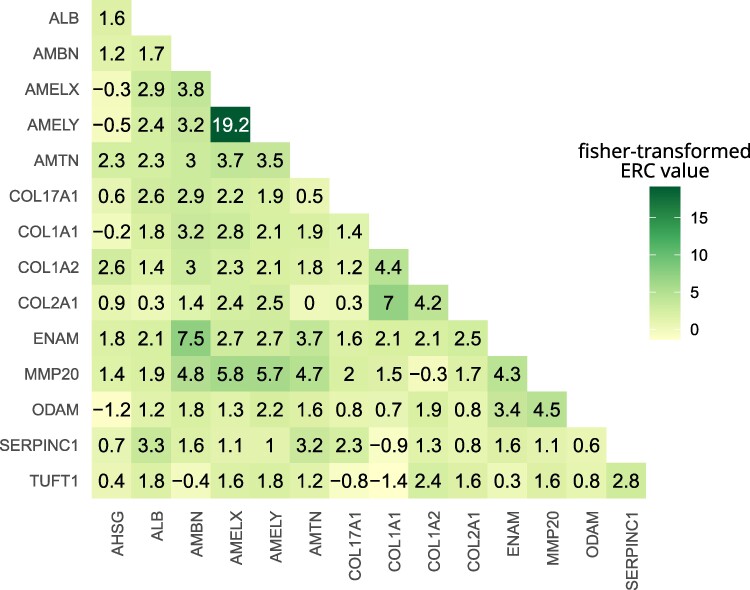
Fisher-transformed ERC values between the 14 proteins of this study. The strongest correlation of evolutionary rates was measured between *AMELX* and *AMELY*. Elevated ERC values can also be observed between the gene that encodes the enzyme MMP20 and its cleavage targets AMBN, AMELX, AMELY, and ENAM (Gasse et al. 2017). Also between the nonenamel collagens *COL1A1*, *COL1A2*, and *COL2A1*, elevated ERC values can be observed.

The enamel proteome is functionally linked, in particular because all proteins are expressed only during amelogenesis (formation of enamel) during a very short phase of an individual's development (Castiblanco et al. 2015). As a possible consequence of this, the evolution of their genes may occur in a nonindependent manner. We estimated the degree of evolutionary covariation of the set of 14 genes using Evolutionary Rate Covariation (ERC) analysis (Clark et al. 2012). ERC returns pairwise correlation coefficients of the branch-specific evolutionary rates of a set of genes (Fig. 8). All ERC scores are based on comparisons between phylogenetic trees that were derived from a representative dataset of coding sequence alignments produced from whole-genome alignments of 120 mammalian species (Hecker and Hiller 2020). Permutation testing indicated that all pairwise values between amelogenesis proteins are significantly elevated (*P*-value < 0.0001). There is particularly elevated covariation (Fisher-transformed value > 3) in the evolutionary rates of all pairs between *COL1A1*, *COL1A2*, and *COL2A1*, whose protein sequences are 64% to 72% identical. *COL17A*, the most divergent of all of the collagens and the only one known to have a function in enamel formation, displays lower covariation with the other collagens.

*AMBN*, *AMTN*, *ENAM*, and *ODAM* are located on a syntenic block (e.g. *Homo sapiens*, chr 4; *P. troglodytes*, chr 4; *Microcebus murinus*, chr 26, *Mus musculus*, chr 5) in the SCPP (secretory calcium-binding phosphoprotein) gene cluster (Sire et al. 2007). Together with *AMELX*, they all evolved through duplications from the ancestral vertebrate *SPARC* gene and resemble each other in gene structure and chemical protein properties (Sire et al. 2007). The elevated and significant ERC values between them may reflect the evolutionary, functional, and spatial connection of these genes.

One of the genes with the highest pairwise correlation values is *MMP20*, a gene that encodes for an enzyme that cleaves the proteins AMBN, AMELX, AMELY, and ENAM during amelogenesis (Gasse et al. 2017). All genes encoding those cleavage targets display elevated values of covariation of evolutionary rates. *AMTN* is another gene that displays a higher correlation in evolutionary rates with the aforementioned group, but little is known about its interactions and function. The most striking degree of covariation (Fisher-transformed value = 19.2) can be observed between *AMELX* (chromosome X) and *AMELY* (chromosome Y). For being encoded on the sex chromosomes they can be considered paralogs. It is known that both are expressed, if a Y chromosome is present, and *AMELX* and *AMELY* seem to fulfill the same function (Haruyama et al. 2011; Parker et al. 2019).

In summary, many enamelome genes display significant degrees of covariation in their evolutionary rate, which suggests evolutionary nonindependence. Thus, the inference of a species tree from these nonindependent loci may result in a tree that reflects the shared evolutionary history of the loci rather than the actual evolution of the species (Pamilo and Nei 1988; Spinks et al. 2009; Lozano-Fernandez 2022).

## Discussion

In this study, we estimated the degree of sequence conservation, evolutionary rate, and phylogenetic signal of protein sequences that are associated with the primate enamel proteome. Our analyses emphasized evaluating these metrics from a perspective of experimental feasibility, since ancient peptide data are highly fragmentary and diagenetically altered (e.g. by deamidation, Ramsøe et al. 2020). The process of degradation was simulated for different stages of fragmentation, which were anticipated from experimental data. Given the limited amount of experimental data and the over-representation of samples younger than 2 Ma, it has not yet been possible to statistically assess the patterns of post mortem sequence degradation, but hopefully it will be in the future as more ancient enamel peptide sequences are published. However, patterns are already visible, e.g. the deep time sequence survival of N-terminal peptides of ENAM (Fig. 3), and that of the N-terminal region of AMELX and its C-terminal proline rich region (Fig. 3).

When simulating fragmented data and subsequently performing phylogenetic inference, most families were in accordance with the reference tree up to a fragmentation stage similar to published samples of an age of 1 to 2 Ma from temperate-to-tropical regions. This does not exclude that sequences with a higher degree of fragmentation (stage “5 Ma”) could be placed correctly in a phylogeny that is based on unfragmented reference sequences, as the *P. troglodytes* sequences in our case study were rather highly fragmented (stage “5 Ma”) and still correctly placed. The same was true for equally fragmented sequences of colobine species.

The effects of missing data on phylogenetic analysis have been explored before (Wiens and Morrill 2011; Roure et al. 2013), but it would also be interesting to further study how to best address missing data in the particular case of ancient enamelomes. Comparing the case studies with only a few fragmented sequences to the phylogenies based on exclusively fragmented data, we could observe that, in phylogenetic analysis, more fragmentation can be tolerated if it affects only some sequences of the MSA. Thus, the conclusions drawn from the phylogenies based on exclusively fragmented data can be considered a conservative consideration, which draws a baseline for expectations. Understanding the use of many fragmentary sequences at a time can also be relevant for future studies, when more paleoproteomic data are available and compared to each other.

Rate4Site identified well the sites that are most phylogenetically informative (supplementary fig. S7, Supplementary Material online). This falls within the expectation, since Rate4Site accounts for amino acid replacement models and phylogenetic relationships between the input sequences. However, informative sites do not always fall within those regions that could be experimentally recovered, e.g. in AHSG, AMTN, COL17A, MMP20, ODAM, or TUFT1 (Fig. 3).

It may be possible to adapt protocols for peptide isolation from tooth enamel in order to maximize the yield of phylogenetically informative sequences. Some progress has been achieved recently by fractionating the sample in order to recover more fragments of different hydrophobicity (Madupe et al. 2023). Identifying variable sites in collagens may also be of interest for optimizing protocols for the application of ZooMS (Buckley et al. 2009; Naihui et al. 2021). The general pattern of conservation of individual sites can also be observed across mammals (supplementary fig. S8, Supplementary Material online). Some cases distinguish primates from the general trend in mammals, e.g. the 32 kDa fragment of ENAM appears particularly conserved in primates. In fact, in this region, signals of positive selection have been reported in primates (Al-Hashimi et al. 2009). This indicates that the degree of sequence conservation might differ across clades.

Evolution of a set of proteins from a specialized tissue may be tightly linked due to the constraints of morphology and function of this tissue. Our example of tarsiers underlines why working with such a small tissue-specific set of biological sequences should be accompanied by morphometric and histological expertise. For example, compared to Simiiformes, Tarsiiformes and Strepsirrhini share the traits of thinner tooth enamel (Shellis et al. 1998), and similar enamel microstructure (Maas and Dumont 1999). Both may be reflected in a similar genetic basis, e.g. as a conserved ancestral trait or as a result of convergent evolution.

Beyond the morpho-functional constraints, the relationships between such a small set of genes can be further entangled, as this is the case for *AMBN*, *AMTN*, *ENAM*, and *ODAM*, which are located in close proximity to each other on the same chromosome in most mammals (e.g. *Homo sapiens*, chr 4). This has also been reflected in significantly higher ERC values in our analysis (Fig. 8) and partly observed in another study that was able to associate evolutionary rates of *ENAM* and *ODAM* to enamel thickness (Mu et al. 2021). A third aspect of covariation and possible codependence of this set of typically studied genes is high sequence similarity between some of them. For instance, all collagens in this study share 38% to 72% sequence identity among each other in humans (aligning UniProt entries P02452, P08123, P02458, and Q9UMD9). We did not have sufficient genomic data to include the Y-chromosomal *AMELX* paralog, *AMELY*, into our analyses based on predicted protein sequences. It is known to share around 88.5% sequence similarity with AMELX in humans (aligning UniProt entries Q99217-3 and Q99218-2) and it showed by far the highest degree of covariation in the ERC analysis. In other mammals, signs of gene conversion between *AMELX* and *AMELY* have been reported, indicating that these 2 genes and their protein sequences are not acting as independent loci (Janečka et al. 2018; Kawasaki et al. 2020).

The dependencies that exist within this small proteome challenge the practice of concatenating them into a single, long MSA to address phylogenetic questions, because an overrepresented set of dependent loci might skew the outcome toward their shared evolutionary history. Gene trees can differ from species trees for various reasons (Pamilo and Nei 1988; Maddison 1997): Especially in cases of deep coalescence, i.e. when the common ancestry of a set of gene copies from different species extends further back than the speciation events, the lineage sorting of the genes does not necessarily agree with the splits between species. Also gene duplication and loss events can lead to discordance between species tree and gene tree because (sometimes unknowingly) the genes considered are in reality paralogs. Thus, the sampling of a reasonably high number of unlinked loci can help to mitigate the impact of discordant gene trees by reducing their stochastic impact (Pamilo and Nei 1988; Spinks et al. 2009; Lozano-Fernandez 2022). Differences between gene trees and the species tree may be a possible explanation for the case of tarsiers, which were placed in profoundly different locations within the phylogenetic tree depending on whether or not collagens were included in the dataset (Fig. 4; supplementary table S5, Supplementary Material online). However, we cannot rule out other causes, such as model misspecification (supplementary information, section S2.4, Supplementary Material online), i.e. the collagen sequences may have evolved in a way that cannot be appropriately modeled by the phylogenetic model used in our analysis (Jermiin et al. 2020). This case example highlights a potential pitfall of paleoproteomics when used for phylogenetic analysis. The specimen of interest might be placed with a reasonably high confidence in a phylogeny based on concatenated protein sequences, as for example tarsiers were placed with Strepsirrhini at the “100 ka” fragmentation stage. Still, as this example demonstrates, such a placement could be in conflict with genomic evidence, and yet there may be no genomic data for the ancient sample that can be used to test the accuracy of the proteomic data.

The difference between single-gene trees and species trees in the context of the enamel proteome has been demonstrated and discussed in previous publications (Welker et al. 2019, 2020; Spoutil et al. 2023), in which phylogenetic inference based on concatenated MSAs delivered results that were more consistent with the recognized species tree for the verifiable extant reference taxa. A common approach for deriving a species tree from a set of gene trees is the multispecies coalescent (Duchêne 2021; Douglas et al. 2022). A multispecies coalescent approach (Douglas et al. 2022) was compared to several Bayesian and maximum likelihood tools in the study of ancient proteins from *Paranthropus robustus*, where the results did not differ significantly from the other approaches (Madupe et al. 2023). Even with optimal phylogenetic tools, it still condenses to making an adequate choice of proteins to be considered (e.g. for studying ancient tarsiers it may be advisable to omit COL1A1). Yet, omission of sequences is costly and needs to be well-justified, when sequence information is scarce.

Altogether, our results provide several lessons for future paleoproteomic studies, in particular on dental enamel: Generally, the genetic distinction of species of the same genus is not possible with the sequences of the enamelome, even with complete sequence data. Consequently, ancient enamelome sequence studies are most likely inadequate to resolve research questions of the phylogenetic relationship between species of the same genus. An exception to this rule of thumb may be justified, if the divergence between the species is relatively deep, such as this was the case in our study of the relationship between chimpanzees and bonobos.

We observed that a small set of loci can have the potential to affect the position of deep splits in the phylogeny and produce wrong results that nevertheless have high statistical confidence (e.g. high bootstrap values in the wrong placement of tarsiers with strepsirrhines). For any specimen that is of interest for paleoproteomic study, we advise to gather protein sequences of the closest related extant species, prior to sampling it. A phylogenetic analysis of these species may reveal the expected phylogenetic resolution of the enamelome in this particular clade and if the protein sequences of some loci can cause unexpected results.

Lastly, we could observe that there is a degree of sequence fragmentation beyond which meaningful phylogenetic inference is impossible (between 1% and 6% of the whole enamelome, between fragmentation stages “5 Ma” and “10 Ma”). It is difficult to anticipate the expected yield of a sample solely based on its estimated age. In particular, temperature is a main driver for protein degradation, with lower temperatures being much more favorable for slowing down this process (Cappellini et al. 2019; Welker et al. 2019). An approach to estimate the expected protein preservation in a specimen of interest is to conduct pilot studies on more abundant specimens from other mammalian species that were ideally found in a comparable paleontological context at the same locality (Welker et al. 2020; Madupe et al. 2023).

Our results and conclusions help evaluate whether a specimen is an adequate candidate for a paleoproteomics-based phylogenetic study, providing guidance on the expected outcome. More publicly available experimental paleoproteomic data will hopefully contribute to refining this picture of the potential of paleoproteomics for phylogenetic applications.

## Materials and Methods

### Genes of Interest

The proteins analyzed are the products the genes *AHSG, ALB, AMBN, AMTN, AMELX, ENAM, MMP20, ODAM, SERPINC1, TUFT1, COL1A1, COL1A2, COL17A1,* and *COL2A1*. The selection of these proteins was mainly driven by the availability of experimental proteomic and genomic data. Other proteins associated with tooth enamel, such as KLK4, may play a key role in enamel formation (Yamakoshi et al. 2006), but barely leave behind any peptides that can be experimentally recovered in paleoproteomic studies (Cappellini et al. 2019; Welker et al. 2019; Welker et al. 2020; Madupe et al. 2023). Similarly, *AMELY* is considered enamel-specific, but since it is encoded on the difficult-to-sequence Y chromosome, there is little genomic reference data available. Although their gene products are not canonically considered to be part of tooth enamel, *COL1A1*, *COL1A2*, and *COL2A1* have been included in this study, because they are occasionally co-extracted from dentin fragments still attached to ancient enamel samples processed for paleoproteomic analysis (Madupe et al. 2023), or because they are recovered in experiments targeting bone or dentin on younger fossils (Welker et al. 2015; Chen et al. 2019; Presslee et al. 2019). Lastly, these collagens are of great importance for the peptide mass fingerprinting method conventionally called “zooarchaeology by mass spectrometry” or “ZooMS” for short (Buckley et al. 2009; Naihui et al. 2021).

### Dataset

The primate DNA sequences stem from 718 Variant Calling Format files (VCFs) from whole-genome sequence data, which were analyzed along with publicly available DNA sequences of the outgroup taxon *Tupaia belangeri chinensis*. In total, this represented 719 individuals: 135 great apes (Prado-Martinez et al. 2013; Xue et al. 2015; De Manuel et al. 2016; Nater et al. 2017) mapped to the human assembly hg19; 561 individuals spanning 16 primate families (including more great apes) mapped against 31 primate genomes​ as listed in the supplementary information, section S1, Supplementary Material online (Kuderna et al. 2023); 19 modern humans from the Simons Genome Diversity Project (Mallick et al. 2016) and 3 extinct hominins (Meyer et al. 2012; Prüfer et al. 2014, 2017), all which were mapped to hg19, and publicly available protein sequences of *Tupaia* as the outgroup (Fan et al. 2013). Sequences of *Nomascus leucogenys* and *Pongo tapanuliensis* were subsequently excluded due to low quality. Sequences of Neanderthal and Denisovan were only included in one case study (“Neanderthal case”).

### Amino Acid Sequence Translation and MSA

For all 14 genes under study, we restricted our analyses to the canonical isoforms from the human hg38 annotation (Ensembl) (supplementary table S1, Supplementary Material online) to ensure comparable sequences across species. The VCFs were used to integrate genomic variants in the coding sequence (CDS) of interest using samtools (Li et al. 2009) and bcftools (Li 2011) (supplementary information, section S1, Supplementary Material online). For each individual, the resulting CDS were translated to proteins through in-house python scripts based on the standard genetic code. Low-quality regions at the DNA level were represented as “N,” and affected codons masked as an “X” in the amino acid sequence.

The resulting translations were grouped by protein and aligned with MAFFT v7.520 (Katoh et al. 2002). Alignments were trimmed using trimal 1.2rev59 (Capella-Gutiérrez et al. 2009) (for parameters see supplementary information, section S1, Supplementary Material online). The resulting alignment files were manually explored and any spurious variation (in most cases due to frameshifts caused by indels) was removed or masked (for details see supplementary information, section S1, Supplementary Material online). In addition to their original annotation, the hg38 annotation was projected onto the 31 reference genomes of the 561 primates from Kuderna et al. (2023). Using the liftOver tool (Hinrichs et al. 2006) with default parameters (supplementary information, section S1, Supplementary Material online), we obtained GTF-files of the projected CDS coordinates for each of the 31 reference genomes (Kuderna et al. 2023). About half of the original annotations were previously published and have been achieved in various ways (see accessions in Kuderna et al. 2023). The other half stems from Shao et al. (2023), and has been annotated with a combination of de-novo and homology-based strategies. In some cases, the predicted protein sequence from the original annotation resulted in a higher quality protein model than LiftOver-based annotations (less premature truncation and less spurious variation), but in other cases the opposite was true. The protein model that yielded the fewest number of gaps was kept for further analysis.

Different sets of MSAs were concatenated, comprising groups of 5, 10, and 14 proteins. The 5 protein concatenation consists of AMBN, AMELX, AMTN, ENAM, and MMP20, which are proteins that are an integral part of the enamel structure and have been consistently identified from fossil teeth in previous studies (Cappellini et al. 2019; Welker et al. 2019, 2020; Madupe et al. 2023). The 10 protein concatenation represents a larger, noncollagenous enamel proteome by adding AHSG, ALB, ODAM, SERPINC1, and TUFT1. The 14 protein concatenation also included 4 collagens: COL17A1, COL1A1, COL1A2, and COL2A1. For subsequent phylogenetic analyses, the signal peptide sequence was removed from every protein sequence, given that it is usually not recovered in paleoproteomic experiments (Warinner et al. 2022). If not otherwise stated, in the following, “MSA” always refers to a concatenation of different sets of proteins of interest. Variable and parsimony-informative sites were assessed using MEGA11 (Molecular Evolutionary Genetics Analysis v. 11) (Tamura et al. 2021).

### Assessment of Protein Sequence Conservation

Shannon entropy is a measure that can be applied to MSAs to quantify the degree of variability at each given homologous site. It is agnostic to physico-chemical similarities and substitution rates between amino acids. It was calculated with a moving average of 20 (https://gist.github.com/jrjhealey/130d4efc6260dd76821edc8a41d45b6a) on the concatenated MSA of 14 proteins with 1 individual per species. Rate4Site (Pupko et al. 2002) is a tool used to calculate conservation scores in homologous amino acid sites. For the same MSA, Rate4Site scores were calculated using default options and setting the concatenated *Tupaia belangeri chinensis* proteins (outgroup) as reference sequence. Gaps in *Tupaia* proteins were filled with the consensus sequence, since the Rate4Site tool will omit sites with an incomplete reference. A moving average of 20 was used to calculate all Rate4Site scores. Alternatively, for the estimation of evolutionary rates in these proteins across mammals, Rate4Site scores were calculated on a concatenated MSA of 22 species (for list of species and sequence IDs see supplementary table S9, Supplementary Material online). The protein sequence data were downloaded from UniParc using the ProteoParc v1.0 tool (https://github.com/guillecarrillo/proteoparc). We selected a set of species that had a mostly complete sequence for each gene and that represented most clades across the group of mammals. Rate4Site scores were calculated using default parameters, setting the reference sequence to *Homo sapiens*.

### Phylogenetic Analysis

For any further downstream analysis, the MSA of all 719 individuals was downsampled to 1 individual per species (with the most complete sequence), yielding a total of 233 terminal taxa. All phylogenetic analyses were performed using ML with IQ-TREE v.1.6.12 (Nguyen et al. 2015) including the Shimodaira Hasegawa approximate likelihood-ratio test (SH-alrt), for 5,000 iterations with ultrafast bootstrap approximation. The evolutionary model of each of the individual proteins was obtained through ModelFinder (Kalyaanamoorthy et al. 2017). For a detailed description of the code and parameters see supplementary section S2 in the supplementary information, Supplementary Material online. In addition, for the complete protein sequence (except the signal peptide) of all 3 concatenations (5, 10, and 14 proteins), phylogenetic trees were also calculated using Bayesian analysis performed using MrBayes v.3.2.7a (Ronquist and Huelsenbeck 2003). Each Bayesian analysis was run for 3 million generations, with a burn-in of 25%. For all trees, the distance to the reference species tree (Kuderna et al. 2023) was assessed via RF-distance using the R package “phangorn” (Schliep 2011). Next, we calculated phylogenetic trees using the above-mentioned parameters for different subsets of amino acid positions. The rationale for building these subsets is described in the following sections.

### Ancient Sequence Reconstruction of Enamel Peptides From Fossil Specimens

Ancient peptide sequences were isolated from the tooth enamel of fossil equids and deinotheriid proboscideans of different ages. The former include specimens of *Equus* cf. *ferus* (IPS87498, 136 mg enamel powder, and IPS87522, 820 mg) from the Late Pleistocene of La Carihuela (probably <100 ka), *Equus* cf. *altidens* (IPS137786, 169 mg) from the Early Pleistocene of Vallparadís layer EVT7 (0.9 to 0.8 Ma) (Aurell-Garrido et al. 2010; Madurell-Malapeira et al. 2010), and *Hippotherium* cf. *primigenium* (IPS98842, 50 mg) from the Late Miocene of Can Llobateres 1 (9.8 Ma) (Casanovas-Vilar et al. 2016; Arranz et al. 2023). Considering that a detailed study of these fossil samples is needed, at the present time, we use the open nomenclature for these specimens. The deinotheriid specimens correspond to *Deinotherium giganteum* (IPS28029, 80 mg) from Can Llobateres (see above) and *Deinotherium levius* (IPS121827, 130 mg) from the Middle/Late Miocene of Abocador de Can Mata locality ACM/C8-A3 (11.6 Ma) (Alba et al. 2022). All the fossil specimens are housed in the Institut Català de Paleontologia Miquel Crusafont, Sabadell, Spain.

Enamel samples were precisely extracted using a rotary tool with a diamond disc and a slow-speed drill (Dremel®). Traces of dentin adhering to the enamel were removed with a scalpel and fiberglass pencil. Ancient peptide sequences were isolated from the enamel pieces in a dedicated clean room following published protocols (Cappellini et al. 2019; Welker et al. 2019), using trifluoroacetic acid as the demineralizing agent. The solubilized peptides were immobilized on a C18 membrane STAGE tip (Rappsilber et al. 2007) and washed with 5% v/v formic acid. Elution followed with a 5% v/v formic acid 50% v/v acetonitrile solution. The eluted peptides were subjected to reverse phase nanoliquid chromatography coupled with tandem mass spectrometry. Samples were analyzed using an Orbitrap Eclipse mass spectrometer (Thermo Fisher Scientific, San Jose, USA) coupled to an EASY-nLC 1200 (Thermo Fisher Scientific, San Jose, USA). More details on the run on this instrument are described in supplementary information, section S1.5, Supplementary Material online. As negative controls, extraction blanks were processed together with the ancient samples during peptide extraction. In addition, injection blanks were injected into the mass spectrometer, between the single injections of the samples and extraction blanks.

The ancient peptides were identified in iterative reference database searches using MaxQuant and MaxNovo. The databases were built from public repositories using the ProteoParc v1.0 tool. A list of the proteins in the databases and database search parameters can be found in supplementary information, section S1.6, Supplementary Material online (supplementary table S2, Supplementary Material online). The resulting ancient reconstructed sequences were used to inform the creation of subsets of the MSA.

### Reducing Alignments to Simulate Ancient Peptide Sequences

Ancient sequence reconstructions (Cappellini et al. 2018), from tooth enamel of various mammals were downloaded from publicly available data (Welker et al. 2015; Cappellini et al. 2019; Chen et al. 2019; Presslee et al. 2019; Welker et al. 2019, 2020; Madupe et al. 2023) and sequenced at the Institute for Evolutionary Biology and the Centre for Genomic Regulation in Barcelona (see section above). The ancient sequences were aligned using MAFFT v7.520 to the corresponding human reference protein from UniProt (Krueger and Fong-Zazueta 2024). The alignments were manually curated because the highly fragmentary nature of the sequenced ancient peptides can cause misalignment at nonhomologous positions. These curated alignments were then added to the predicted protein sequences of this study, using MAFFT v7.520 with the –add and –keeplength option. Inspecting the ancient sequences of different ages, we defined a set of sites which needed to be removed in order to simulate data loss due to degradation. Reducing the MSA to those positions was done using an in-house python script. The older the modeled fragmentation stage, the more sites were removed.

A list of all positional information, in relation to the single gene MSAs before concatenation, can be found in the dataset published alongside this article (Krueger and Fong-Zazueta 2024). Note that the sample ages that describe the different fragmentation stages (“100 ka,” “1 to 2 Ma,” “5 Ma,” and “10 Ma”) are based on the actual age of each sample and that most of them stem from sites with annual average temperatures higher than 10 °C. The fragmentation stage in samples of similar age might be different depending on its environment. For the stage “100 ka,” a rather large coverage of collagens is anticipated because, at this fragmentation stage, additional sampling of dentin or bone may be possible. For the stage “5 Ma,” experimental data are not available, so that this stage is an intermediate between “1 to 2 Ma” and “10 Ma.” We could not find any public peptides that stem from TUFT1, nor could we confidently sequence them. Phylogenetic analysis with ML was performed on the 4 subset MSAs, and the resulting topologies were compared against the reference tree with RF-distance.

### Case Studies of Simulated Ancient Samples

With the aim of simulating typical phylogenetic inference with paleoproteomic data, several phylogenetic analyses were performed as case studies (“Neanderthal case,” “Chimpanzee case,” “Colobine case,” and “Lemur case”). In these 4 scenarios, the “100 ka” fragmentation pattern was used for the “Neanderthal case” and the “Lemur case,” and the “5 Ma” pattern for the “Chimpanzee case” and the “Colobine case,” aiming to mimic the fragmentary pattern that could be recovered from actual fossils after their split from their most recent extant sister group. The objective of these 2 analyses was to observe if the fragmented data still allowed the individuals to be positioned correctly in the phylogenetic tree. The reference data for the “Neanderthal case” consisted of a concatenation of the set of 14 proteins (full-length) of 5 individuals from each of the hominid species, including 5 *Homo sapiens* individuals, and 1 *Hylobates lar* individual as an outgroup. The 3 Neanderthal/Denisovan sequences with the “100 ka” fragmentation pattern were added to this scaffold. The “Chimpanzee case” also comprised 14 concatenated proteins of 5 individuals per hominid species, including 5 *Homo sapiens*, but excluding *P. troglodytes*, and using *Hylobates lar* as an outgroup. Three simulated *P. troglodytes* sequences with the fragmentation pattern of “5 Ma” were then added to this scaffold. The reference data for the “Colobine case” consisted of 1 individual of all available species of the subfamily Colobinae. Fragmented sequence data (stage “5 Ma”) of 3 individuals from different colobine species was added to this reference. The reference data for the “Lemur case” consisted of 1 individual for all available species in Lemuroidea. Fragmented sequence data (stage “100 ka”) of 3 individuals from different lemur species was added to this reference. ML phylogenetic analysis was performed on all of these case studies. For the “Colobine case”, we also tested approaches with mixture models and fixed topologies. For more details, see the supplementary information, section S5, Supplementary Material online.

### Phylogenies by Amino Acid Conservation

For subsequent analysis, the MSAs were separated into sections of higher or lower conservation. We used 2 methods (Rate4Site and Shannon entropy) to measure variability of each site in the MSA. The Rate4Site score and Shannon entropy values were calculated for each protein and normalized to a mean of 1. The MSAs of each protein were then subset by values equal or higher than 1 and lower than 1 and concatenated into a long MSA. For the 2 metrics, this resulted in 4 different types of concatenated MSAs “Shannon variable,” “Shannon conserved,” “Rate4Site variable,” and “Rate4Site conserved.” This, applied to all 3 concatenations (5, 10, and 14 proteins), resulted in a total of 12 MSAs. Phylogenetic analysis with ML was performed on each of them. The resulting tree topologies were compared to the trees resulting from the full-length proteins and to the reference tree using RF-distance.

### Evolutionary Rate Covariation Scores

The degree of evolutionary covariation of the set of 14 genes was estimated using Evolutionary Rate Covariation (ERC) analysis (Clark et al. 2012). The ERC for 19,137 orthologous genes from 120 mammalian species was calculated using the R code available at https://github.com/nclark-lab/erc. The covariation in relative evolutionary rates for each gene pair was calculated using only the branches that are shared between the 2 genes. The raw correlations were then Fisher-transformed, normalizing for the number of branches that contributed to the correlation. In R v.4.3.1, significance was estimated via permutation analysis using the mean as test statistic and 10,000 permutations. The results were plotted in R using the package “ggplot2” (Wickham 2016).

## Supplementary Material

evaf007_Supplementary_Data

## Data Availability

The mass spectrometry proteomics data have been deposited to the ProteomeXchange Consortium (Deutsch et al. 2017) via the PRIDE (Perez-Riverol et al. 2022) partner repository with the dataset identifier PXD048972. Additional supplementary data, Supplementary Material online to the supplementary files associated with this publication, is deposited at Zenodo under the doi 10.5281/zenodo.10637110. All code is available under github.com/RicardoFong/primate_enamelome.
