# Supplementary material for: Phylogenetic Signal in Primate Tooth Enamel Proteins and its Relevance for Paleoproteomics
Source: Genome Biol Evol. 2025 Jan 21;17(2):evaf007. doi: 10.1093/gbe/evaf007 (PMC11878541; doi:10.1093/gbe/evaf007)
Supplement: evaf007_Supplementary_Data [file evaf007_supplementary_data.zip › Supplementary_information.pdf]

Supplementary information

Phylogenetic signal in primate tooth enamel proteins and its relevance for paleoproteomics

S1. Dataset generation

All mentioned code that is not explicitly explained here, is available under github.com/RicardoFong/primate_enamelome.

S1.1 Prediction of protein sequences

The dataset consisted of 722 individuals of 233 species, spanning the 16 families of the order Primates. Identifiers and species are available at Zenodo under the doi 10.5281/zenodo.10637110.

Not every species has a reference assembly, and so the individuals of some species were mapped to one of their closest relatives ("reference species"). If not specified otherwise, the assembly for a species is the same as in Kuderna et al. (2023). The mapping went as follows:

Reference species: *Aotus nancymaae*

Species mapped:

Aotus azarae

Aotus griseimembra

Aotus trivirgatus

Aotus vociferans

Reference species: *Ateles fusciceps*

Species mapped:

Alouatta belzebul

Alouatta caraya

Alouatta discolor

Alouatta juara

Alouatta macconnelli

Alouatta nigerrima

Alouatta palliata

Alouatta puruensis

Alouatta seniculus

Ateles belzebuth

Ateles chamek

Ateles geoffroyi

Ateles marginatus

Ateles paniscus

Lagothrix lagothricha

Reference species: *Callithrix jacchus*

Species mapped:

Callibella humilis

Callimico goeldii

Callithrix geoffroyi

Callithrix jacchus

Callithrix kuhlii

Cebuella niveiventris

Cebuella pygmaea

Leontopithecus chrysomelas

Leontopithecus rosalia

Mico argentatus

Mico humeralifer

Mico spnv

Reference species: *Cebus albifrons*

Species mapped:

Cebus albifrons

Cebus olivaceus

Cebus unicolor

Saimiri cassiquiarensis

Saimiri macrodon

Saimiri oerstedii

Saimiri sciureus

Saimiri ustus

Sapajus macrocephalus

Reference species: Cercocebus atys

Species mapped:

Cercocebus chrysogaster

Cercocebus lunulatus

Cercocebus torquatus

Reference species: *Cercopithecus mitis*

Species mapped:

Cercopithecus ascanius

Cercopithecus cephus

Cercopithecus diana

Cercopithecus hamlyni

Cercopithecus lowei

Cercopithecus mitis

Cercopithecus mona

Cercopithecus neglectus

Cercopithecus nictitans

Cercopithecus petaurista

Cercopithecus pogonias

Cercopithecus roloway

Miopithecus ogouensis

Reference species: *Chlorocebus aethiops*

Species mapped:

Allenopithecus nigroviridis

Allochrocebus lhoesti

Allochrocebus preussi

Allochrocebus solatus

Chlorocebus pygerythrus

Reference species: *Colobus guereza*

Species mapped:

Colobus angolensis

Colobus guereza

Colobus polykomos

Reference species: *Daubentonia madagascariensis*

Species mapped:

Daubentonia madagascariensis

Reference species: *Erythrocebus patas*

Species mapped:

Erythrocebus patas

Reference species: *Galago moholi*

Species mapped:

Galagoides demidoff

Galago moholi

Galago senegalensis

Reference species: *Gorilla gorilla gorilla*

Species mapped:

Gorilla beringei

Gorilla gorilla

Reference species: *Homo sapiens* (hg19)

Species mapped:

Homo sapiens

Neanderthal

Denisovan

Gorilla beringei (partly, see methods)

Gorilla gorilla (partly, see methods)

Pan troglodytes (partly, see methods)

Pan paniscus (partly, see methods)

Pongo abelii (partly, see methods)

Pongo pygmaeus (partly, see methods)

Reference species: *Lemur catta*

Species mapped:

Eulemur albifrons

Eulemur collaris

Eulemur coronatus

Eulemur flavifrons

Eulemur fulvus

Eulemur macaco

Eulemur mongoz

Eulemur rubriventer

Eulemur rufus

Eulemur sanfordi

Hapalemur alaotrensis

Hapalemur gilberti

Hapalemur griseus

Hapalemur meridionalis

Hapalemur occidentalis

Lemur catta

Prolemur simus

Varecia rubra

Varecia variegata

Reference species: *Loris tardigradus*

Species mapped:

Loris lydekkerianus

Loris tardigradus

Reference species: *Macaca mulatta*

Species mapped:

Macaca arctoides

Macaca assamensis

Macaca cyclopis

Macaca fascicularis

Macaca fuscata

Macaca leonina

Macaca maura

Macaca mulatta

Macaca nemestrina

Macaca nigra

Macaca radiata

Macaca siberu

Macaca silenus

Macaca thibetana

Macaca tonkeana

Reference species: *Mandrillus sphinx*

Species mapped:

Lophocebus aterrimus

Mandrillus leucophaeus

Mandrillus sphinx

Reference species: *Microcebus murinus*

Species mapped:

Cheirogaleus major

Cheirogaleus medius

Lepilemur ankaranensis

Lepilemur dorsalis

Lepilemur ruficaudatus

Lepilemur septentrionalis

Microcebus murinus

Mirza zaza

Reference species: *Nomascus leucogenys*

Species mapped:

Hoolock hoolock

Hylobates abbotti

Hylobates agilis

Hylobates klossii

Hylobates lar

Hylobates muelleri

Nomascus annamensis

Nomascus concolor

Nomascus gabriellae

Nomascus leucogenys

Nomascus siki

Reference species: *Nycticebus pygmaeus*

Species mapped:

Arctocebus calabarensis

Nycticebus bengalensis

Nycticebus coucang

Nycticebus pygmaeus

Perodicticus ibeanus

Perodicticus potto

Reference species: *Otolemur garnettii*

Species mapped:

Otolemur crassicaudatus

Otolemur garnettii

Reference species: *Pan troglodytes*

Species mapped:

Pan troglodytes

Reference species: *Papio anubis*

Species mapped:

Papio anubis

Papio cynocephalus

Reference species: *Pithecia pithecia*

Species mapped:

Cacajao ayresi

Cacajao calvus

Cacajao hosomi

Cacajao melanocephalus

Cheracebus lucifer

Cheracebus lugens

Cheracebus regulus

Cheracebus torquatus

Chiropotes albinasus

Chiropotes israelita

Chiropotes sagulatus

Pithecia albicans

Pithecia chrysocephala

Pithecia hirsuta

Pithecia mittermeieri

Pithecia pissinattii

Pithecia vanzolinii

Plecturocebus bernhardi

Plecturocebus brunneus

Plecturocebus caligatus

Plecturocebus cinerascens

Plecturocebus cupreus

Plecturocebus dubius

Plecturocebus grovesi

Plecturocebus hoffmannsi

Plecturocebus miltoni

Plecturocebus moloch

Reference species: *Pongo abelii*

Species mapped:

Pongo abelii

Pongo pygmaeus

Reference species: *Propithecus coquereli*

Species mapped:

Avahi laniger

Avahi peyrierasi

Indri indri

Propithecus coquereli

Propithecus coronatus

Propithecus diadema

Propithecus edwardsi

Propithecus perrieri

Propithecus tattersalli

Propithecus verreauxi

Reference species: *Macaca mulatta*

Species mapped:

Papio anubis

Papio cynocephalus

Papio hamadryas

Papio kindae

Papio papio

Papio ursinus

Reference species: *Rhinopithecus roxellana*

Species mapped:

Nasalis larvatus

Piliocolobus badius

Piliocolobus gordonorum

Piliocolobus kirkii

Piliocolobus tephrosceles

Presbytis comata

Presbytis mitrata

Pygathrix cinerea

Pygathrix nemaeus

Pygathrix nigripes

Rhinopithecus bieti

Rhinopithecus roxellana

Semnopithecus entellus

Semnopithecus hypoleucos

Semnopithecus johnii

Semnopithecus priam

Semnopithecus schistaceus

Semnopithecus vetulus

Trachypithecus auratus

Trachypithecus crepusculus

Trachypithecus cristatus

Trachypithecus francoisi

Trachypithecus geei

Trachypithecus germaini

Trachypithecus hatinhensis

Trachypithecus laotum

Trachypithecus leucocephalus

Trachypithecus melamera

Trachypithecus obscurus

Trachypithecus phayrei

Trachypithecus pileatus

Reference species: *Saguinus midas*

Species mapped:

Leontocebus fuscicollis

Leontocebus illigeri

Leontocebus nigricollis

Saguinus bicolor

Saguinus geoffroyi

Saguinus imperator

Saguinus inustus

Saguinus labiatus

Saguinus midas

Saguinus mystax

Saguinus oedipus

Reference species: *Sapajus apella*

Species mapped:

Sapajus apella

Sapajus macrocephalus

Reference species: *Tarsius syrichta*

Species mapped:

Carlito syrichta

Cephalopachus bancanus

Tarsius lariang

Tarsius wallacei

Reference species: *Theropithecus gelada*

Species mapped:

*Theropithecus gelad*a

Projection of the human hg38 annotation to each assembly was performed via LiftOver to obtain GTF files with the coordinates of the coding sequences (CDSs) of each of the genes. The Ensembl ID of the hg38 canonical forms used are in supplementary table S1. The coordinates for each of the references used, and the liftedOver beds are available at Zenodo under the doi 10.5281/zenodo.10637110. For the 157 representatives of the family Hominidae (i.e., great apes, and fossil and modern humans) that were originally mapped to hg19, the hg19 human annotation was used for simplicity, because the annotations of the genes of interest did not show any differences between the hg19 and hg38 annotations.

Supplementary table S1: Ensembl identifiers of the human canonical isoforms used for this study

**Gene**	**ENSEMBL ID**
AHSG	ENST00000411641.7
ALB	ENST00000295897.9
AMBN	ENST00000322937.10
AMELX	ENST00000380714.7
AMTN	ENST00000339336.9
COL17A1	ENST00000648076.2
COL1A1	ENST00000225964.10

COL1A2	ENST00000297268.11
COL2A1	ENST00000380518.8
ENAM	ENST00000396073.4
MMP20	ENST00000260228.3
ODAM	ENST00000683306.1
SERPINC1	ENST00000367698.4
TUFT1	ENST00000368849.8

The VCFs were queried, and the sequences obtained filtered through an in-house script using samtools (Li et al. 2009) and bcftools (Li 2011), and then translated using python with standard genetic code.

The genotype calling was performed through the following expression:

samtools faidx assembly.fasta ${scaffold}:${start}-${stop} | bcftools consensus –sample ${samplename} -m mask.bed -H 1 file.vcf

SNPs were called and filtered using the expression:

TYPE!='snp' | (GT='het' & FMT/AD[*:*] < 3) | AC > 2 | FMT/DP <= 10 | QD < 2 | FS > 60 | INFO/MQ < 40 | SOR > 3 | ReadPosRankSum < -8 | QUAL < 30 | MQRankSum < -12.5

And INDELS were called and filtered using:

TYPE!='indel' | (GT='het' & FMT/AD[*:*] < 3) | FMT/DP <= 10 | QD < 2 | FS > 200 | INFO/MQ < 40 | SOR > 3 | ReadPosRankSum < -20 | QUAL < 30

At heterozygous positions, bcftools was randomly run either with the option –H1 or –H2 for random allele selection.

S1.2 Selecting the more comparable sequences for analysis

For the 561 individuals stemming from Kuderna et al. (2023) two sets of translations were performed, one using the original annotation files present in its publication, and the other by using the liftOver coordinates from hg38 reference genome for each of the 32 genomes used (previous section). For both translations, each of the sequences were aligned against the human hg38 canonical form with MAFFT v7.490 (Katoh & Standley, 2013). If only an original, or a liftOver-based translated sequence was available, this sequence was kept for further analysis. If both versions of the sequence were present, gaps of each sequence aligned to the hg38 reference were counted. The sequence that had less gaps when aligned against the hg38 model was kept for further analysis. The resulting set of sequences is a mix of original and liftOver sequences. A file describing which model was kept for each of the genes per reference is available at Zenodo (10.5281/zenodo.10637110).

S1.3 Aligning and refining the sequences

Proteins were aligned using MAFFT v7.490 through the command:

```
mafft --maxiterate 1000 --globalpair protein.fasta > aligned_protein.fasta
```

After alignment, a first round of less stringent trimming was performed using the program trimAl 1.2rev59 (Capella-Gutiérrez et al. 2009) with the command:

```
trimal -in <inputfile> -out <outputfile> -gt 04 -cons 09
```

For some proteins the value of -gt was lowered from 0.9 to 0.4 and the option -cons was dropped (minimum percentage of the positions in the original alignment to conserve), resulting in proteins COL17A1 and COL1A1 trimmed with -gt= 0.4, COL2A1 -gt=0.8, ENAM and TUFT1 -gt= 0.9.

After trimming, the proteins were explored through jalview for nonsense variation caused by frameshifts, most likely due to indels. The coordinates of the nonsense variation were manually registered into tsv files available at Zenodo (10.5281/zenodo.10637110). These coordinates were used to find the positions in the DNA sequence causing the frame shift, and edit the DNA in order to recover the frame through an in-house python script.

After recovering the frame of the proteins they were again aligned through the command,

```
mafft --maxiterate 1000 --globalpair protein.fasta > aligned_protein.fasta
```

and trimmed with trimal.

AHSG, ALB, AMBN AMELX, AMTN MMP20, ODAM and SERPINC1 were trimmed with the parameters -gt 09 –cons 60; COL17A1 and COL1A1 with -gt 04; ENAM and TUFT1 with -gt 09.

The resulting trimmed files were further revised through jalview. Any remaining spurious variation was masked with a “?”.

S1.4 Testing the effect of intraspecific variation

In order not to skew the analysis results toward species that are overrepresented with many individuals, most analyses were performed on a multiple sequence alignment (MSA) that contained exactly one individual per species. For each species in this MSA, the individual with the most complete sequences (i.e. fewest “?”, “X” or “-” characters) was chosen. To test the influence on the outcome, if other individuals had been chosen, we created 1,000 MSAs by randomly sampling one individual per species. Hence, each of these MSAs comprises the concatenated proteins from 232 primate individuals and one individual of the outgroup *Tupaia belangeri chinensis*. Shannon entropy, Rate4Site score, and a maximum likelihood ML tree (IQ-TREE v. 1.6.12) were calculated for each of the 1,000 MSAs (supplementary figure S1). All trees were compared to a reference species tree that is based on high quality genomic data (Kuderna et al. 2023) by measuring the Robinson-Foulds distances (RF-distances) between them. All RF-distances to the reference tree range between 82-156,

showing median (110) and mean (112.7) values that are very close to the RF-distance between the phylogeny based on a single representative individual per species (the one with most complete sequence) and the reference tree (108). The mean differences of Shannon entropy at all given sites range from 0.77% to 2.81% of the possible range of values, with a mean difference of 1.71% (mean difference in entropy value 0.05). The mean differences of Rate4Site scores at all given sites range from 0.50% to 1.36% of the possible range of values, with a mean difference of 0.92% (mean difference in Rate4Site score 0.04). From these results, we assumed that the concatenated MSA of one individual per species with the most complete sequence is representative and continued all downstream analyses with this set of individuals (supplementary file 2).

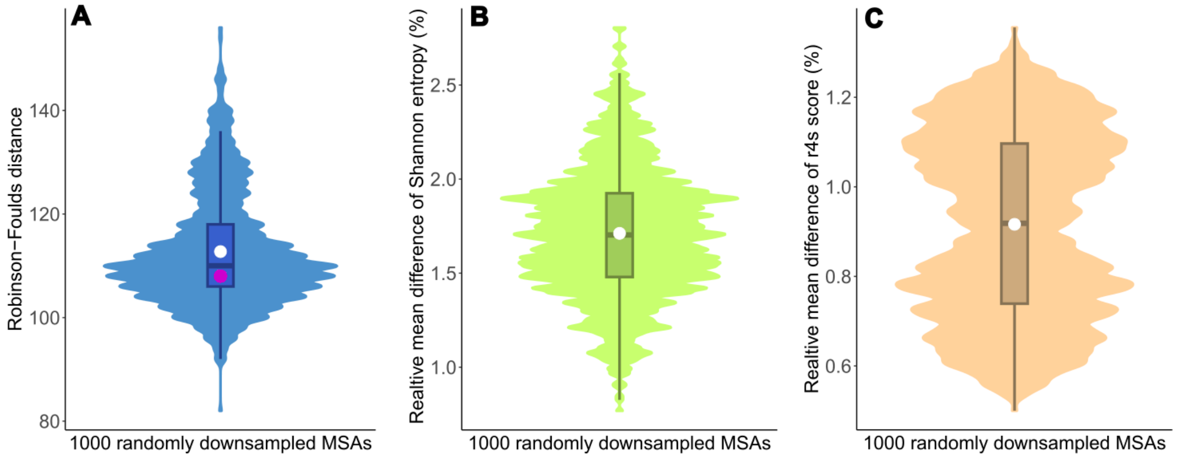

Supplementary figure S1: Relative Shannon entropy, Rate4Site scores, and Robinson-Foulds distances of 1000 randomly downsampled MSAs
A - Robinson-Foulds distances (RF-distances) to reference species tree (Kuderna et al. 2023), mean is marked as white dot, RF-distance of most complete MSA is marked as pink dot, B - average difference between Shannon entropy at each site of random MSA and most complete MSA as percentage of range of values, C - average difference between Rate4Site score at each site of random MSA and most complete MSA as percentage of range of values

S1.5 Reversed phase liquid chromatography tandem mass spectrometry

In order to simulate sequence degradation, we needed to create a simple model of how tooth enamel protein sequences degrade *post mortem*. We mainly used publicly available data (more details see methods in main text), but also added some ancient samples whose processing is described in the following sections.

Samples were analyzed using an Orbitrap Eclipse mass spectrometer (Thermo Fisher Scientific, San Jose, USA) coupled to an EASY-nLC 1200 (Thermo Fisher Scientific, San Jose, USA).

Peptides were loaded directly onto the analytical column and were separated by reversed-phase chromatography using a 50-cm column with an inner diameter of 75 μm, packed with 2 μm C18 particles spectrometer (Thermo Scientific, San Jose, USA).

Chromatographic gradients started at 95% buffer A and 5% buffer B with a flow rate of 300 nl/min and gradually increased to 25% buffer B and 75% A in 105 min and then to 40% buffer B and 60% A in 15 min. After each analysis, the column was washed for 10 min with 100% buffer B. Buffer A: 0.1% formic acid in water. Buffer B: 0.1% formic acid in 80% acetonitrile.

The mass spectrometer was operated in positive ionization mode with nanospray voltage set at 2.4 kV and source temperature at 305 °C. The acquisition was performed in data-dependent acquisition (DDA) mode and full MS scans with 1 micro scan at resolution of 120000 were used over a mass range of m/z 350-1400 with detection in the Orbitrap mass analyzer. Auto gain control (AGC) was set to 4E6 and injection time to "auto". In each cycle of data-dependent acquisition analysis, following each survey scan, the most intense ions above a threshold ion count of 10000 were selected for fragmentation. The number of selected precursor ions for fragmentation was determined by the "Top Speed" acquisition algorithm and a dynamic exclusion of 60 seconds. Fragment ion spectra were produced via high-energy collision dissociation (HCD) at normalized collision energy of 28% and they were acquired in the Orbitrap mass analyzer at resolution 30000. AGC was set to 3E4, and an isolation window of 0.7 m/z and a maximum injection time of 54 ms were used.

Four blank samples were injected before and after each sample to avoid sample carryover. Digested bovine serum albumin (New england biolabs cat # P8108S) was analyzed between each sample to avoid sample carryover and to assure stability of the instrument and QCloud (Chiva et al. 2018) has been used to control instrument longitudinal performance during the project.

S1.6 Ancient protein sequence reconstruction

Equid samples were run using MaxQuant v.1.6.17.0, with the following settings different from default as described in supplementary table S2. The reference equid enamelome database was mainly built from publicly available protein sequences of ALB, COL1A2, COL1A1, COL2A1, COL17A1, AMBN, AMELX, ENAM, AMELY, AMTN, MMP20, KLK4, TUFT1, SERPINC1 and ODAM from UniProt and GenBank. All isoforms were included.

Deinotheriid samples were run using MaxQuant v2.0.2.0, including MaxNovo, with the following settings different from default as described in supplementary table S2.

The reference Proboscidea enamelome database was built using ProteoParc v1.0. The TaxID was set as 9779 (Order Proboscidea) and the search was focused on the above described teeth proteins: ALB, COL1A2, COL1A1, COL2A1, COL17A1, AMBN, AMELX, ENAM, AMELY, AMTN, MMP20, KLK4, TUFT1, SERPINC1 and ODAM.

Ancient sequence reconstruction was performed grouping the peptide sequences by the most common "Leading razor protein" in each protein group in the MaxQuant output. Reverse and contaminant peptides were removed from the final reconstruction during this step.

Supplementary table S2: MaxQuant parameter settings
Carbamidomethyl (C) was removed from the fixed modifications parameter as the sample preparation does not produce this type of chemical conversion.

Parameter	Setting v.1.6.17.0	Setting v.2.0.2.0
Digestion	Unspecific	Unspecific
Fixed modifications	None	None
Variable modifications	Oxidation of M; Oxidation of P; Gln->Pyro-Glu; Glu->Pyro-Glu; Deamidation (NQ); Phosphorylation (ST)	Oxidation of M; Oxidation of P; Gln->Pyro-Glu; Glu->Pyro-Glu; Deamidation (NQ); Phosphorylation (ST)
Maximum number of modifications per peptide	5	4
Main search peptide tolerance	4.5 ppm	10 ppm
Individual peptide mass tolerance	TRUE	FALSE
Minimum peptide length for unspecific search	7	7
Protein FDR	1	1
Dependent peptides	FALSE	TRUE
De-novo sequencing	FALSE	TRUE
Minimum delta score for modified peptides	0	0
Minimum score for unmodified peptides	40	40
use .NET Core	TRUE	FALSE (for windows OS)

S2. Concatenations and Phylogenetic analysis

S2.1 Concatenation and trimming

The signal peptide was removed, by trimming the following number of amino acids from the beginning of the sequence of each protein, following the signal peptide sequence reported at UniProt:

AHSG	17
ALB	17
AMBN	21
AMTN	16
COL17A1	0
COL1A1	22
COL1A2	22
COL2A1	25
ENAM	39

ODAM	15
MMP20	22
SERPINC1	32
TUFT1	20

The trimmed sequences were concatenated into three different groups of 5, 10 and 14 proteins with the command:

```
$ catfasta2phyml.pl prot1.fasta prot2.fasta (...) protX.fasta > concatenation.fasta
```

The three concatenations that were performed are:

5 proteins: AMBN, AMELX, AMTN, ENAM, MMP20

10 proteins: AHSG, ALB, AMBN, AMELX, AMTN, ENAM, MMP20, ENAM, ODAM, SERPINC1

14 proteins: AHSG, ALB, AMBN, AMELX, AMTN, COL17A1, COL1A1, COL1A2, COL2A1, ENAM, MMP20, ODAM, SERPINC1

The number of amino acid sites (i.e. length) of each MSA is displayed in supplementary table S3.

Supplementary table S3: Details of multiple sequence alignments

Number of proteins in concate-nation	Proteins	Length MSA full sequence (w/o signal) / variable sites / parsimony inform. sites (aa)	Length MSA fragmented "100 ka"	Length MSA fragmented "1-2 Ma"	Length MSA fragmented "5 Ma"	Length MSA fragmented "10 Ma"	Length of each "variable" / "conserved " MSA
5	AMBN, AMELX, AMTN, ENAM, MMP20	2339 / 1443 / 1225	NA	NA	NA	NA	shannon: 826 / 1513 r4s: 878 / 1461
10	AMBN, AMELX, AMTN, ENAM, MMP20, AHSG, ALB, ODAM, SERPINC1, TUFT1	4327 / 2547 / 2118	NA	NA	NA	NA	shannon: 1535 / 2792 r4s: 1557 / 2770
14	AMBN, AMELX, AMTN, ENAM, MMP20, AHSG, ALB, ODAM, SERPINC1, TUFT1,	9557 / 3636 / 3050	3884	1139	593	98	shannon: 3632 / 5925 r4s: 2516 / 7041

	COL1A1, COL1A2, COL2A1, COL17A1						

S2.2 Phylogenetic analysis on full length sequences

ML and Bayesian phylogenetic analyses were performed for the three concatenations.

For the phylogenetic analysis, the dataset was reduced to one individual per species, yielding a dataset of 233 individuals.

The evolutionary model of each of the proteins was obtained using modelfinder (Kalyaanamoorthy et al. 2017) with the command:

iqtree -s Aligned_protein_file.fasta -nt 6 -redo -m MF -msub nuclear

yielding the following models:

AHSG = JTT+R3
ALB = JTTDCMut+G4
AMBN = JTT+I+G4
AMELX = JTTDCMut+F+G4
AMTN = JTT+R2
COL17A1 = JTT+R3
COL1A1 = JTTDCMut+F+R3
COL1A2 = JTTDCMut+F+R3
COL2A1 = JTTDCMut+F+R3
ENAM = JTT+R3
MMP20 = JTT+I+G4
ODAM = JTT+I+G4
SERPINC1 = JTT+I+G4
TUFT1 = JTT+G4

ML phylogenetic analysis was performed with IQ-TREE1.6.12 (Nguyen et al. 2015) through the command:

iqtree -nt 4 -s $infile -spp $partfile -alrt 5000 -bb 5000 -nstop 500 -nm 10000 -wsplits -pre $outdir

Bayesian analysis was performed with MrBayes v.3.2.7a (Ronquist & Huelsenbeck 2003) for 3 million generations with the parameters:

rates=invgamma nucmodel=Protein;
unlink statefreq=(all) revmat=(all) shape=(all) pinvar=(all);
prset applyto=(all) ratepr=variable aamodel=mixed;

```
temp=0.2
Ngen=3000000
Diagnfreq=5000
Nruns=2
Nchains=4
relburnin=yes
Burninfrac=0.25
samplefreq=500
printfreq=500
starttree=random
savebrlens=yes
startparams=reset xx  x
Filename=out_files/14_proteins/14_proteins.out;
```

Convergence was assessed with TRACER (Rambaut et al. 2018) (output available at 10.5281/zenodo.10637110)

The ML trees based on the 5 and 10 protein concatenations are displayed in supplementary figure S2, the 14 protein ML tree is displayed in Fig.4 in the main text. The RF-distance between all full sequence protein trees and the reference tree is displayed in supplementary figure S3.

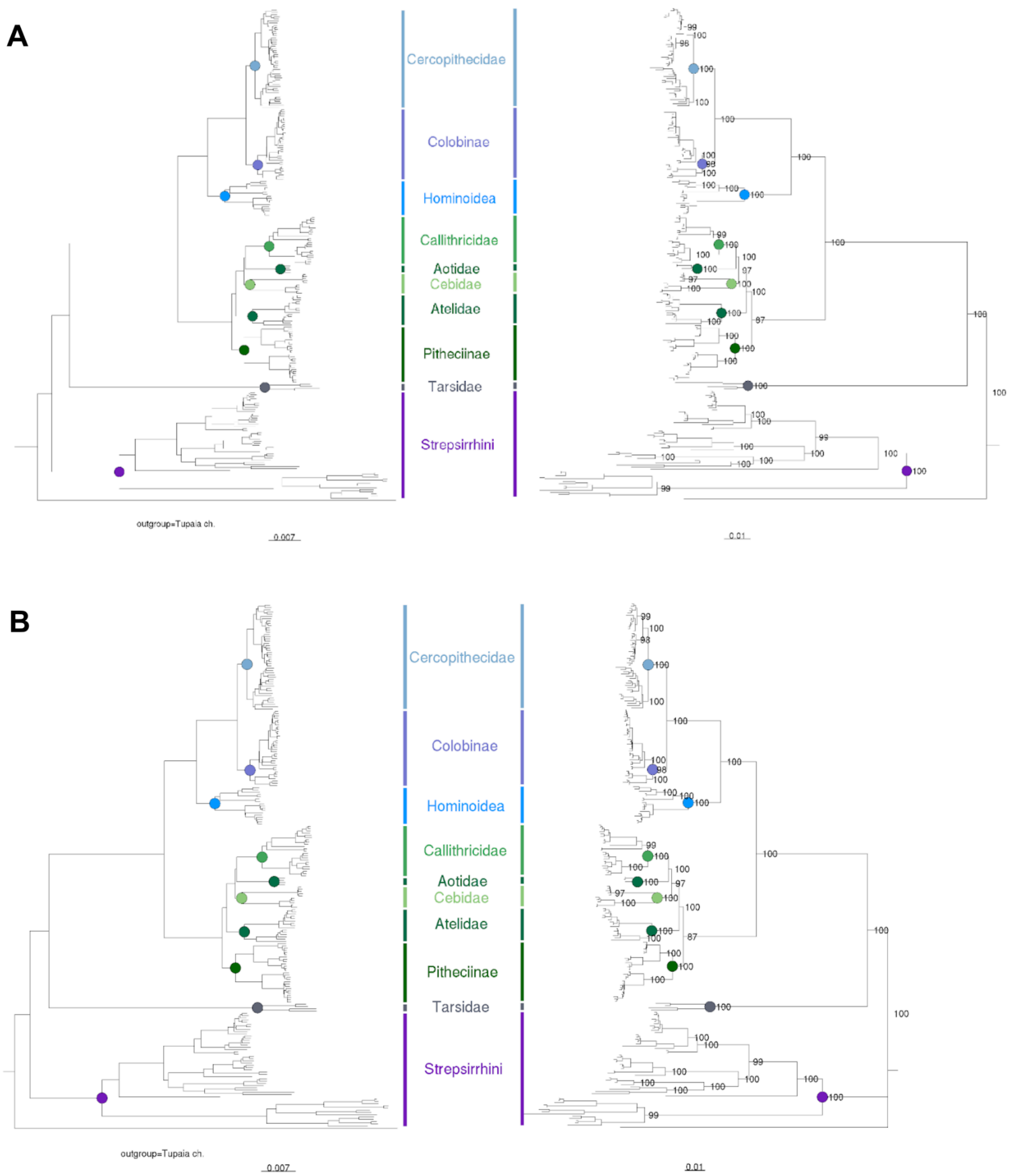

Supplementary figure S2: Phylogenies based on concatenation of five and ten proteins
Created using ML (IQ-TREE v1.6.12). A - five proteins, B - ten proteins. Trees on the left are the reference tree, trees on the right the phylogenies computed with the concatenations.

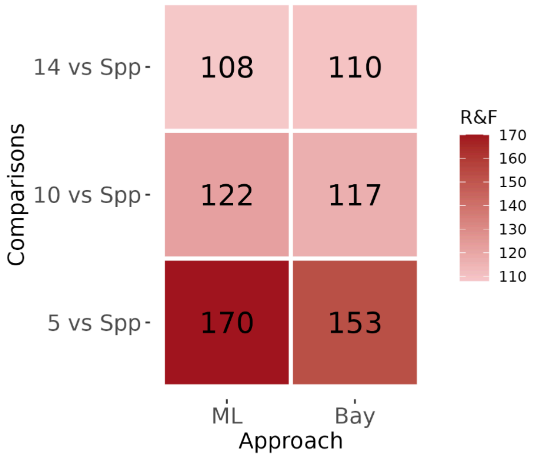

Supplementary figure S3: Robinson-Foulds distances between phylogenies based on 5, 10, and 14 protein concatenation and reference species tree for maximum likelihood and Bayesian approach
Spp - Reference species tree based on whole genome data (Kuderna et al. 2023), ML - maximum likelihood approach (IQ-TREE v. 1.6.12), Bay - Bayesian approach (MrBayes v.3.2.7a).

The computational times with similar resources were substantially shorter using IQ-TREE. They are shown in supplementary table S4.

Supplementary table S4: Computational times for Bayesian and Maximum Likelihood approach
Format in days-hours:minutes:seconds.

	14 proteins	10 proteins	5 proteins
Bayesian (MrBayes)	12-10:05:57	6-16:13:46	4-07:32:22
Maximum Likelihood (IQ-TREE)	03:46:34	01:32:54	02:10:46

S2.3 Phylogenetic analysis on different combinations of collagens

In the 14 protein concatenation-based phylogenies, which contain collagens, it was seen that the tarsiers appeared wrongly positioned as monophyletic with strepsirrhines (see Results in main text). For this reason, we performed phylogenetic analysis on each of the collagens, and in all the permutations possible of collagens along with the complete set of non-collagen proteins (10 proteins). This approach allowed us to explore which of the proteins were driving the observed placement of the tarsiers in the trees based on the 14 protein concatenations. We can see in supplementary table S5 in which sequences or combinations of sequences the tarsiers appear correctly (i.e. “sister to Simiiformes”) or incorrectly positioned.

Supplementary table S5: Phylogenetic position of tarsiers when computing ML with the selection of protein sequences displayed in column 1.
“E” stands for enamel and represents 10 non-collagenous enamel proteins (AHSG, ALB, AMBN, AMELX, AMTN ENAM, MMP20, ODAM, SERPINC1, TUFT1).

Enamel and Collagens in dataset	Position of Tarsiers	Bootstrap value
COL17A1	sister to strepsirrhines	55
COL1A1	Tarsiidae clusters with Lorisidae and Galagidae. This cluster is a sister group to the rest of strepsirrhines.	92
COL1A2	sister to strepsirrhines	98
COL2A1	sister to simiiformes, with strepsirrhines unresolved	100
4 collagens	sister to strepsirrhines	99
Enamel (E) + 4 collagens	sister to strepsirrhines	90
E + COL17A1, COL1A2	sister to strepsirrhines	73
E + COL1A1, COL1A2	sister to strepsirrhines	78
E + COL17A1, COL1A1, COL1A2	sister to strepsirrhines	85
E + COL1A1, COL2A1, COL1A2	sister to strepsirrhines	78
E + COL17A1, COL1A2, COL2A1	sister to strepsirrhines	72
E + COL17A1, COL1A1	sister to simiiformes	100
E + COL17A1, COL2A1	sister to simiiformes	95
E + COL1A1, COL2A1	sister to simiiformes	95
E + COL1A2, COL2A1	sister to simiiformes	93
E + COL17A1, COL1A1, COL2A1	sister to simiiformes	88
E + COL17A1	sister to simiiformes	95
E + COL1A1	sister to simiiformes	98
E + COL1A2	sister to simiiformes	93
E + COL2A1	sister to simiiformes	100

S2.4 Alternative phylogenetic methods for further validation

To further validate the results of the 14 protein concatenation that placed tarsiers with strepsirrhines, we tested alternative approaches that applied mixture models. We assessed the fit of the models to examine the likelihood of model misspecification.

As Maximum Likelihood approach we used IQ-TREE2 (Minh et al. 2020):

```
iqtree2 -s protein_aligned.fas -m MFP -mset LG+F+G,WAG+F+G,JTT+F+G,GTR20 -madd
LG+C20+F+G,LG+C10+F+G,LG+C30+F+G,LG+C40+F+G,LG+C50+F+G,LG+C60+F+G,C1
0,C20,C30,C40,C50,C60,EX2,EX3,EHO,LG4M,LG4X -nt 8 -bb 1000
```

This command was applied to each of the individual protein alignments described in section S1.3 of this document, yielding the following models:

AHSG=JTT+F+R3
ALB=LG+C20+F+G
AMBN=JTT+F+G4
AMELX=JTT+F+G4
AMTN=JTT+F+R2
COL17A1=JTT+F+R3

COL1A1=JTT+F+I+G4
COL1A2=JTT+F+R3
COL2A1=JTT+F+R2
ENAM=GTR20+F+I+G4
MMP20=JTT+F+I+G4
ODAM=JTT+F+I+G4
SERPINC1=C60
TUFT1=JTT+F+G4

We then calculated maximum likelihood phylogenies for the three protein concatenations (5, 10 and 14 proteins described in section S2.1) using these new models, and applying the IQ-TREE command described in section S2.2 of this document.

The resulting phylogeny placed tarsiers as a monophyletic group with strepsirrhines, consistent with all previously described approaches (supplementary figure S4).

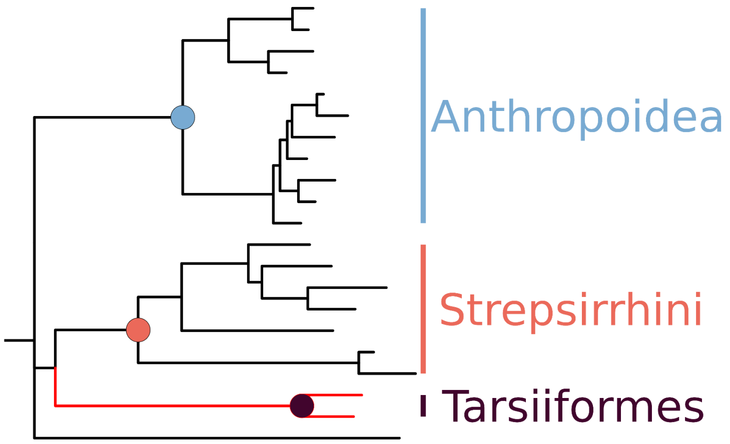

Supplementary figure S4: Placement of tarsiers when using mixture models in IQ-TREE2. The node that splits Strepsirrhini from Tarsiiformes has a bootstrap support of 88. *Tupaia belangeri chinensis* forms the branch at the bottom. It forms the outgroup to the primates of this tree (short branch length). The tree shown stems from the 14 proteins concatenation.

IQTREE2 offers several statistics to see whether the data fits some of the assumptions made by models (Naser-Khdour et al. 2019). It can test for the assumptions that amino-acid frequencies remain constant over time (stationarity) and that substitutions are equally likely in both directions (reversibility). It was performed as follows:

```
iqtree2 -s example.phy -p example.nex --symtest-only
```

The results do not indicate any violations of these two assumptions by any collagen as is evident in the SymPval column of supplementary table S6, where a value < 0.05 indicates that the assumptions of stationarity or homogeneity, or both are rejected (Naser-Khdour et al. 2019). Only for ODAM could significant deviations from the assumption of stationarity be observed; however the inclusion or exclusion of ODAM into the analysis did not show any notable changes in deep branches of the tree (compare 5 protein vs. 10 protein

concatenation in supplementary figure S2).

Supplementary table S6: Results of IQ-TREE2 "symtest"
The columns of table 6 denote the following concepts:
Protein: Name of the partition, SymSig: Number of significant sequence pairs by test of symmetry, SumNon: Number of non-significant sequence pairs by test of marginal symmetry, SymPval: P-value for maximum test of symmetry, MarSig: Number of significant sequence pairs by test of marginal symmetry, MarNon: Number of non-significant sequence pairs by test of marginal symmetry, MarPval: P-value for maximum test of marginal symmetry, a small p-value means that the assumption of stationarity is rejected. IntSig: Number of significant sequence pairs by test of internal symmetry, Int Non: Number of non-significant sequence pairs by test of internal symmetry, IntPval: P-value for maximum test of internal symmetry, a small p-value means rejection of the assumption of homogeneity.

Protein	Sym-sig	SymNon	SymPval	MarSig	MarNon	MarPval	IntSig	IntNon	IntPval
AHSG	3	26870	0.679029	13	971	nan	1	983	nan
ALB	272	26441	0.347463	0	32	nan	0	32	nan
AMBN	252	26576	0.340511	0	0	nan	0	0	nan
AMELX	0	22213	0.407477	0	0	nan	0	0	nan
AMTN	0	26660	0.587408	0	0	nan	0	0	nan
COL17A1	11	26957	0.406006	727	2561	nan	0	3288	nan
COL1A1	0	26069	0.760846	0	0	nan	0	0	nan
COL1A2	279	26390	0.316152	0	0	nan	0	0	nan
COL2A1	2	26048	0.401605	0	0	nan	0	0	nan
ENAM	1365	25615	0.252049	0	0	nan	0	0	nan
MMP20	0	26631	0.43078	0	0	nan	0	0	nan
ODAM	18	24577	0.0830456	5	127	0.0494201	16	116	0.335447
SERPINC1	47	26347	0.240917	0	0	nan	0	0	nan
TUFT1	0	26652	0.535153	0	60	nan	0	60	nan

For a Bayesian approach, we used PhyloBayes-mpi v.1.9 (Lartillot & Philippe 2004, 2006; Lartillot et al. 2007) to infer a phylogeny from the 14 protein concatenation using the CAT-GTR model:

```
mpirun -np 24 pb_mpi -cat -gtr -d alignment.phylip -x 1 20000 name_of_chain
```

As in the other previously described analyses, tarsiers are placed as a monophyletic group with strepsirrhines (supplementary figure S5). They have similar Robinson-Foulds distances to the reference tree, as all other approaches (supplementary table S7)

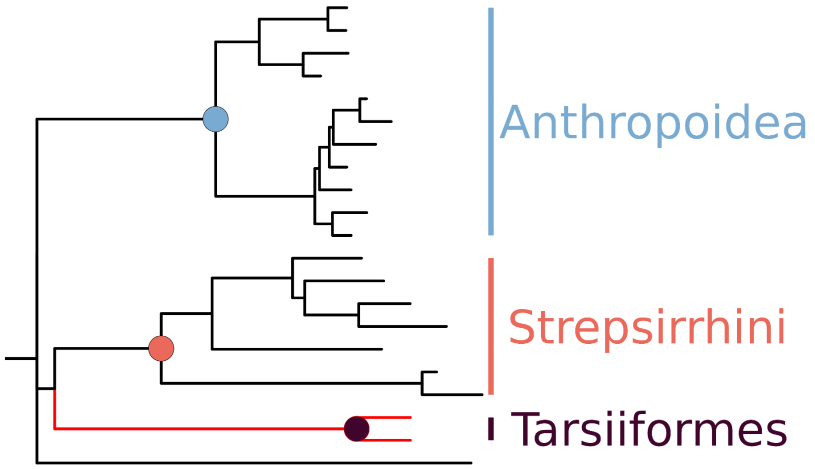

Supplementary figure S5: Placement of tarsiers when using CAT-GTR model in PhyloBayes. The node that splits Strepsirrhini from Tarsiiformes has a posterior probability of 0.87. *Tupaia belangeri chinensis* forms the branch at the bottom. It forms the outgroup to the primates of this tree (short branch length). The tree shown stems from the 14 proteins concatenation.

Supplementary table S7: Robinson-Foulds distances between mixed-model trees and other trees
Abbreviations: SPtree - Species tree (reference tree), ML - Maximum Likelihood (IQ-TREE v.1.6.12), Bay - Bayesian approach, MB - MrBayes, NM - New model Maximum Likelihood (mixture models in IQ-TREE2), PB - PhyloBayes

	SPtree	14ML	14Bay(MB)	10ML	10Bay(MB)	5ML	5Bay(MB)
14NM	110	24	24	60	53	134	123
10NM	122	62	58	20	25	110	101
5NM	176	136	132	118	111	28	51
14PB	108	16	16	52	41	124	109
10PB	116	46	42	18	9	100	85
5PB	143	107	103	95	84	55	22

To test for model misspecification, we performed posterior predictiveness tests with the options implemented in PhyloBayes-mpi:

mpirun -np 4 readpb_mpi -x 100 10 -allppred “name_of_chain”

This simulates alignment data based on the parameter configuration from the model that was estimated using the empirical input data (see phylobayes-mpi 1.9 documentation for more details). Several statistics are then performed on the simulated data which defines null distributions that are then compared to the observed statistic from the empirical input data.

The results show that mean diversity per site, i.e. the mean number of distinct amino acids per site, does not differ significantly between empirical and simulated data (“diversity test”). Also the variance in site-specific empirical amino acid frequencies across the alignment does not differ significantly between empirical and simulated data (“across-site compositional heterogeneity test”). The statistic of the “empirical convergence probability test” can be

interpreted as the long-term probability of any randomly chosen aligned position to converge toward the same state in two independent species. The statistic of the empirical data is significantly different from the mean of the simulated data. The same applies to the compositional homogeneity across taxa (supplementary table S8). Failure of the analysis to adequately model these processes may stem from many factors. These statistics assess properties of cross-species comparisons, which may mean that the species composition of the empirical data is difficult to be modeled correctly. One reason for this might be unequal distribution of closely and distantly related species, with the over-representation of some genera, most of all *Macaca* (15 species), *Trachypithecus* (13 species), *Cercopithecus* (12 species), *Eulemur* and *Plecturocebus* (10 species). These posterior predictive tests in PhyloBase-mpi do not allow for values by partition (since the mixture models do not comprise partitions). Thus, in cases of significant deviations, it cannot be concluded whether a particular protein of the concatenation fails to be modeled adequately. However, the modeling of across-site compositional heterogeneity and in consequence that within and between proteins appeared adequate, which could indicate that it is not single proteins or domains that aggravate training of the model. All these things, considered, together with the results of IQ tree's "symtest", we consider it unlikely that model misspecification explains the incorrect position of tarsiers in all trees that include collagen sequences (supplementary table S5).

diversity test
obs div: 1.71121
mean div: 1.71791 +/- 0.0106828
z-score: 0.627374
pp: 0.260804

empirical convergence probability test
obs: 0.933311
mean: 0.929486 +/- 0.00162032
z-score: 2.36064
pp: 0.00954774

across-site compositional heterogeneity test
obs: 0.0426244
mean: 0.0425281 +/- 9.38781e-05
z-score: 1.02558
pp: 0.137688

max heterogeneity across taxa
obs comp: 0.00102741
mean comp: 0.000502668 +/- 8.4426e-05
z-score: 6.21545
pp: 0

mean squared heterogeneity across taxa (means of values in supplementary table S8)
obs comp: 7.72727e-05
mean comp: 3.5502e-05 +/- 4.84827e-06

z-score: 8.6156
pp: 0

Supplementary table S8: Mean squared heterogeneity across by species.

taxon name	obs	mean pred	z-score	pp
Allenopithecus_nigroviridis	0.0000118731	0.00000775598	1.43977	0.0829146
Allochrocebus_lhoesti	0.00000836821	0.00000690437	0.568129	0.250754
Allochrocebus_preussi	0.00000845323	0.00000793133	0.178173	0.382412
Allochrocebus_solatus	0.0000320659	0.000016592	2.62044	0.0130653
Alouatta_belzebul	0.0000324302	0.0000192012	2.02972	0.0366834
Alouatta_caraya	0.0000137092	0.000014262	-0.105699	0.486432
Alouatta_discolor	0.0000329396	0.0000191236	2.03279	0.0356784
Alouatta_juara	0.0000358857	0.0000186684	2.68745	0.0120603
Alouatta_macconnelli	0.0000379906	0.0000190807	2.8566	0.0105528
Alouatta_nigerrima	0.0000244673	0.0000160394	1.47778	0.080402
Alouatta_palliata	0.0000387599	0.0000201776	2.69095	0.0160804
Alouatta_puruensis	0.0000348974	0.0000187454	2.48278	0.018593
Alouatta_seniculus	0.0000359724	0.0000180463	2.83631	0.0145729
Aotus_azarae	0.0000158291	0.00000987398	1.62473	0.0633166
Aotus_griseimembra	0.0000181559	0.00000957198	2.39331	0.0231156
Aotus_trivirgatus	0.0000171948	0.00000988554	1.99221	0.039196
Aotus_vociferans	0.0000178494	0.00000983935	2.19818	0.0311558
Arctocebus_calabarensis	0.000111077	0.000044958	4.52823	0.000502513
Ateles_belzebuth	0.0000369154	0.0000213182	2.09146	0.0321608
Ateles_chamek	0.0000328752	0.000017215	2.63186	0.0160804
Ateles_geoffroyi	0.0000336951	0.0000169205	2.78039	0.0145729
Ateles_marginatus	0.0000177894	0.000010682	1.7683	0.058794
Ateles_paniscus	0.0000315654	0.0000178874	2.2218	0.0266332
Avahi_laniger	0.0000681207	0.0000367509	2.63908	0.0135678
Avahi_peyrierasi	0.0000784489	0.0000395526	3.17766	0.00552764
Cacajao_ayresi	0.0000997422	0.0000445838	4.38038	0.00100503
Cacajao_calvus	0.000101023	0.0000446519	4.44098	0.00100503
Cacajao_hosomi	0.00028728	0.000131686	5.1889	0
Cacajao_melanocephalus	0.000104398	0.0000460896	4.50278	0.000502513
Callibella_humilis	0.0000319507	0.0000166496	2.43428	0.0226131
Callimico_goeldii	0.0000718757	0.0000309404	3.70728	0.00301508
Callithrix_geoffroyi	0.0000476134	0.000025623	2.23253	0.0296482
Callithrix_jacchus	0.000057315	0.0000483888	0.555327	0.262312
Callithrix_kuhlii	0.0000362972	0.0000218571	1.85576	0.0487437
Carlito_syrichta	0.0000716043	0.0000356565	2.7073	0.0125628
Cebuella_niveiventris	0.0000517149	0.0000161154	6.05027	0
Cebuella_pygmaea	0.0000334335	0.0000181193	2.56511	0.0145729
Cebus_albifrons	0.000097005	0.0000386554	4.67539	0
Cebus_olivaceus	0.000112558	0.0000415974	5.34148	0.000502513
Cebus_unicolor	0.000113792	0.0000435146	5.27868	0
Cephalopachus_bancanus	0.0000788775	0.0000353835	3.28657	0.00552764
Cercocebus_chrysogaster	0.0000221645	0.0000143011	1.74999	0.0497487
Cercocebus_lunulatus	0.0000207324	0.0000142845	1.4637	0.0758794
Cercocebus_torquatus	0.0000232457	0.0000138391	2.18882	0.0261307
Cercopithecus_ascanius	0.0000449259	0.0000206875	3.75723	0.00351759
Cercopithecus_cephus	0.0000544765	0.0000244405	4.11454	0.00201005

Cercopithecus_diana	0.0000491576	0.0000232827	3.76981	0.0040201
Cercopithecus_hamlyni	0.0000126937	0.0000158327	-0.533494	0.669849
Cercopithecus_lowei	0.0000335924	0.0000153629	3.56867	0.00351759
Cercopithecus_mitis	0.0000635299	0.0000247715	5.37466	0.000502513
Cercopithecus_mona	0.0000412565	0.0000186584	3.88156	0.00100503
Cercopithecus_neglectus	0.0000315775	0.0000146362	3.34839	0.00301508
Cercopithecus_nictitans	0.0000373247	0.0000186578	3.22544	0.00603015
Cercopithecus_petaurista	0.0000752806	0.0000310502	4.38669	0.00201005
Cercopithecus_pogonias	0.0000479222	0.0000205292	4.22033	0.00201005
Cercopithecus_roloway	0.0000572111	0.000025718	3.90958	0.00452261
Cheirogaleus_major	0.0000486553	0.0000331279	1.39963	0.0964824
Cheirogaleus_medius	0.00005633	0.0000390783	1.38239	0.0904523
Cheracebus_lucifer	0.000113045	0.0000435124	5.55465	0
Cheracebus_lugens	0.000110748	0.0000440277	5.33894	0
Cheracebus_regulus	0.000111942	0.0000429875	5.57568	0
Cheracebus_torquatus	0.000114523	0.0000431483	5.78092	0
Chiropotes_albinasus	0.00101133	0.000439157	6.42033	0
Chiropotes_israelita	0.000106021	0.000045472	4.71825	0
Chiropotes_sagulatus	0.000124242	0.0000534983	4.75654	0
Chlorocebus_pygerythrus	0.00000870507	0.00000721994	0.556141	0.262312
Colobus_angolensis	0.000007836	0.0000189853	-1.61505	0.978894
Colobus_guereza	0.00000880654	0.0000085616	0.076125	0.418593
Colobus_polykomos	0.00000618165	0.00000771353	-0.525033	0.659296
Daubentonia_madagascariensis	0.000024759	0.0000177874	1.03766	0.140704
Erythrocebus_patas	0.00000956476	0.00000868363	0.280498	0.345729
Eulemur_albifrons	0.0000262639	0.000017454	1.35868	0.0949749
Eulemur_collaris	0.000031946	0.0000177104	2.15931	0.0266332
Eulemur_coronatus	0.000028627	0.0000180792	1.55961	0.0678392
Eulemur_flavifrons	0.0000249639	0.0000171141	1.2152	0.120101
Eulemur_fulvus	0.0000263194	0.0000171189	1.42415	0.0829146
Eulemur_macaco	0.0000258	0.0000174728	1.26305	0.101005
Eulemur_mongoz	0.0000261585	0.0000169479	1.42966	0.0869347
Eulemur_rubriventer	0.0000260128	0.0000176235	1.28813	0.105528
Eulemur_rufus	0.000026347	0.0000179582	1.24134	0.110553
Eulemur_sanfordi	0.000028262	0.000018119	1.49376	0.0768844
Galago_moholi	0.000155907	0.000034719	10.1848	0
Galago_senegalensis	0.00013705	0.000031508	9.73384	0
Galagoides_demidoff	0.000200534	0.0000416285	11.2647	0
Gorilla_beringei	0.00000784413	0.00000830723	-0.145523	0.495477
Gorilla_gorilla	0.0000111053	0.00000986358	0.345556	0.320101
Hapalemur_alaotrensis	0.000158011	0.0000518903	5.67795	0.00351759
Hapalemur_gilberti	0.0000283374	0.0000176013	1.59912	0.0698492
Hapalemur_griseus	0.0000300119	0.0000190934	1.52896	0.0713568
Hapalemur_meridionalis	0.000024864	0.0000168704	1.26342	0.105528
Hapalemur_occidentalis	0.00102741	0.000443298	5.80098	0
Homo_sapiens	0.0000168492	0.0000138287	0.598159	0.244221
Hoolock_hoolock	0.0000182051	0.0000156002	0.45657	0.292965
Hylobates_abbotti	0.0000671277	0.0000540987	0.815524	0.189447
Hylobates_agilis	0.0000152824	0.0000154605	-0.0315322	0.459296
Hylobates_klossii	0.0000433201	0.000027479	1.69863	0.0663317
Hylobates_lar	0.0000198398	0.0000166994	0.512174	0.268342

Hylobates_muelleri	0.0000238805	0.0000197399	0.583774	0.251256
Indri_indri	0.00010843	0.0000454709	4.63382	0.00100503
Lagothrix_lagothricha	0.0000533502	0.0000219324	4.1693	0.00251256
Lemur_catta	0.000034809	0.0000164737	2.91829	0.0140704
Leontocebus_fuscicollis	0.000250744	0.0000864409	6.99467	0
Leontocebus_illigeri	0.000274925	0.0000893723	7.5837	0
Leontocebus_nigricollis	0.000334044	0.0001185	7.06525	0
Leontopithecus_chrysomelas	0.0000460496	0.0000190421	4.22671	0.000502513
Leontopithecus_rosalia	0.0000523452	0.0000204921	4.86689	0.000502513
Lepilemur_ankaranensis	0.0000170405	0.0000269651	-1.00097	0.860302
Lepilemur_dorsalis	0.000119273	0.0000609726	3.40201	0.00552764
Lepilemur_ruficaudatus	0.0000643702	0.0000369562	2.20356	0.0271357
Lepilemur_septentrionalis	0.000120004	0.0000604887	3.5048	0.0040201
Lophocebus_aterrimus	0.0000387623	0.0000187994	3.26302	0.00251256
Loris_lydekkerianus	0.000134653	0.0000285827	10.2172	0
Loris_tardigradus	0.000132713	0.0000288613	9.84247	0
Macaca_arctoides	0.0000135703	0.00000768579	2.13153	0.0306533
Macaca_assamensis	0.0000788595	0.0000889393	-0.333108	0.58794
Macaca_cyclopis	0.0000347452	0.0000146593	4.12447	0.00251256
Macaca_fascicularis	0.0000138113	0.00000873723	1.66735	0.058794
Macaca_fuscata	0.0000148456	0.00000912334	1.77191	0.0517588
Macaca_leonina	0.0000136228	0.00000854725	1.70057	0.058794
Macaca_maura	0.000013422	0.00000993163	0.952178	0.164824
Macaca_mulatta	0.0000123797	0.00000802354	1.51412	0.0809045
Macaca_nemestrina	0.0000152569	0.00000870496	2.15469	0.0276382
Macaca_nigra	0.0000128576	0.00000830578	1.54087	0.0728643
Macaca_radiata	0.000016034	0.00000818001	2.68422	0.0120603
Macaca_siberu	0.0000134077	0.00000873207	1.4403	0.0824121
Macaca_silenus	0.0000142643	0.00000797072	2.21478	0.0256281
Macaca_thibetana	0.00000967729	0.00000767809	0.719412	0.21809
Macaca_tonkeana	0.000017455	0.0000101279	2.13873	0.0286432
Mandrillus_leucophaeus	0.000020737	0.0000116933	2.09604	0.0341709
Mandrillus_sphinx	0.0000174743	0.0000116637	1.39873	0.0939698
Mico_argentatus	0.0000385511	0.0000176807	3.54523	0.0040201
Mico_humeralifer	0.000034628	0.0000170564	3.04803	0.00603015
Mico_spnv	0.0000427368	0.0000146031	5.42535	0.000502513
Microcebus_murinus	0.0000628582	0.0000282686	3.46199	0.00452261
Miopithecus_ogouensis	0.0000553335	0.0000262515	3.73449	0.00452261
Mirza_zaza	0.0000644778	0.0000359267	2.39679	0.019598
Nasalis_larvatus	0.0000194885	0.0000124281	1.65597	0.061809
Nomascus_annamensis	0.0000134419	0.000015449	-0.357448	0.58995
Nomascus_concolor	0.0000684731	0.0000411215	1.77615	0.0532663
Nomascus_gabriellae	0.000020933	0.0000158051	0.878452	0.186432
Nomascus_siki	0.0000155672	0.000015567	0.0000378303	0.451759
Nycticebus_bengalensis	0.000105292	0.0000259737	8.18836	0
Nycticebus_coucang	0.000156637	0.0000431116	7.83421	0
Nycticebus_pygmaeus	0.0000902562	0.0000228463	7.67996	0
Otolemur_crassicaudatus	0.0000916028	0.000025063	7.11858	0
Otolemur_garnettii	0.0000992175	0.0000249913	7.86405	0
Pan_paniscus	0.0000448002	0.0000253806	2.08006	0.0291457
Pan_troglodytes	0.00001084	0.0000101564	0.188589	0.382915

Papio_anubis	0.0000123674	0.00000767285	1.71288	0.0603015
Papio_cynocephalus	0.000014409	0.00000795306	2.26246	0.0261307
Papio_hamadryas	0.0000189376	0.00000969249	2.73897	0.0145729
Papio_kindae	0.0000138412	0.00000833372	1.89645	0.0462312
Papio_papio	0.0000119482	0.00000836697	1.20477	0.125126
Papio_ursinus	0.0000151477	0.00000852986	2.23261	0.0246231
Perodicticus_ibeanus	0.000074999	0.0000389422	2.39338	0.0221106
Perodicticus_potto	0.000226081	0.0000667123	8.06702	0
Piliocolobus_badius	0.000119949	0.0000828633	1.48061	0.0809045
Piliocolobus_gordonorum	0.00000762174	0.0000088728	-0.386192	0.621106
Piliocolobus_kirkii	0.00000313852	0.00000737319	-1.55801	0.977387
Piliocolobus_tephrosceles	0.0000049963	0.00000776032	-0.961143	0.834171
Pithecia_albicans	0.000123039	0.0000557807	4.63469	0.00100503
Pithecia_chrysocephala	0.0000977595	0.0000439736	4.44754	0
Pithecia_hirsuta	0.0000946503	0.000042939	4.31883	0.00100503
Pithecia_mittermeieri	0.000114727	0.0000510349	4.51893	0.000502513
Pithecia_pissinattii	0.000110277	0.0000476967	4.77277	0.000502513
Pithecia_vanzolinii	0.000258117	0.0000983059	6.40891	0
Plecturocebus_bernhardi	0.0000796837	0.0000410148	3.21234	0.00552764
Plecturocebus_brunneus	0.0000748028	0.0000383736	3.19553	0.00452261
Plecturocebus_caligatus	0.0000810236	0.0000409672	3.29681	0.00452261
Plecturocebus_cinerascens	0.0000757824	0.000038266	3.31008	0.00452261
Plecturocebus_cupreus	0.0000828915	0.0000410878	3.52684	0.00301508
Plecturocebus_dubius	0.0000814267	0.000040042	3.55722	0.00251256
Plecturocebus_grovesi	0.0000741048	0.0000369356	3.3388	0.0040201
Plecturocebus_hoffmannsi	0.0000815519	0.0000417763	3.23459	0.00502513
Plecturocebus_miltoni	0.0000775086	0.0000398343	3.24339	0.00452261
Plecturocebus_moloch	0.0000732277	0.0000357735	3.39365	0.00351759
Pongo_abelii	0.0000142675	0.000010648	0.931586	0.163317
Pongo_pygmaeus	0.0000136593	0.00000954128	1.18521	0.11809
Presbytis_comata	0.00000847697	0.00000738806	0.385874	0.299497
Presbytis_mitrata	0.0000082667	0.00000783192	0.151593	0.38392
Prolemur_simus	0.0000303732	0.0000221251	1.045	0.142714
Propithecus_coquereli	0.0000874951	0.0000398374	3.84899	0.00251256
Propithecus_coronatus	0.0000834585	0.0000403621	3.44166	0.00452261
Propithecus_diadema	0.0000914293	0.0000412911	3.96152	0.00201005
Propithecus_edwardsi	0.0000883301	0.0000403583	3.8304	0.00301508
Propithecus_perrieri	0.0000553744	0.0000310874	2.31979	0.0246231
Propithecus_tattersalli	0.0000896273	0.0000417009	3.73998	0.00351759
Propithecus_verreauxi	0.0000256353	0.0000318875	-0.550888	0.679899
Pygathrix_cinerea	0.0000109548	0.00000783885	1.04966	0.142714
Pygathrix_nemaeus	0.0000165499	0.00000985674	1.91359	0.0477387
Pygathrix_nigripes	0.00000990234	0.00000933367	0.165474	0.396985
Rhinopithecus_bieti	0.000895492	0.000407992	5.30115	0
Rhinopithecus_roxellana	0.0000190291	0.00001077	2.23286	0.0271357
Saguinus_bicolor	0.000248501	0.0000839183	7.28786	0
Saguinus_geoffroyi	0.000256528	0.0000872829	7.22083	0
Saguinus_imperator	0.000267581	0.0000995228	6.95351	0
Saguinus_inustus	0.00032533	0.000116077	7.1341	0
Saguinus_labiatus	0.000234748	0.0000792222	7.2716	0
Saguinus_midas	0.000251136	0.0000833256	7.51851	0

Saguinus_mystax	0.000279094	0.0000875529	7.98345	0
Saguinus_oedipus	0.00018958	0.0000700451	6.02146	0
Saimiri_cassiquiarensis	0.000109384	0.000032626	7.05854	0
Saimiri_macrodon	0.000126881	0.0000365671	7.44081	0
Saimiri_oerstedii	0.000103728	0.0000387174	5.0396	0
Saimiri_sciureus	0.000120055	0.0000337994	7.61422	0
Saimiri_ustus	0.000110997	0.0000334477	7.08592	0
Sapajus_apella	0.0000408666	0.0000187055	3.19035	0.00452261
Sapajus_macrocephalus	0.0000319667	0.0000174836	2.29821	0.0221106
Semnopithecus_entellus	0.0000135608	0.0000120775	0.335993	0.338693
Semnopithecus_hypoleucos	0.00000427176	0.00000682055	-0.968338	0.857286
Semnopithecus_johnii	0.0000092478	0.00000949892	-0.0702913	0.477387
Semnopithecus_priam	0.0000317077	0.0000200275	1.70379	0.0567839
Semnopithecus_schistaceus	0.0000139868	0.0000117642	0.516161	0.266834
Semnopithecus_vetulus	0.000011338	0.0000112763	0.0149625	0.443719
Tarsius_lariang	0.000622245	0.000368287	2.91501	0.00552764
Tarsius_wallacei	0.000543937	0.000345882	2.289	0.0190955
Theropithecus_gelada	0.000011595	0.0000073082	1.62926	0.0713568
Trachypithecus_auratus	0.0000133308	0.0000108275	0.665881	0.227136
Trachypithecus_crepusculus	0.0000123819	0.0000104028	0.528317	0.266332
Trachypithecus_cristatus	0.0000118663	0.000010377	0.410855	0.298492
Trachypithecus_francoisi	0.00000742411	0.00000870215	-0.38792	0.61407
Trachypithecus_geei	0.000010023	0.00000894456	0.333786	0.323618
Trachypithecus_germaini	0.0000135123	0.0000100031	0.963819	0.160804
Trachypithecus_hatinhensis	0.0000186508	0.0000129436	1.19578	0.120101
Trachypithecus_laotum	0.0000135129	0.00000943332	1.19957	0.11608
Trachypithecus_leucocephalus	0.0000131924	0.000010111	0.842613	0.185427
Trachypithecus_melamera	0.0000631543	0.0000334338	2.52547	0.019598
Trachypithecus_obscurus	0.00000722797	0.00000757578	-0.122021	0.505025
Trachypithecus_phayrei	0.0000113055	0.00000958599	0.505602	0.269849
Trachypithecus_pileatus	0.00000740775	0.00000977218	-0.691165	0.737186
Tupaia_chiniensis	0.0000185456	0.0000218106	-0.391835	0.613568
Varecia_rubra	0.0000350967	0.0000180801	2.48912	0.0190955
Varecia_variegata	0.0000303854	0.0000174802	1.95702	0.041206

S3. Rate4Site scores and Shannon entropy

The degree of sequence conservation of each of the amino acid sites through the 5, 10 and 14 protein concatenations was estimated in two ways. First, with Shannon entropy with the script Shannon.py (https://gist.github.com/jrjhealey). Given the fact that this metric is agnostic to evolutionary constraints, Rate4Site scores (Pupko et al. 2002) were also calculated. Rate4Site first computes a phylogenetic tree and calculates the rate of each site considering the topology and the length of the branches. Rate4Site was applied through the debian package Rate4Site (https://debian.pkgs.org/10/debian-main-arm64/Rate4Site_3.0.0-6_arm64.deb.html).

For Shannon entropy, the computation was performed on the fasta files of concatenated MSAs. In contrast, for Rate4Site, the computation was made on both, single proteins (used

to divide into variable and conserved datasets) and the concatenated MSA (used for all other figures). Both metrics along the three protein concatenations (5, 10, and 14 proteins) were then normalized to mean of 1. The output files are available at Zenodo (10.5281/zenodo.10637110).

Based on the Rate4Site and Shannon entropy scores, we built files for each of the three concatenations (5, 10 and 14 proteins) by subsetting them by values over and under the threshold of 1. The files with values over this threshold were tagged as “variable”, and the ones with the values under the threshold, as “conserved” (supplementary figure S6).

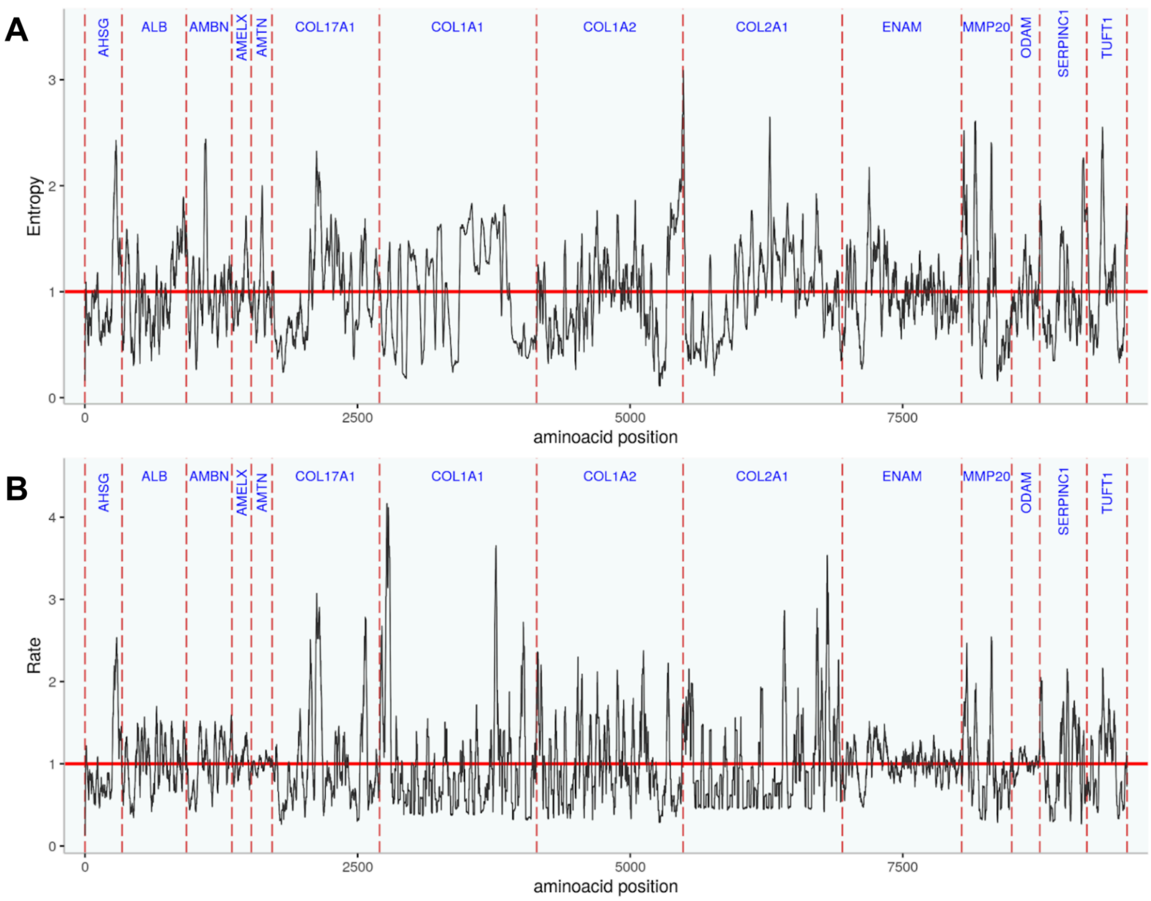

Supplementary figure S6: Division of each protein into “conserved” and “variable” sites
Sites with Shannon entropy or Rate4Site values below 1 are considered “conserved”, sites above this value “variable”. The calculation of these values and normalization has been performed on each protein individually before concatenating the “variable” and “conserved” sites into an MSA, respectively and for each method and concatenation (5, 10, and 14 proteins), which resulted in twelve MSAs. A - by Shannon entropy; B - by Rate4Site score

The result was twelve fasta files. The subsetting was performed through in-house python code. We computed ML phylogenetic analysis over each file with IQ-TREE v1.6.12 through the command:

```
iqtree -nt 4 -s $infile -spp $partfile -alrt 5000 -bb 5000 -nstop 500 -nm 10000 -wsplits -pre $outdir
```

Where the amino acid substitution models used for the analysis for each protein partition was the same as reported in section S2.

We calculated RF-distances for each of the twelve files against the species tree reported in Kuderna et al. (2023) through the function treedist of the R package phangorn (https://cran.r-project.org/web/packages/phangorn/index.html) (supplementary figure S7).

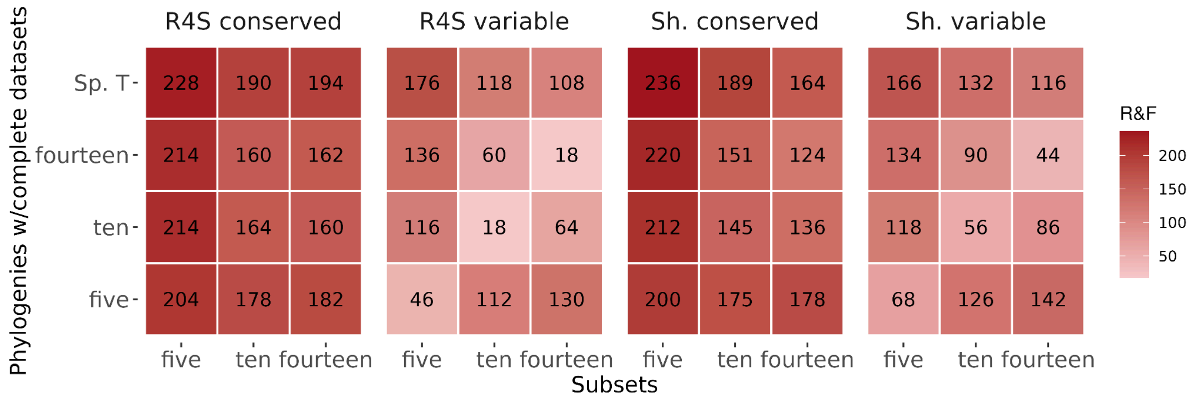

Supplementary figure S7: Robinson-Foulds distances between phylogenies based on highly conserved or variable data and reference species tree
The phylogenies based on variable sites show comparably small RF-distances to the reference tree (Kuderna et al. 2023) (see supplementary figure S3; 5 protein tree: 170, 10 protein tree: 122, 14 protein tree: 108). Especially Rate4Site identifies the sites that drive the topology to resemble the reference. Phylogenies based on the conserved sets of sites tend to show larger RF-distances.

To compare the patterns of sequence conservation of primates, Rate4Site scores were calculated for a set of mammals. The species were chosen in a manner to represent each branch of the mammalian lineage. Species and exact sequences used for the calculations are noted in supplementary table S9. The resulting Rate4Site scores are displayed in supplementary figure S8.

Supplementary table S9: Protein identifiers and species of mammals used to calculate Rate4Site scores
In AMBN and AMELX, a hybrid of *Bos taurus x Bos indicus* was used instead of *Bos taurus* because of better sequence quality.

UniParc ID	Genus	Species Epitheton	Gene
UPI00025DC9FE	*Ictidomys*	*tridecemlineatus*	AHSG
UPI0001F16D78	*Ailuropoda*	*melanoleuca*	AHSG
UPI0003EE3D23	*Leptonychotes*	*weddellii*	AHSG
UPI00029907A1	*Felis*	*catus*	AHSG

UPI001E26DF69	*Lemur*	*catta*	AHSG
UPI000D184883	*Desmodus*	*rotundus*	AHSG
UPI0001C5B6FA	*Loxodonta*	*africana*	AHSG
UPI00045E4D8F	*Orycteropus*	*afer*	AHSG
UPI0003AFC8EB	*Capra*	*hircus*	AHSG
UPI0023FAA5B1	*Ovis*	*aries*	AHSG
UPI0000125015	*Bos*	*taurus*	AHSG
UPI001346041F	*Moschus*	*moschiferus*	AHSG
UPI000BB8DBA5	*Delphinapterus*	*leucas*	AHSG
UPI0003C88453	*Camelus*	*ferus*	AHSG
UPI000333C952	*Echinops*	*telfairi*	AHSG
UPI0000125017	*Homo*	*sapiens*	AHSG
UPI0001FB1964	*Equus*	*caballus*	AHSG
UPI000000191F	*Mus*	*musculus*	AHSG
UPI00038BD484	*Microtus*	*ochrogaster*	AHSG
UPI000328F00F	*Dasypus*	*novemcinctus*	AHSG
UPI0000F63D7F	*Erinaceus*	*europaeus*	AHSG
UPI0003343EAD	*Condylura*	*cristata*	AHSG
UPI00001257C2	*Felis*	*catus*	ALB
UPI0001DECA0B	*Ailuropoda*	*melanoleuca*	ALB
UPI0003EE3118	*Leptonychotes*	*weddellii*	ALB
UPI000D186FF9	*Desmodus*	*rotundus*	ALB
UPI0003346446	*Condylura*	*cristata*	ALB
UPI000004ECF8	*Bos*	*taurus*	ALB
UPI00001257CB	*Ovis*	*aries*	ALB
UPI000BB6C4F8	*Delphinapterus*	*leucas*	ALB
UPI0003C8A000	*Camelus*	*ferus*	ALB
UPI000002C1AC	*Homo*	*sapiens*	ALB
UPI001C5C884A	*Orycteropus*	*afer*	ALB
UPI001E26D6CF	*Lemur*	*catta*	ALB
UPI00001257C3	*Equus*	*caballus*	ALB
UPI0001C5F149	*Loxodonta*	*africana*	ALB
UPI000443926B	*Erinaceus*	*europaeus*	ALB
UPI00000011E3	*Mus*	*musculus*	ALB
UPI00038C3C0B	*Microtus*	*ochrogaster*	ALB
UPI0003CC0A0C	*Dasypus*	*novemcinctus*	ALB
UPI00025DA330	*Ictidomys*	*tridecemlineatus*	ALB
consensus_UPI001343547A_UPI001334EB93	*Moschus*	*moschiferus*	ALB
consensus_UPI0000E43A39_UPI001E1E0F8E	*Echinops*	*telfairi*	ALB

UPI0003AF5A5F	*Capra*	*hircus*	ALB
UPI00025DA2E2	*Ictidomys*	*tridecemlineatus*	AMBN
UPI000C812A7E	*Loxodonta*	*africana*	AMBN
UPI001E267C92	*Lemur*	*catta*	AMBN
UPI00045DB8A8	*Orycteropus*	*afer*	AMBN
UPI00004A0469	*Mus*	*musculus*	AMBN
UPI0003EDC605	*Leptonychotes*	*weddellii*	AMBN
UPI0002AD4D39	*Felis*	*catus*	AMBN
UPI000BB71DA7	*Delphinapterus*	*leucas*	AMBN
UPI00038BF908	*Microtus*	*ochrogaster*	AMBN
UPI0013A62954	*Camelus*	*ferus*	AMBN
UPI001235A039	*Equus*	*caballus*	AMBN
UPI00029D6FC5	*Ovis*	*aries*	AMBN
UPI00059B09E9	*Ailuropoda*	*melanoleuca*	AMBN
UPI0003AFBAE5	*Capra*	*hircus*	AMBN
UPI0003343AF3	*Condylura*	*cristata*	AMBN
UPI0006D8F6B5	*Bos*	*taurus x indicus*	AMBN
UPI00133DC85A	*Moschus*	*moschiferus*	AMBN
UPI000000DCCB	*Homo*	*sapiens*	AMBN
UPI000333E489	*Echinops*	*telfairi*	AMBN
UPI000D184425	*Desmodus*	*rotundus*	AMBN
UPI000328CF7A	*Dasypus*	*novemcinctus*	AMBN
UPI000444297F	*Erinaceus*	*europaeus*	AMBN
UPI00000E705F	*Mus*	*musculus*	AMELX
UPI00038C31DD	*Microtus*	*ochrogaster*	AMELX
UPI0001510A3B	*Ictidomys*	*tridecemlineatus*	AMELX
UPI0000E41C37	*Echinops*	*telfairi*	AMELX
UPI0001C5D780	*Loxodonta*	*africana*	AMELX
UPI000002A3B7	*Homo*	*sapiens*	AMELX
UPI0000F6350E	*Erinaceus*	*europaeus*	AMELX
UPI000D1810BA	*Desmodus*	*rotundus*	AMELX
UPI000057EE88	*Bos*	*taurus x indicus*	AMELX
UPI0006B12AE5	*Capra*	*hircus*	AMELX
UPI00134C2B05	*Moschus*	*moschiferus*	AMELX
UPI00194C6239	*Ailuropoda*	*melanoleuca*	AMELX
UPI000DE7D14B	*Felis*	*catus*	AMELX
UPI00057B080A	*Camelus*	*ferus*	AMELX
UPI00033446DD	*Condylura*	*cristata*	AMELX
UPI001235B980	*Equus*	*caballus*	AMELX
UPI0003EDE479	*Leptonychotes*	*weddellii*	AMELX
UPI001E26D384	*Lemur*	*catta*	AMELX
UPI0000DB81B5	*Ovis*	*aries*	AMELX

UPI00062AA047	*Dasypus*	*novemcinctus*	AMELX
UPI00122CF1E6	*Delphinapterus*	*leucas*	AMELX
UPI00045DD7A1	*Orycteropus*	*afer*	AMELX
H2PUX0	*Pongo*	*abelii*	AMELX
F6PLF2	*Canis*	*lupus*	AMELX
A0A1D5RDA1	*Macaca*	*mulatta*	AMELY
Q99218	*Homo*	*sapiens*	AMELY
Q861X8	*Pan*	*troglodytes*	AMELY
W8CEL7	*Gorilla*	*gorilla*	AMELY
Q99004	*Bos*	*taurus*	AMELY
Q9TUI2	*Equus*	*caballus*	AMELY
Q861X6	*Saimiri*	*sciureus*	AMELY
Q861W9	*Sus*	*scrofa*	AMELY
UPI00064E6D0F	*Echinops*	*telfairi*	AMTN
UPI000BB67AC4	*Delphinapterus*	*leucas*	AMTN
UPI00045DAB7C	*Orycteropus*	*afer*	AMTN
UPI0001C5F2F4	*Loxodonta*	*africana*	AMTN
UPI00059AE37A	*Ailuropoda*	*melanoleuca*	AMTN
UPI00057B3196	*Camelus*	*ferus*	AMTN
UPI0003ACA174	*Equus*	*caballus*	AMTN
UPI00000389F3	*Homo*	*sapiens*	AMTN
UPI0003344074	*Condylura*	*cristata*	AMTN
UPI00134501CB	*Moschus*	*moschiferus*	AMTN
UPI001C2E0034	*Ovis*	*aries*	AMTN
UPI0003F1B638	*Felis*	*catus*	AMTN
UPI0000EBF097	*Bos*	*taurus*	AMTN
UPI000BADC7EA	*Ictidomys*	*tridecemlineatus*	AMTN
UPI0000029DDC	*Mus*	*musculus*	AMTN
UPI0003AFA8CF	*Capra*	*hircus*	AMTN
UPI00238146D9	*Desmodus*	*rotundus*	AMTN
UPI0003EDF5CC	*Leptonychotes*	*weddellii*	AMTN
UPI001E26A062	*Lemur*	*catta*	AMTN
UPI00038C4533	*Microtus*	*ochrogaster*	AMTN
UPI000C837699	*Dasypus*	*novemcinctus*	AMTN
UPI0000F61C6D	*Erinaceus*	*europaeus*	AMTN
UPI0014946839	*Ailuropoda*	*melanoleuca*	COL17A1
UPI00015885B6	*Bos*	*taurus*	COL17A1
UPI0013A681D7	*Camelus*	*ferus*	COL17A1
UPI000846E3F9	*Capra*	*hircus*	COL17A1
UPI000643CF4E	*Condylura*	*cristata*	COL17A1
UPI00062A778E	*Dasypus*	*novemcinctus*	COL17A1
UPI000BB74BB4	*Delphinapterus*	*leucas*	COL17A1

UPI000D1822A7	*Desmodus*	*rotundus*	COL17A1
UPI00064EC0AD	*Echinops*	*telfairi*	COL17A1
UPI000C9E8A63	*Equus*	*caballus*	COL17A1
UPI000443BA49	*Erinaceus*	*europaeus*	COL17A1
UPI001D19C19E	*Felis*	*catus*	COL17A1
UPI000006DB58	*Homo*	*sapiens*	COL17A1
UPI00068077E8	*Ictidomys*	*tridecemlineatus*	COL17A1
UPI001E26B01E	*Lemur*	*catta*	COL17A1
UPI0003EDDE6C	*Leptonychotes*	*weddellii*	COL17A1
UPI000C813626	*Loxodonta*	*africana*	COL17A1
UPI00067CEC6C	*Microtus*	*ochrogaster*	COL17A1
UPI00134886E1	*Moschus*	*moschiferus*	COL17A1
UPI0000475485	*Mus*	*musculus*	COL17A1
UPI001C5CB292	*Orycteropus*	*afer*	COL17A1
UPI0005FAA81D	*Ovis*	*aries*	COL17A1
UPI000C9EBA1E	*Equus*	*caballus*	COL1A1
UPI000003BB1E	*Bos*	*taurus*	COL1A1
UPI0003EE2841	*Leptonychotes*	*weddellii*	COL1A1
UPI0000020B83	*Mus*	*musculus*	COL1A1
UPI0001DEBE30	*Ailuropoda*	*melanoleuca*	COL1A1
UPI000298B65B	*Felis*	*catus*	COL1A1
UPI001345F54E	*Moschus*	*moschiferus*	COL1A1
UPI0005FBADFE	*Ovis*	*aries*	COL1A1
UPI000847268D	*Capra*	*hircus*	COL1A1
UPI0000DACAC3	*Homo*	*sapiens*	COL1A1
UPI00070453D3	*Camelus*	*ferus*	COL1A1
UPI0005406912	*Loxodonta*	*africana*	COL1A1
UPI001A9DA007	*Ictidomys*	*tridecemlineatus*	COL1A1
UPI000BB84A00	*Delphinapterus*	*leucas*	COL1A1
UPI0003336B52	*Echinops*	*telfairi*	COL1A1
UPI001E2667D5	*Lemur*	*catta*	COL1A1
UPI000334613F	*Condylura*	*cristata*	COL1A1
UPI0023812E63	*Desmodus*	*rotundus*	COL1A1
UPI00045E115D	*Orycteropus*	*afer*	COL1A1
UPI0004439EB6	*Erinaceus*	*europaeus*	COL1A1
UPI000328F48D	*Dasypus*	*novemcinctus*	COL1A1
UPI00038BEBF8	*Microtus*	*ochrogaster*	COL1A1
UPI0001DEAD0B	*Ailuropoda*	*melanoleuca*	COL1A2
UPI0000126D35	*Bos*	*taurus*	COL1A2
UPI0003C82C5C	*Camelus*	*ferus*	COL1A2
UPI0003AF943D	*Capra*	*hircus*	COL1A2
UPI00033455AE	*Condylura*	*cristata*	COL1A2

UPI0003292562	*Dasypus*	*novemcinctus*	COL1A2
UPI000BB97DA2	*Delphinapterus*	*leucas*	COL1A2
UPI000D181AB4	*Desmodus*	*rotundus*	COL1A2
UPI000333F1DE	*Echinops*	*telfairi*	COL1A2
UPI0001FB3191	*Equus*	*caballus*	COL1A2
UPI000443F7C7	*Erinaceus*	*europaeus*	COL1A2
UPI000298A177	*Felis*	*catus*	COL1A2
UPI00001B0786	*Homo*	*sapiens*	COL1A2
UPI00038C5333	*Ictidomys*	*tridecemlineatus*	COL1A2
UPI001E26842F	*Lemur*	*catta*	COL1A2
UPI0003EDCB05	*Leptonychotes*	*weddellii*	COL1A2
UPI0001C5C769	*Loxodonta*	*africana*	COL1A2
UPI00038C1845	*Microtus*	*ochrogaster*	COL1A2
UPI001346D826	*Moschus*	*moschiferus*	COL1A2
UPI0000044DC6	*Mus*	*musculus*	COL1A2
UPI00045E253C	*Orycteropus*	*afer*	COL1A2
UPI00029D4E7D	*Ovis*	*aries*	COL1A2
UPI0001F1A265	*Ailuropoda*	*melanoleuca*	COL2A1
UPI000162E87B	*Bos*	*taurus*	COL2A1
UPI0013A67CAE	*Camelus*	*ferus*	COL2A1
UPI000846A11F	*Capra*	*hircus*	COL2A1
UPI0003346F70	*Condylura*	*cristata*	COL2A1
UPI000328E64E	*Dasypus*	*novemcinctus*	COL2A1
UPI000BB74C37	*Delphinapterus*	*leucas*	COL2A1
UPI000D183C63	*Desmodus*	*rotundus*	COL2A1
UPI000333750D	*Echinops*	*telfairi*	COL2A1
UPI0001FB2989	*Equus*	*caballus*	COL2A1
UPI0004439497	*Erinaceus*	*europaeus*	COL2A1
UPI00029890C4	*Felis*	*catus*	COL2A1
UPI0000D79713	*Homo*	*sapiens*	COL2A1
UPI00038C63F9	*Ictidomys*	*tridecemlineatus*	COL2A1
UPI001E266799	*Lemur*	*catta*	COL2A1
UPI0003EE2F09	*Leptonychotes*	*weddellii*	COL2A1
UPI0002233784	*Loxodonta*	*africana*	COL2A1
UPI00038BE84A	*Microtus*	*ochrogaster*	COL2A1
UPI0013431802	*Moschus*	*moschiferus*	COL2A1
UPI000043A9B1	*Mus*	*musculus*	COL2A1
UPI00045E51C7	*Orycteropus*	*afer*	COL2A1
UPI001C2E8633	*Ovis*	*aries*	COL2A1
UPI0004439DC5	*Erinaceus*	*europaeus*	ENAM
UPI0001DEB032	*Ailuropoda*	*melanoleuca*	ENAM
UPI0003EDE520	*Leptonychotes*	*weddellii*	ENAM

UPI000298CC11	*Felis*	*catus*	ENAM
UPI000D184420	*Desmodus*	*rotundus*	ENAM
UPI000D195468	*Capra*	*hircus*	ENAM
UPI0003CD1C6A	*Ovis*	*aries*	ENAM
UPI000FC4D3F9	*Bos*	*taurus*	ENAM
UPI001350ABB9	*Moschus*	*moschiferus*	ENAM
A0A8B8R9K1	*Camelus*	*ferus*	ENAM
UPI0023FBDC02	*Delphinapterus*	*leucas*	ENAM
UPI001E26D3EE	*Lemur*	*catta*	ENAM
UPI000013CE60	*Homo*	*sapiens*	ENAM
UPI000155E1FB	*Equus*	*caballus*	ENAM
UPI0003343607	*Condylura*	*cristata*	ENAM
UPI0001C5F2ED	*Loxodonta*	*africana*	ENAM
UPI00045D6081	*Orycteropus*	*afer*	ENAM
UPI00025DA2DB	*Ictidomys*	*tridecemlineatus*	ENAM
UPI000333824D	*Echinops*	*telfairi*	ENAM
UPI000328B984	*Dasypus*	*novemcinctus*	ENAM
UPI000154CC5B	*Mus*	*musculus*	ENAM
UPI00038BB978	*Microtus*	*ochrogaster*	ENAM
UPI0000000896	*Homo*	*sapiens*	KLK4
UPI001E26E4EA	*Lemur*	*catta*	KLK4
UPI001493E8BC	*Ailuropoda*	*melanoleuca*	KLK4
UPI0003EDF9BD	*Leptonychotes*	*weddellii*	KLK4
UPI001D19BEAC	*Felis*	*catus*	KLK4
UPI000846C44C	*Capra*	*hircus*	KLK4
UPI0005FB8487	*Ovis*	*aries*	KLK4
UPI001333A6C8	*Moschus*	*moschiferus*	KLK4
UPI00017C3809	*Bos*	*taurus*	KLK4
UPI001C2E209E	*Ovis*	*aries*	KLK4
UPI0001FB1F6D	*Equus*	*caballus*	KLK4
UPI001A9F0922	*Ictidomys*	*tridecemlineatus*	KLK4
UPI0004439D51	*Erinaceus*	*europaeus*	KLK4
UPI0000020FE9	*Mus*	*musculus*	KLK4
UPI00038C2237	*Microtus*	*ochrogaster*	KLK4
UPI0003347776	*Condylura*	*cristata*	KLK4
UPI00038C593F	*Ictidomys*	*tridecemlineatus*	MMP20
UPI001E26A080	*Lemur*	*catta*	MMP20
UPI0003AF98CD	*Capra*	*hircus*	MMP20
UPI0005FB8FE4	*Ovis*	*aries*	MMP20
UPI001348900A	*Moschus*	*moschiferus*	MMP20
UPI000012F256	*Bos*	*taurus*	MMP20
UPI0003C89840	*Camelus*	*ferus*	MMP20

UPI000BB801D3	*Delphinapterus*	*leucas*	MMP20
UPI000155E9D4	*Equus*	*caballus*	MMP20
UPI0004444AF6	*Erinaceus*	*europaeus*	MMP20
UPI0003344CA9	*Condylura*	*cristata*	MMP20
UPI001D19FDC5	*Felis*	*catus*	MMP20
UPI0001C5B5D5	*Loxodonta*	*africana*	MMP20
UPI000D186A4F	*Desmodus*	*rotundus*	MMP20
UPI00033371A4	*Echinops*	*telfairi*	MMP20
UPI0014944DE9	*Ailuropoda*	*melanoleuca*	MMP20
UPI00123EADAD	*Leptonychotes*	*weddellii*	MMP20
UPI00038BD861	*Microtus*	*ochrogaster*	MMP20
UPI0000023950	*Mus*	*musculus*	MMP20
UPI00019549AB	*Dasypus*	*novemcinctus*	MMP20
UPI00045D67AA	*Orycteropus*	*afer*	MMP20
UPI000013D0B3	*Homo*	*sapiens*	MMP20
UPI0014947B96	*Ailuropoda*	*melanoleuca*	ODAM
UPI0000EAFE61	*Bos*	*taurus*	ODAM
UPI0013A666AA	*Camelus*	*ferus*	ODAM
UPI0003AFF1F9	*Capra*	*hircus*	ODAM
UPI0003343C2B	*Condylura*	*cristata*	ODAM
UPI0003CC0FFF	*Dasypus*	*novemcinctus*	ODAM
UPI000BB7081C	*Delphinapterus*	*leucas*	ODAM
UPI001E1C196E	*Desmodus*	*rotundus*	ODAM
UPI0003336874	*Echinops*	*telfairi*	ODAM
UPI0001560BCD	*Equus*	*caballus*	ODAM
UPI000443CB73	*Erinaceus*	*europaeus*	ODAM
UPI0009484FF4	*Felis*	*catus*	ODAM
UPI000387D1B9	*Homo*	*sapiens*	ODAM
UPI000BADC9C8	*Ictidomys*	*tridecemlineatus*	ODAM
UPI001E26755C	*Lemur*	*catta*	ODAM
UPI0003EDE888	*Leptonychotes*	*weddellii*	ODAM
UPI0001C5F341	*Loxodonta*	*africana*	ODAM
UPI000F2F2276	*Microtus*	*ochrogaster*	ODAM
UPI0013438182	*Moschus*	*moschiferus*	ODAM
UPI0000217CB9	*Mus*	*musculus*	ODAM
UPI001C5CA009	*Orycteropus*	*afer*	ODAM
UPI0023FD8446	*Ovis*	*aries*	ODAM
UPI0001F17692	*Ailuropoda*	*melanoleuca*	SERPINA1
UPI0000124FD5	*Bos*	*taurus*	SERPINA1
UPI0003C8A3EF	*Camelus*	*ferus*	SERPINA1
UPI0006B11B66	*Capra*	*hircus*	SERPINA1
UPI0003344B0A	*Condylura*	*cristata*	SERPINA1

UPI000328DF56	*Dasypus*	*novemcinctus*	SERPINA1
UPI000BB9AFFB	*Delphinapterus*	*leucas*	SERPINA1
UPI0003340CFE	*Echinops*	*telfairi*	SERPINA1
UPI0000F624DC	*Erinaceus*	*europaeus*	SERPINA1
UPI0002990549	*Felis*	*catus*	SERPINA1
UPI000000CBEC	*Homo*	*sapiens*	SERPINA1
UPI000BADC12A	*Ictidomys*	*tridecemlineatus*	SERPINA1
UPI00123EA0AD	*Leptonychotes*	*weddellii*	SERPINA1
UPI000C81200E	*Loxodonta*	*africana*	SERPINA1
UPI001346A56B	*Moschus*	*moschiferus*	SERPINA1
UPI001C5C8F61	*Orycteropus*	*afer*	SERPINA1
UPI0000124FDF	*Ovis*	*aries*	SERPINA1
UPI00059B1C44	*Ailuropoda*	*melanoleuca*	SERPINC1
UPI0000D89868	*Bos*	*taurus*	SERPINC1
UPI0003C81BE1	*Camelus*	*ferus*	SERPINC1
UPI0003AF37D9	*Capra*	*hircus*	SERPINC1
UPI0003346AD1	*Condylura*	*cristata*	SERPINC1
UPI000329100E	*Dasypus*	*novemcinctus*	SERPINC1
UPI000BB7B261	*Delphinapterus*	*leucas*	SERPINC1
UPI000D17FE07	*Desmodus*	*rotundus*	SERPINC1
UPI001E1DD6E9	*Echinops*	*telfairi*	SERPINC1
UPI001234CC0A	*Equus*	*caballus*	SERPINC1
UPI0000F61FCD	*Erinaceus*	*europaeus*	SERPINC1
UPI001D19D3E2	*Felis*	*catus*	SERPINC1
UPI000002C0C1	*Homo*	*sapiens*	SERPINC1
UPI00025DCD96	*Ictidomys*	*tridecemlineatus*	SERPINC1
UPI001E26AEEC	*Lemur*	*catta*	SERPINC1
UPI0003EDD592	*Leptonychotes*	*weddellii*	SERPINC1
UPI000C813313	*Loxodonta*	*africana*	SERPINC1
UPI00038BE024	*Microtus*	*ochrogaster*	SERPINC1
UPI001343A7C5	*Moschus*	*moschiferus*	SERPINC1
UPI0000000900	*Mus*	*musculus*	SERPINC1
UPI00045E2888	*Orycteropus*	*afer*	SERPINC1
UPI0000125B63	*Ovis*	*aries*	SERPINC1
UPI0001F17B27	*Ailuropoda*	*melanoleuca*	TUFT1
UPI0000136C9D	*Bos*	*taurus*	TUFT1
UPI0003C81F4D	*Camelus*	*ferus*	TUFT1
UPI0003AFE16D	*Capra*	*hircus*	TUFT1
UPI0006428DAE	*Condylura*	*cristata*	TUFT1
UPI00019A5FB8	*Dasypus*	*novemcinctus*	TUFT1
UPI000BB87305	*Delphinapterus*	*leucas*	TUFT1
UPI0023810BC5	*Desmodus*	*rotundus*	TUFT1

UPI00122F45DF	*Echinops*	*telfairi*	TUFT1
UPI0001796107	*Equus*	*caballus*	TUFT1
UPI0004444238	*Erinaceus*	*europaeus*	TUFT1
UPI0005ABE932	*Felis*	*catus*	TUFT1
UPI0000037BFA	*Homo*	*sapiens*	TUFT1
UPI00025DBEA6	*Ictidomys*	*tridecemlineatus*	TUFT1
UPI001E269DF7	*Lemur*	*catta*	TUFT1
UPI0003EE1537	*Leptonychotes*	*weddellii*	TUFT1
UPI0001C5FAD3	*Loxodonta*	*africana*	TUFT1
UPI00038BB892	*Microtus*	*ochrogaster*	TUFT1
UPI00134AC1FD	*Moschus*	*moschiferus*	TUFT1
UPI0000027DA2	*Mus*	*musculus*	TUFT1
UPI001C5CAFE4	*Orycteropus*	*afer*	TUFT1
UPI00100DA457	*Ovis*	*aries*	TUFT1

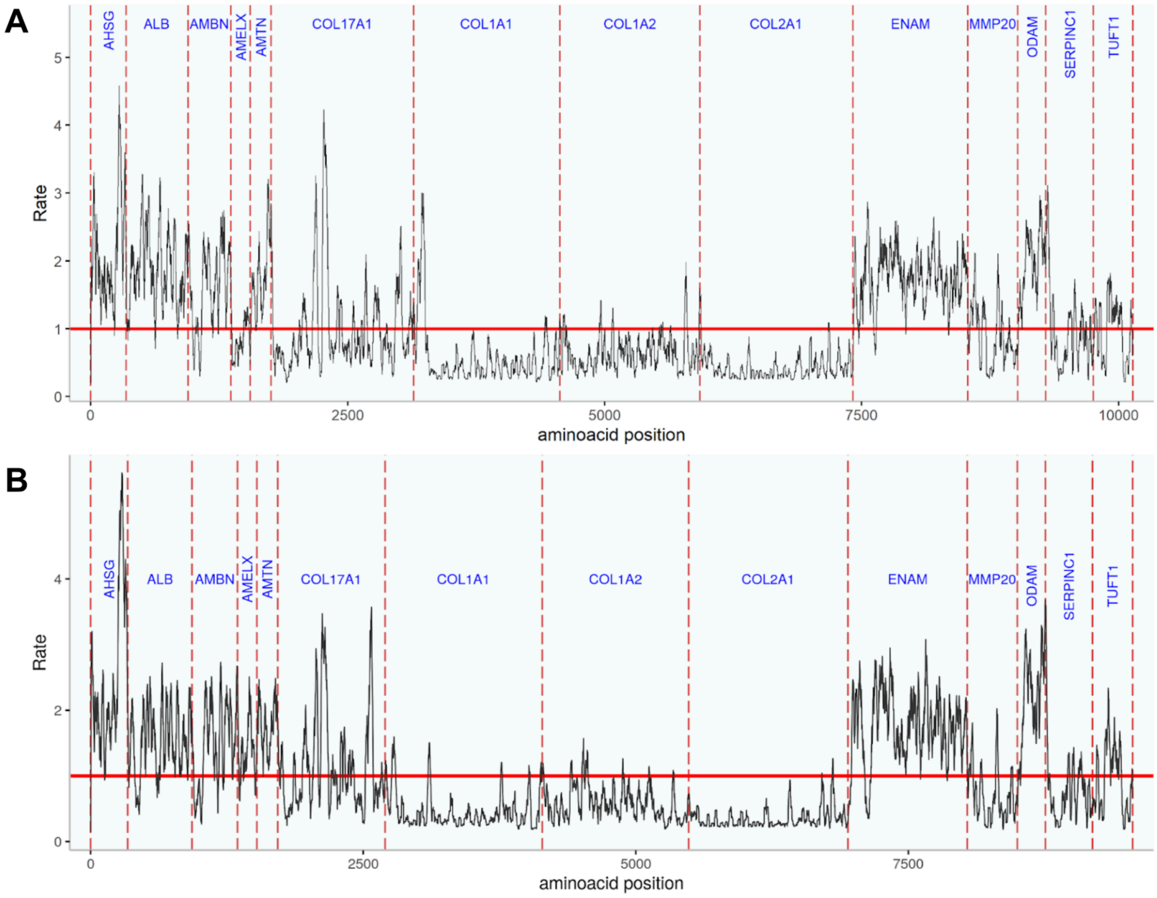

Supplementary figure S8: Rate4Site scores calculated for a set of 22 mammals
A - mammals, B - primates (as in Fig. 2 in main text)

S4. Phylogenetic analyses with simulated fragmentary patterns

The predicted protein sequences were fragmented by removing sites (columns of the MSA) to emulate ancient data (see methods in main text for detailed description). From these fragmented datasets, we calculated phylogenetic trees using ML. The topologies of the resulting trees were compared to the reference tree (Kuderna et al. 2023) and RF-distances measured (see results in main text). The differences between the protein based trees and the reference tree were also visually inspected and are described in supplementary table S10. The names of the fragmentation stages roughly represent the age of data from temperate, subtropical and tropical fossils they are based on in the model.

Supplementary table S10: Differences between protein-based trees and the reference tree (Kuderna et al. 2023) at genus level or higher

MSA (Protein)	Genus	Reference tree	Protein tree	Difference at family level or higher
5 proteins (full length, except signal peptide)	*Galago*	sister to Lorisidae	within Lorisidae	yes
	Otolemur	sister to Lorisidae	within Lorisidae	yes
	Galagoides	sister to Lorisidae	within Lorisidae	yes
	Cebuella	sister to *Mico* + *Callibella*	sister to *Callithrix* + *Mico* + *Callibella*	no
	Semnopithecus	sister to *Trachypithecus*	unresolved with *Trachypithecus*	no
	Lophocebus	sister to *Papio*	sister to *Mandrillus* + *Cercocebus*	no
	Theropithecus	sister to *Papio* + *Lophocebus*	sister to *Papio* + *Lophocebus* + *Mandrillus* + *Cercocebus*	no
	Miopithecus	sister to *Cercopithecus*	sister to all other Cercopithecini	no
	Chlorocebus	sister to *Allochrocebus*	sister to *Cercopithecus* + *Erythrocebus* + *Allenopithecus* + *Allochrocebus*	no
	Allenopithecus	sister to *Allochrocebus* + *Chlorocebus* + *Erythrocebus*	sister to *Allochrocebus*	no
10 proteins (full length, except signal peptide)	*Nasalis*	sister to *Pygathrix* + *Rhinopithecus*	sister to *Pygathrix*	no
	Semnopithecus	sister to *Trachypithecus*	unresolved with *Trachypithecus*	no
	Theropithecus	sister to *Papio* + *Lophocebus*	sister to *Papio*	no
	Miopithecus	sister to *Cercopithecus*	sister to all other Cercopithecini	no
14 proteins (full length, except signal peptide)	*Carlito*	sister to Simiiformes	sister to Strepsirrhini	yes
	Cephalopachus	sister to Simiiformes	sister to Strepsirrhini	yes
	Tarsius	sister to Simiiformes	sister to Strepsirrhini	yes
	Nasalis	sister to *Pygathrix* + *Rhinopithecus*	sister to *Pygathrix*	no
	Lophocebus	sister to *Papio*	sister to *Mandrillus*	no
fragmentation stage 100ka	*Carlito*	sister to Simiiformes	sister to Strepsirrhini	yes
	Cephalopachus	sister to Simiiformes	sister to Strepsirrhini	yes
	Tarsius	sister to Simiiformes	sister to Strepsirrhini	yes

	Cebuella	sister to *Mico* + *Callibella*	sister to *Callithrix*	no
	Lophocebus	sister to *Papio*	sister to *Mandrillus*	no
	Chlorocebus	sister to *Allochrocebus*	sister to *Erythrocebus*	no
	Semnopithecus	sister to *Trachypithecus*	unresolved with *Trachypithecus*	no
	Rhinopithecus	sister to *Pygathrix*	sister to *Semnopithecus* + *Trachypithecus*	no
fragmentation stage 1-2 Ma	*Carlito*	sister to Simiiformes	sister to all other primates	yes
	Cephalopachus	sister to Simiiformes	sister to all other primates	yes
	Tarsius	sister to Simiiformes	sister to all other primates	yes
	Galago	sister to Lorisidae	within Lorisidae	yes
	Otolemur	sister to Lorisidae	within Lorisidae	yes
	Galagoides	sister to Lorisidae	within Lorisidae	yes
	Aotus	sister to Callitrichidae (weak support in reference (PP = 0.56, all other family nodes PP = 1), thus, discordance not informative)	sister to Atelidae + Cebidae + Callitrichidae	yes
	Cebuella	sister to *Mico* + *Callibella*	sister to *Callithrix*	no
	Alouatta	together with other Athelidae sister to Cebidae + Callitrichidae + Aotidae	sister to Callitrichidae	yes
	Ateles	together with other Athelidae sister to Cebidae + Callitrichidae + Aotidae	sister to Callitrichidae	yes
	Lagothrix	together with other Athelidae sister to Cebidae + Callitrichidae + Aotidae	sister to Callitrichidae	yes
	Pan	sister to *Homo*	sister to *Gorilla*	no
	Rhinopithecus	sister to *Pygathrix*	sister to *Semnopithecus* + *Trachypithecus*	no
	Chlorocebus	sister to *Allochrocebus*	sister to *Erythrocebus*	no
	Semnopithecus	sister to *Trachypithecus*	unresolved with *Trachypithecus*	no
	Mandrillus	sister to *Cercocebus*	outgroup to *Papio* + *Theropithecus* + *Lophocebus* + *Cercocebus*	no
	Lophocebus	sister to *Papio*	outgroup to *Theropithecus* + *Papio*	no
fragmentation stage 5 Ma	*Carlito*	sister to Simiiformes	sister to Strepsirrhini	yes
	Cephalopachus	sister to Simiiformes	sister to Strepsirrhini	yes
	Tarsius	sister to Simiiformes	sister to Strepsirrhini	yes
	Varecia	basal-most Lemuridae	sister to *Lemur* + *Prolemur* + *Hapalemur*	no
	Daubentonia	sister to all other Lemuriformes	sister to Indriidae + Cheirogaleidae + Lepilemuridae	yes
	Galagoides	sister to *Otolemur* + *Galago*	within *Galago*	no
	Arctocebus	sister to *Perodictius*	within *Perodictius*	no
	all genera of Platyrrhini	NA	Resolution of nodes at family level is widely in discordance with reference tree.	yes
	all genera of Catarrhini	NA	Still placed in same family as in reference, but relationships within families with low	no

			confidence<br>and mostly in discordance with the reference tree.	
fragmentation stage 10 Ma	all genera of primates	NA	Unresolved phylogeny with bootstrap values typically below 50. Of all infraorders, only Lorisiformes form a monophylum.	yes

S5. Case studies with simulated fragmentary patterns

Four cases were simulated in order to test how fragmentary protein data can be positioned in a phylogenetic tree, the “Neandertal case”, the “Chimpanzee case”, the “Colobine case”, and the “Lemur case”.

Three archaic human individuals with sequence data reduced to fragmentary stage “100 ka” were added to a full-length sequence protein MSA of all 14 proteins of interest. This scaffold MSA consisted of five individuals from all other species of *Hominidae* and one hylobatid outgroup. Accordingly, three *Pan troglodyte*s with sequence data reduced to fragmentary stage “5 Ma” were added to the same complete protein scaffold that lacked *Pan troglodyte*s sequences. The modeling of the fragmentation stages is explained in detail in the methods of the main text.

After adding the fragmentary data to the scaffolds, both datasets were aligned with MAFFT v7.520 through the command:

mafft --auto --addfragments $fragments --keeplength $scaffold > fragmentary_alignment.fasta

ML phylogenetic analysis was performed over the fragmentary alignments with the command:

iqtree -nt 4 –s $fragmentary_alignment.fasta -spp $14_prot.nexus -alrt 5000 -bb 5000 –pre $case_out

The file “14_proteins.nexus” in the associated published dataset (https://zenodo.org/records/10637110) contains the evolutionary models determined for each of the proteins within the concatenation. The resulting trees are displayed in the main text figures 6 and 7.

For the “Colobine case”, also the approach of allowing mixture models was tested using:

iqtree2 -s protein_aligned.fas -m MFP -mset LG+F+G,WAG+F+G,JTT+F+G,GTR20 -madd LG+C20+F+G,LG+C10+F+G,LG+C30+F+G,LG+C40+F+G,LG+C50+F+G,LG+C60+F+G,C10,C20,C30,C40,C50,C60,EX2,EX3,EHO,LG4M,LG4X -nt 8 -bb 1000

This resulted in the following models for each partition:

JTT+G4:AHSG, JTTDCMut+G4:ALB, JTT+G4:AMBN, JTTDCMut+F:AMELX, JTT+F+G4:AMTN, JTT+F+R2:COL17A1, JTTDCMut+F+I:COL1A1, JTTDCMut+F+I:COL2A1, JTT+F+G4:ENAM, JTT+I:MMP20, JTT+I:SERPINC1

In the resulting tree (supplementary figure S9), *Trachypithecus geei* is now placed correctly at genus level, in contrast to the approach reported in the main text (Fig. 7).

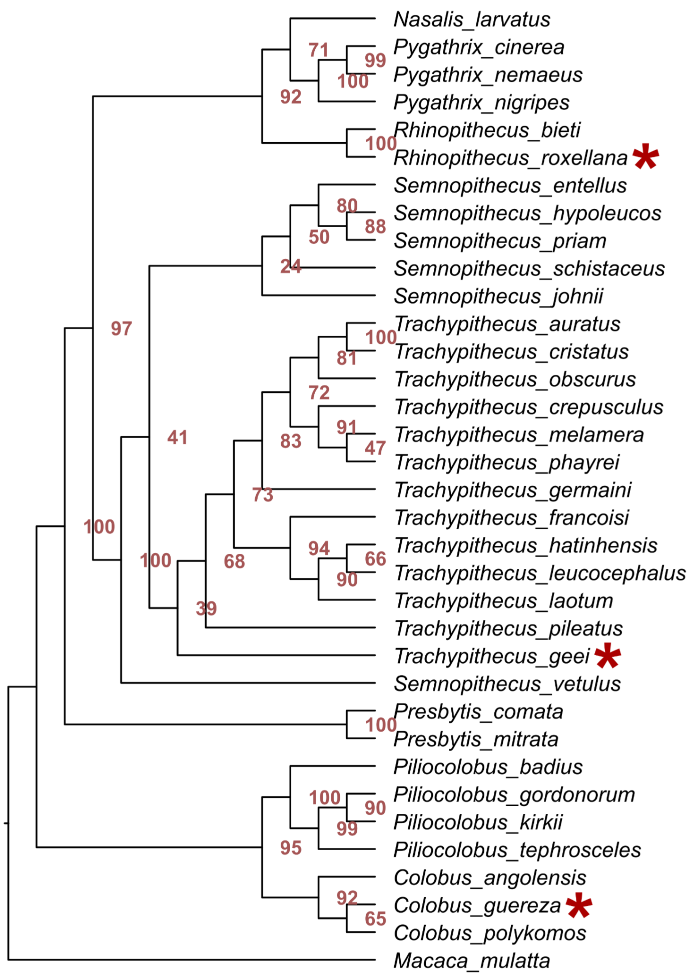

Supplementary figure S9: “Colobine case” with mixture model
All simulated ancient samples (marked with asterisk) are placed correctly at genus level.

In another approach of the “Colobine case”, the topology of the reference sequences was constrained according to the DNA-based reference tree (Kuderna et al. 2023). Only the topology of the simulated ancient samples was not predetermined. This was executed with the following command:

```
iqtree -nt 8 -s $infile -spp $partfile -g $sptree -alrt 5000 -bb 5000 -pre $out
```

In the resulting tree (supplementary figure S10), not only *Trachypithecus geei* is placed correctly at genus level, but also the reference species *Semnopithecus vetulus*. In the approach of the main text (Fig. 7) and the mixture model approach (supplementary figure

S9), *Semnopithecus vetulus* is wrongly placed as an outgroup to all other *Trachypithecus* and *Semnopithecus* individuals.

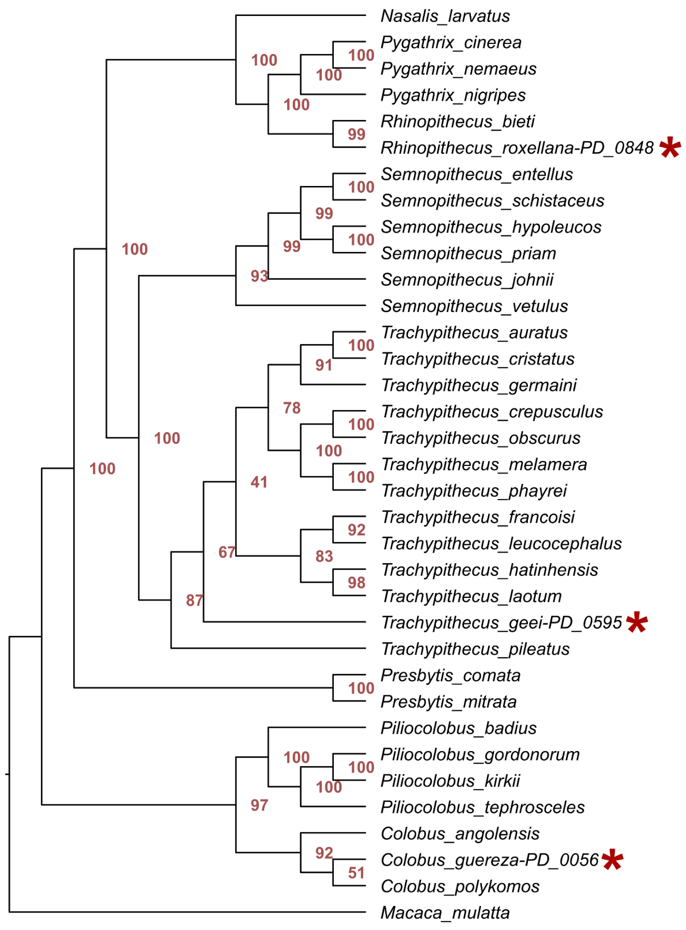

Supplementary figure S10: “Colobine case” with fixed topology
All simulated ancient samples (marked with asterisk) are placed correctly at genus level. Taking into account information from DNA data (Kuderna et al. 2023), all genera of the reference sequences form monophyletic clades.

S6. Divergence time estimation

Divergence times have been estimated using MCMCtree for each node of the “Chimpanzee case” tree (supplementary figure S11, supplementary table S11, supplementary figure S12). The estimation of divergence times was methodologically as similar as possible to the analysis used to generate the reference tree (Kuderna et al. 2023). Consequently, MCMCtree from the PAML program package v. 4.10.6 (Yang 2007) was used for fossil-calibration-based analysis. To format the required protein sequence input, the MSA of the “Chimpanzee case” was pruned to one individual per species, keeping the individuals with the most complete sequences. All fossil calibrations are from publicly available data (de Vries & Beck 2023), and the prior distributions were taken from Kuderna et al. (2023). In the control file of MCMCtree, all parameters were set following “tutorial 4” in the “MCMCtree

tutorials" (dos Reis & Yang 2017) with some modifications to perform an analysis as similar as possible to that of Kuderna et al. (2023): The time scale was set to 1 million years. For the approximate likelihood calculation (usedata=2) under the “correlated rates" clock model (clock=3), we ran 10 replicates of 1 million generations, sampling every 50 generations. 10% were discarded as burn-in. Convergence of runs was confirmed in TRACER v. 1.7.2 (Rambaut et al. 2018). The raw Markov Chain Monte Carlo (MCMC) results (mcmc.txt) were further analyzed in R using the packages “coda” v.0.19.4 (Plummer et al. 2006) and “reshape” v.0.8.9 (Wickham 2007) for calculating a combined 95% highest posterior density (95% HPD). The package “RcolorBrewer” v.1.1.3 (Harrower & Brewer 2003), and the package “ggplot2” v.3.4.3 (Wickham 2016) were used for plotting. Effective priors (prior distribution that takes into account all constraints resulting from all calibrations) were calculated in a separate run (usedata=0).

Consistency of results was tested in ten independent runs (supplementary figure S11). The estimated divergence times were compared to those in the reference tree (Kuderna et al. 2023) obtained by DNA data (supplementary table S11, supplementary figure S12). Most divergence time estimates derived from the protein-based data of the “Chimpanzee case” are younger than those of the genome-based reference (a mean of 21% younger). Only the divergence time estimate of the split between the *Pan troglodytes* (fragmented protein sequences) and *Pan paniscus* (complete protein sequences) is, with a mean of 3.58 Ma, 50% older than that of the reference (Kuderna et al. 2023). The 95% highest posterior density intervals (95% HPD) of all internal nodes overlap between the reference and our case study, with the exception of the split between Ponginae and Homininae (12.24 - 16.42 Ma in our case study, 18.58 - 22.19 Ma in reference). While the boundaries of 95% HPD of this case study are rather close to the effective prior’s 95% HPD boundaries (12.25 - 15.26 Ma), the boundaries of 95% HPD of the reference are completely outside their effective prior 95% HPD bounds (12.26 - 14.58 Ma). This may be a result of genomic data contributing signals that produce a posterior distribution with significantly older ages. These signals, however, may not be reflected in the protein sequences of the enamelome. In addition, the analysis presented here only estimated divergence dates for the superfamily Hominoidea (apes and humans) whereas Kuderna et al. (2023) calculated divergence dates for all primates, which allowed the use of additional fossil calibrations proposed by de Vries and Beck (2023).

In this particular case study, the divergence time estimates were generally younger and closer to the effective prior distribution than those of the reference. This may be a reflection of the relatively high degree of sequence conservation of the enamelome (Bartlett et al. 2006; Al-Hashimi et al. 2009; Haruyama et al. 2011; Castiblanco et al. 2015; Gil-Bona & Bidlack 2020; Warinner et al. 2022). Moreover, the overall short sequence length of all enamelome protein sequences combined is significantly shorter than that of the genomic data as used in the reference (9557 amino acids enamelome MSA vs. 676 kilo-base-pairs reference-MSA of ultra conserved elements). Phylogenetically informative mutations in redundant codon positions or cis-regulatory regions remain invisible in protein sequence data. To fully understand the implications of using the enamelome for divergence time estimates, more comparative studies are needed.

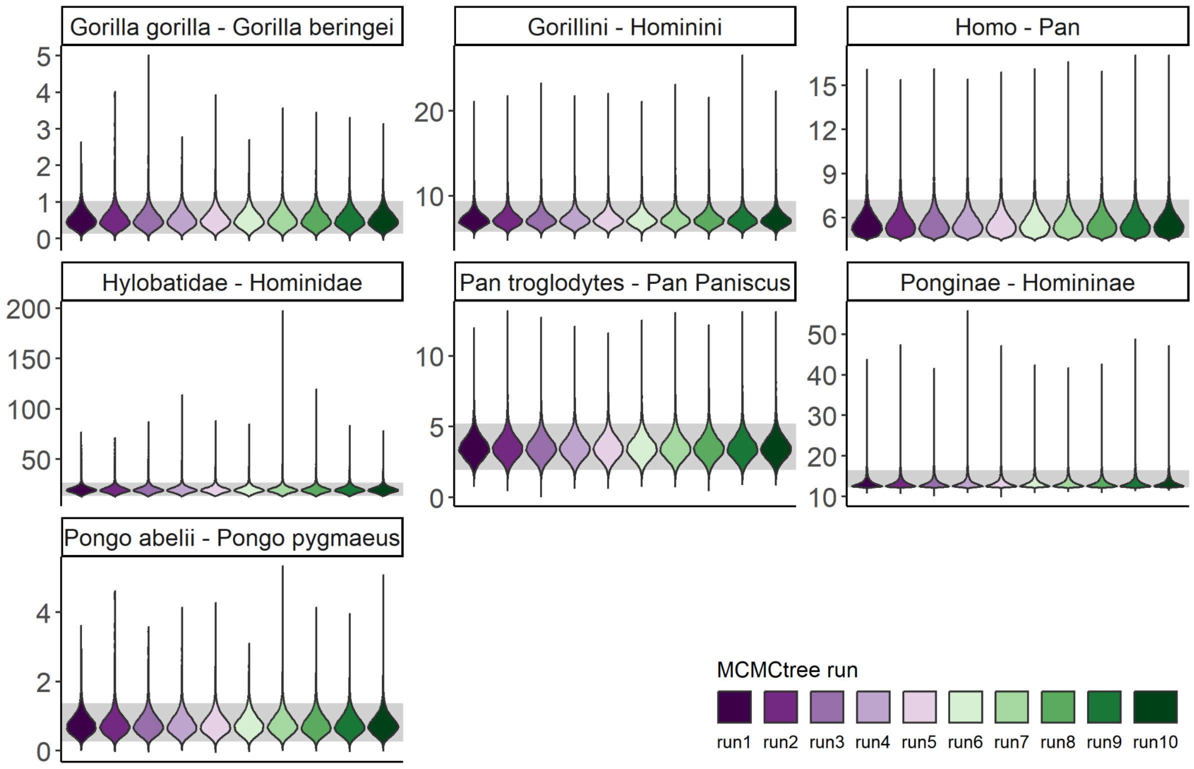

Supplementary figure S11: Densities of node age estimations

For each node the posterior densities of node age estimation is shown for 10 independent runs as violin plots. Note the different y-axis scales. The gray area marks the 95% HPD (highest posterior density) for all 10 runs combined.

Supplementary table S11: Comparison of divergence time estimates at different nodes in Hominidae

Values for reference from Kuderna et al. (2023). "95% HPD posterior" and "Mean protein" from all 10 Markov chain Monte Carlo runs combined. "Protein" refers to the estimations based on protein sequences from Hominidae (*Pan troglodytes* at fragmentation stage "5 Ma"). "Reference" refers to the estimations based on genomic DNA data from Kuderna et al. (2023).

**Split**	**Mean protein**	**95% HPD posterior protein**	**95% HPD effective prior protein**	**Mean reference**	**95% HPD posterior reference**	**95% HPD effective prior reference**	**Mean ratio (protein / reference)**
Hylobatidae - Hominidae	20.07	13.64 - 26.49	13.31 - 30.38	22.89	21.02 - 24.80	13.34 - 21. 30	0.88
Ponginae - Homininae	13.52	12.24 - 16.42	12.25 - 15.26	20.32	18.58 - 22.19	12.26 - 14.58	0.67
Gorillini - Hominini	7.39	5.74 - 9.35	6.62 - 14.62	10.12	8.79 - 11.24	7.50 - 12.18	0.73
Homo - Pan	5.72	4.63 - 7.21	4.63 - 12.87	8.01	6.91 - 8.96	4.63 - 9.41	0.71
Pongo pygmaeus - P. abelii	0.78	0.26 - 1.37	0.03 - 13.22	1.55	1.18 - 1.95	0 - 11.21	0.5
Gorilla gorilla - G. beringei	0.55	0.13 - 1.02	0 - 12.59	1.03	0.81 - 1.26	0 - 9.45	0.53

Pan troglodytes - P. paniscus	3.58	1.94 - 5.23	0 - 10.01	2.39	1.98 - 2.81	0 - 7.05	1.5

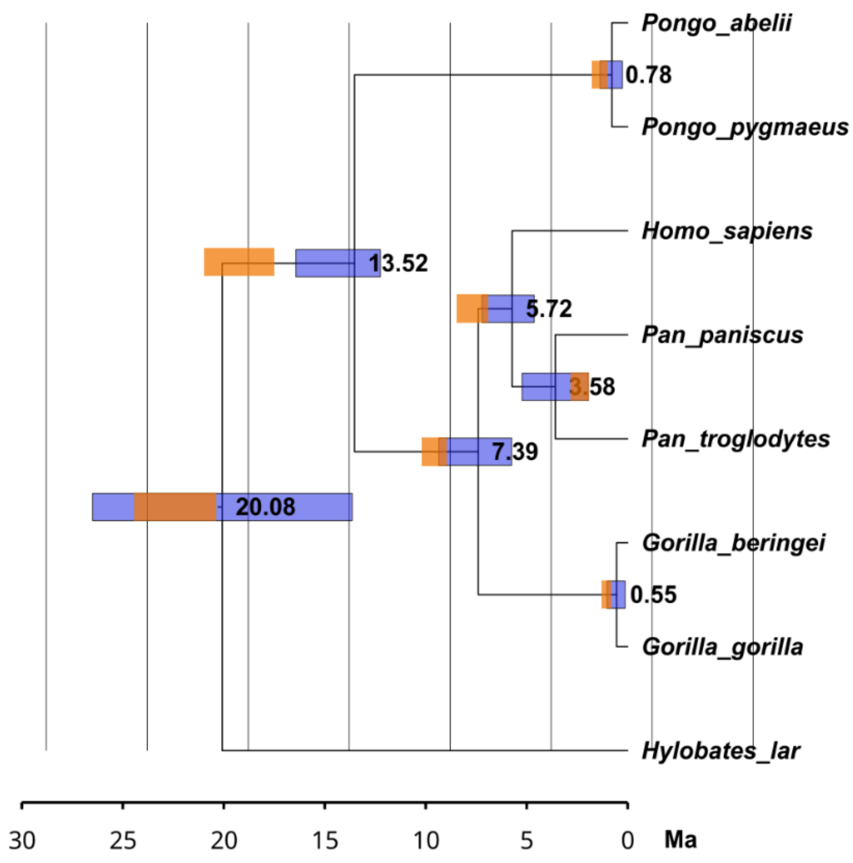

Supplementary figure S12: Phylogeny with divergence time estimates for "Chimpanzee case" simulation
The X-axis represents "million years ago". Purple bars display 95% HPD of all 10 MCMCtree runs combined. Orange bars display the 95% HPD of the estimates based on genomic data from the reference tree (Kuderna et al. 2023). Nearly all 95% HPD are overlapping with the ones of the reference tree, including the simulated ancient *Pan troglodytes* sequence. Only the 95% HPD of the split between Ponginae and Homininae covers a younger time range and does not overlap with that of the reference tree (12.24 - 16.42 Ma in our case study, 18.58 - 22.19 Ma in reference).

References

Al-Hashimi N, Sire JY, Delgado S. 2009. Evolutionary analysis of mammalian enamelin, the largest enamel protein, supports a crucial role for the 32-kDa peptide and reveals selective adaptation in rodents and primates. J. Mol. Evol. 69:635–656.

Bartlett JD et al. 2006. Protein-Protein Interactions of the Developing Enamel Matrix. Curr. Top. Dev. Biol. 74:57–115.

Capella-Gutiérrez S, Silla-Martínez JM, Gabaldón T. 2009. trimAl: a tool for automated alignment trimming in large-scale phylogenetic analyses. Bioinformatics. 25:1972–1973.

Castiblanco GA et al. 2015. Identification of proteins from human permanent erupted enamel. Eur. J. Oral Sci. 123:390–395.

Chiva C et al. 2018. QCloud: A cloud-based quality control system for mass spectrometry-based proteomics laboratories. PLoS One. 13:e0189209.

Gil-Bona A, Bidlack FB. 2020. Tooth enamel and its dynamic protein matrix. Int. J. Mol. Sci. 21:1–25.

Harrower M, Brewer CA. 2003. ColorBrewer.org: An online tool for selecting colour schemes for maps. Cartogr. J. 40:27–37.

Haruyama N, Hatakeyama J, Moriyama K, Kulkarni AB. 2011. Amelogenins: Multi-Functional Enamel Matrix Proteins and Their Binding Partners. J. Oral Biosci. 53:257–266.

Kalyaanamoorthy S, Minh BQ, Wong TKF, von Haeseler A, Jermiin LS. 2017. ModelFinder: fast model selection for accurate phylogenetic estimates. Nat. Methods. 14:587–589.

Kuderna LFK et al. 2023. A global catalog of whole-genome diversity from 233 primate species. Science. 380:906–913.

Lartillot N, Brinkmann H, Philippe H. 2007. Suppression of long-branch attraction artefacts in the animal phylogeny using a site-heterogeneous model. 14:1–14.

Lartillot N, Philippe H. 2004. A Bayesian mixture model for across-site heterogeneities in the amino-acid replacement process. Mol. Biol. Evol. 21:1095–1109.

Lartillot N, Philippe H. 2006. Computing Bayes factors using thermodynamic integration. Syst. Biol. 55:195–207.

Li H. 2011. A statistical framework for SNP calling, mutation discovery, association mapping and population genetical parameter estimation from sequencing data. Bioinformatics. 27:2987–2993.

Li H et al. 2009. The Sequence Alignment/Map format and SAMtools. Bioinformatics. 25:2078–2079.

Minh BQ et al. 2020. IQ-TREE 2: New models and efficient methods for phylogenetic inference in the genomic era. Mol. Biol. Evol. 37:1530–1534.

Naser-Khdour S, Minh BQ, Zhang W, Stone EA, Lanfear R. 2019. The prevalence and impact of model violations in phylogenetic analysis. Genome Biol. Evol. 11:3341–3352.

Nguyen L-T, Schmidt HA, von Haeseler A, Minh BQ. 2015. IQ-TREE: a fast and effective stochastic algorithm for estimating maximum-likelihood phylogenies. Mol. Biol. Evol. 32:268–274.

Plummer M, Best N, Cowles K, Vines K. 2006. {CODA}: Convergence Diagnosis and Output Analysis for {MCMC}. R News. 6:7–11.

Pupko T, Bell RE, Mayrose I, Glaser F, Ben-Tal N. 2002. Rate4Site: An algorithmic tool for the identification of functional regions in proteins by surface mapping of evolutionary determinants within their homologues. Bioinformatics. 18:S71–S77.

Rambaut A, Drummond AJ, Xie D, Baele G, Suchard MA. 2018. Posterior Summarization in Bayesian Phylogenetics Using Tracer 1.7. Syst. Biol. 67:901–904.

dos Reis M, Yang Z. 2017. MCMCTree tutorials. http://abacus.gene.ucl.ac.uk/software/MCMCtree.Tutorials.pdf (last accessed February 2024).

Ronquist F, Huelsenbeck JP. 2003. MRBAYES 3: Bayesian phylogenetic inference under mixed models. Bioinformatics. 19:1572–1574.

de Vries D, Beck RMD. 2023. Twenty-five well-justified fossil calibrations for primate divergences. Palaeontol. Electronica. 26:1–52.

Warinner C, Korzow Richter K, Collins MJ. 2022. Paleoproteomics. Chem. Rev. 122:13401–13446.

Wickham H. 2016. *Ggplot2: Elegant graphics for data analysis*. 2nd ed. Springer International Publishing: Cham, Switzerland.

Yang Z. 2007. PAML 4: phylogenetic analysis by maximum likelihood. Mol. Biol. Evol. 24:1586–1591.
